# Real Time Classification of Viruses in 12 Dimensions

**DOI:** 10.1371/journal.pone.0064328

**Published:** 2013-05-22

**Authors:** Chenglong Yu, Troy Hernandez, Hui Zheng, Shek-Chung Yau, Hsin-Hsiung Huang, Rong Lucy He, Jie Yang, Stephen S.-T. Yau

**Affiliations:** 1 Department of Mathematics, Statistics and Computer Science, University of Illinois at Chicago, Chicago, Illinois, United States of America; 2 Information Technology Services Center, Hong Kong University of Science and Technology, Kowloon, Hong Kong; 3 Department of Biological Sciences, Chicago State University, Chicago, Illinois, United States of America; 4 Department of Mathematical Sciences, Tsinghua University, Beijing, P. R. China; Institute of Infectious Disease and Molecular Medicine, South Africa

## Abstract

The International Committee on Taxonomy of Viruses authorizes and organizes the taxonomic classification of viruses. Thus far, the detailed classifications for all viruses are neither complete nor free from dispute. For example, the current missing label rates in GenBank are 12.1% for family label and 30.0% for genus label. Using the proposed Natural Vector representation, all 2,044 single-segment referenced viral genomes in GenBank can be embedded in 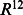. Unlike other approaches, this allows us to determine phylogenetic relations for all viruses at any level (e.g., Baltimore class, family, subfamily, genus, and species) in real time. Additionally, the proposed graphical representation for virus phylogeny provides a visualization of the distribution of viruses in 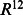. Unlike the commonly used tree visualization methods which suffer from uniqueness and existence problems, our representation always exists and is unique. This approach is successfully used to predict and correct viral classification information, as well as to identify viral origins; e.g. a recent public health threat, the West Nile virus, is closer to the Japanese encephalitis antigenic complex based on our visualization. Based on cross-validation results, the accuracy rates of our predictions are as high as 98.2% for Baltimore class labels, 96.6% for family labels, 99.7% for subfamily labels and 97.2% for genus labels.

## Introduction

The rapid development of sequencing technologies produces a large number of viral genome sequences. Characterizing genetic sequences and determining viral origins have always been important issues in virology [1]. The study of sequence similarity at the interfamily level is especially crucial for revealing key aspects of evolutionary history [2]. It is known that the commonly used multiple sequence alignment methods fail for diverse systems of different families of RNA viruses [3]. Another popular category of alignment-free methods is based on the statistics of oligomers frequency and associated with a fixed length segment, known as *k*-mers [4]. In the past 10 years alignment-free methods have attracted a lot of attention from researchers [5]–[8]. More recently the genome space method has been shown to be a fast and efficient way to characterize nucleotide sequences [9], [10]. Unlike *k*-mer methods, which ignore the positional information of nucleotides, the natural vector characterization constructs a one-to-one correspondence between genome sequences and numerical vectors [10]. Along this line, we construct a viral genome space in 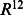 based on the quantity and global distribution of nucleotides in viral sequences. Each sequence is uniquely represented by a single point in 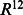, called a Natural Vector (NV). The Euclidean distance between two points represents the biological distance of the corresponding two viruses. This allows us to make a simultaneous comparison against all available viruses at any level (e.g., Baltimore class, family, subfamily, genus, and species) in a fast and efficient manner. Using a higher dimensional NV doesn't change the classification or phylogenetic relationships. We emphasize that our NV does not depend on any model assumption. Our approach to classifying viral genomes is not a partial-sequence-based method; it uses the global sequence information of genomes. Furthermore, we propose a two-dimensional graphical representation of viruses in the genome space which is unique and does not depend on any model assumption.

## Materials and Methods

### Overview of the viral genome data

The composition and structure of viral genomes is more varied than bacterial, plant, or animal kingdoms. The viral genomes may be single-stranded or double-stranded, linear or circular, and in single-segmented or multi-segmented configuration. There are 2,418 reference viral genomes in the current GenBank collection (up to 2012-4-6). In this study, we focus on the 2,044 single-segment viruses among them (Table S1 in File SI). Baltimore classification places viruses into one of seven groups based on their method of viral mRNA synthesis [11]. The International Committee on Taxonomy of Viruses (ICTV) has also developed a universal taxonomic scheme for viruses by assigning them order, family, subfamily, genus, and species [12]. All viruses belonging to the same family should have the same Baltimore classification. After checking the consistency between Baltimore classification and ICTV families, we find that the original GenBank records of the viruses in the *Retroviridae* family (RNA viruses) contain erroneous DNA label information. Additionally, there are 17 families containing both circular virus(es) and linear virus(es) (Table S2 in File SI). This is not possible based on the ICTV classification criteria [12]. Using within-family majority voting results in the correct shape labels. In Table 1, we show the corrected Baltimore classification information of the 2,044 single-segment referenced viruses. Satellites have no Baltimore class and <NA> refers to unknown classifications.

**Table 1:** The dataset and statistical results of our study.

Baltimore class		I	II	III	IV	V	VI	VII	Satellite	<NA>
Name		dsDNA	ssDNA	dsRNA	ssRNA(+)	ssRNA(−)	ssRNA(RT)	dsDNA(RT)		
Linear number		599	56	45	563	66	58	0	33	19
Circular number		177	272	0	0	1	0	44	103	8
Total Number		776	328	45	563	67	58	44	136	27
Checking Baltimore classification by NV	Inconsistencies	4	14	5	21	2	7	1	NA	NA
	Inconsistency Rate	0.01	0.04	0.11	0.04	0.03	0.12	0.02	NA	NA
Checking Family classification by NV	Inconsistencies	58	0	0	11	0	0	0	NA	NA
	Inconsistency Rate	0.08	0	0	0.02	0	0	0	NA	NA

(1) The corrected Baltimore classification information of the 2,044 single-segmented referenced viruses. (2) The Baltimore classification prediction information for the 2,044 viruses. (3) The family classification prediction information given Baltimore class information.

Among the 2,044 viruses, there are 248 without family labels; a missing label rate 12.1%. The remaining viruses belong to 72 families. The missing label rates for subfamily and genus are 84.7% and 30.0%, respectively. Relative to the dramatically increased rate of virus genome sequencing, the expert time and technical resources of ICTV are too restricted to be able to continue assigning labels to all new sequences. For details of the dataset, please see Supporting Information.

### Natural vector and genome space

To study virus classification and phylogeny rapidly and accurately, we construct a novel viral genome space as a subspace in 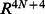 (*N*≥2) by means of the natural vector mapping which is based on the quantity and global distribution of nucleotides in the sequence. Each sequence is uniquely represented by a single point in this subspace. The Euclidean distance between two points represents the biological distance of the corresponding two viruses. Using the natural vector representation we can perform phylogenetic and cluster analysis for all the existing viral genomes.

A key finding of this work is that this viral genome space is a 12-dimensional space (*N* = 2). We emphasize that our natural vectors depend only on the numbers and distributions of nucleotides in the viral genome sequences. They do not rely on any model assumption. There are two reasons that the virus is represented as a point in the viral genome space without losing inherent biological information. First, the 12-dimensional natural vector mapping on all the viruses we examined is one-to-one. Second, we do not gain any more useful information for classification purposes using the 16-dimensional or higher natural vector mapping. Our new approach to classifying viral genomes is not a partial-sequence-based method. It is constructed based on the global sequence information of genomes.

Let 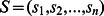 be a nucleotide sequence of length *n*, that is, 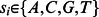, *i* = 1, 2, …, *n*. For *k* = *A*, *C*, *G*, *T*, define 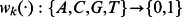 such that 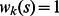 if *s* = *k* and 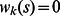 otherwise.

1. Let 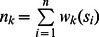 denote the number of letter *k* in *S*.
2. Let 
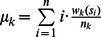
 be the mean position of letter *k*.
3. For *j* = 2, 3, …, 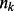, let 
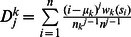
.

The natural vector *N(S)* of a nucleotide sequence *S* is defined by
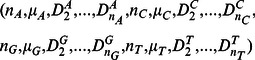
(A1)It has been proved that the correspondence between nucleotide sequences and their associated natural vectors is one-to-one [10]. The natural vector defined here is essentially the same vector defined in [10] when 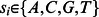 only.

In DNA/RNA sequencing data, the standard International Union for Pure and Applied Chemistry (IUPAC) nucleotide code is used to describe ambiguous sites, where a single character may represent more than one nucleotide (see Table S3 in File SI). Our natural vector defined above can be easily extended to handle the nucleotide sequences with ambiguous letters other than *A*, *C*, *G*, *T*. That is, for *k* = *A*, *C*, *G*, *T*, let the weight 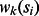 be the expected count of letter *k* at position *i*. For example,
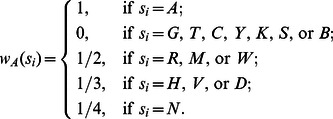
For a DNA/RNA sequence with ambiguous letters, the coordinates of weighted natural vector are defined by the same formula as in (A1) which is also used for the usual natural vector.

We use this natural vector to construct a viral genome space, which is a moduli space of viral genomes. In this space each point corresponds to a viral genome. The distance between two viruses in the space reflects the biological distance between these two viruses. For a viral genome sequence of length *n*, we can compute its (*n*+4)-dimensional natural vector as in (A1). A noteworthy contribution of this work is that we do not need to compute the central moments (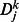) higher than *j* = 2 in the vector since the 12-dimensional natural vectors have allowed us to obtain stable classification results - the 12-dimensional natural vector mapping (restricted to this dataset) is still one-to-one. Thus, we use the 12-dimensional natural vector
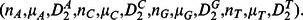
Using these natural vectors, we can construct the genome space as a subset in 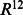. Every virus corresponds to a point in this space. Using the Euclidean distance between two points as a metric, we can perform phylogenetic and clustering analysis for the viral genome sequences.

### Novel graphical representation for phylogeny

Distance matrices or similarity matrices are used by many algorithms [13]–[15] to produce either rooted or unrooted phylogenetic trees of DNA or protein sequences. For example, the neighbor-joining algorithm [15] produces unrooted trees, while the UPGMA algorithm [13] produces rooted trees. Additionally, the matrices produced by sequence alignment methods may not satisfy the triangle inequality and therefore are not proper distance matrices. Even if a proper distance matrix and an algorithm are given, the resulting trees may not be unique [16], [17]. Therefore previous phylogenetic results may be controversial.

With the construction of a natural vector distance matrix we propose a natural graphical representation to overcome the disadvantages of existing methods for inferring phylogenies. Specifically, given a distance matrix of finite elements, the algorithm is as follows:

1. For each point *A*, find the closest point(s) *B* (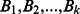) to *A*. Then connect *A* to *B* (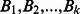) with a directed line(s) from *A* to *B*. If both *A* and *B* are closest to each other then connect them using a bi-directional line.
2. We then get many connected components, called level-1 graphs, after step (1). We compute the distance matrix for these connected components. The distance between two components is defined as the minimum of all distances between an element in one component and an element in another component. We then obtain a new distance matrix, in which the elements are the connected graphs obtained in step (1).
3. Repeat the process in steps (1) and (2) to obtain higher-level graphs until we get one connected component for all elements, which is the final graphical representation.

**Figure 1:**
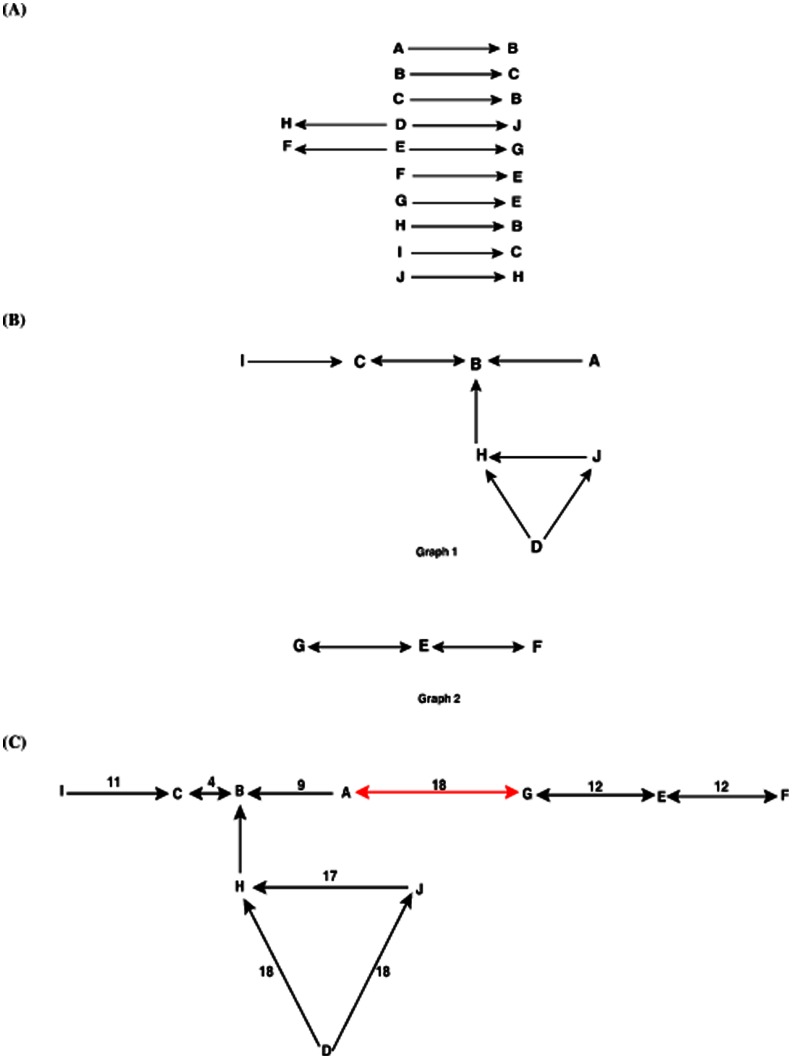
The graph construction process of a distance matrix shown in Table 2. (A) From each element draw a directed line(s) to its closest element(s). (B) Combine the connected elements in (A) using directed lines, resulting in two connected graphs, graph 1 and graph 2. (C) The final graphical representation is obtained by connecting element A in graph1 and element G in graph2, based on the distance matrix in Table 2.

For example, given the distance matrix of 10 elements in Table 2, we illustrate the graph construction process in Figure 1. First, we find the closest element(s) for each element and connect them as shown in Figure 1(A). Then we combine the level-1 connected components to get level-2 components, graph 1 and graph 2, as shown in Figure 1(B). We check the minimum distance between these two graphs, and get the new distance matrix in Table 3. The minimum distance 18 is obtained between element *A* in graph 1 and element *G* in graph 2. So, we connect these two elements to get a connected graph as shown in Figure 1(C). We use the directed red line to mark this connection, indicating 2nd level connection. Clearly, this directional graphical representation uniquely illustrates the 1st-nearest-neighbor relationships.

**Table 2:** The distance matrix of 10 elements.

	A	B	C	D	E	F	G	H	I	J
A	0									
B	9	0								
C	13	4	0							
D	23	21	23	0						
E	27	34	38	30	0					
F	26	36	39	39	12	0				
G	18	26	30	25	12	16	0			
H	19	8	9	18	34	25	25	0		
I	20	14	11	30	43	44	35	12	0	
J	28	21	20	18	20	47	37	17	20	0

**Table 3:** The distance matrix of 2 graphs obtained from Figure 1.

	Graph 1	Graph 2
Graph 1	0	
Graph 2	18	0

The direction in the graph shows the closest element(s) to each element based on their biological distances. For example, given a virus *A*, virologists would like to know which virus *B* is closest to *A*. An arrow from *A* to *B* in the graph represents this relation. Here we need to point out that the natural graphical representation is not necessarily a tree. As in the example, a cycle may exist in the graphical representation which may show interesting biological information.

## Results

### Predict Baltimore class label

For each virus we find its nearest neighbor in the 12-dimensional NV genome space and check whether its label matches that of its nearest neighbor. If we have a complete genome space which contains all of the viruses it is reasonable to assume any virus must have a neighbor sharing the same label.

Firstly, given a nucleotide sequence along with its topological information (DNA/RNA, single/double-stranded, linear/circular), we can use our method to predict its Baltimore class label. For a single-stranded DNA sequence or double-stranded RNA sequence, there is no need to predict since it exactly belongs to class II (ssDNA) or III (dsRNA), respectively. For a double-stranded DNA sequence, it may belong to class I (dsDNA) or VII (dsDNA (RT)). For a single-stranded RNA sequence, it may belong to class IV (ssRNA (+)), V (ssRNA (−)), or VI (ssRNA (RT)). Here we use “#virus” to denote the number of viruses, and “#error” to denote the number of viruses with inconsistent nearest neighbor labels. Then the inconsistency rate is defined by #error/#virus ×100%. For classes I and VII, the inconsistency rate for linear viruses is 0/599 = 0% since all class VII viruses are circular, and the inconsistency rate for circular viruses is 3/(177+44) = 3/221 = 1.36%. For classes IV, V, and VI, the inconsistency rate for linear viruses is 45/(563+66+58) = 45/687 = 6.55%, and the inconsistency rate for circular viruses is 0/1 = 0%. Thus the overall inconsistency rate is (3+45)/(599+221+687+1) = 3.18%.

The reason for the inconsistency is due to the sparsity of the reference dataset, i.e., the dataset in this study does not cover all of the viruses. In this case, prediction based on a distant neighbor is not reliable. For example, the distance between virus #170 (class I) and its nearest neighbor virus #62 (class VII) is 1265.021. To measure the relative magnitude of 1265.021, we collect all the distances D(A) between virus A in class VII and its nearest neighbor within class VII. Then (1265.021−maximum)/(Q3−Q1) = 2.651, where “maximum”, “Q3”, “Q1” are the maximum, .75th quantile, and .25th quantile of the collection of D(A)'s, and equal to 808.147, 320.170, 147.861, respectively. Such a big relative distance indicates that the distance from virus #170 to its nearest neighbor virus # 62 is much bigger than all the nearest distances of the viruses belonging to class VII. The prediction that virus #170 belongs to class VII based on the label of virus #62 is thus unreliable.

In practice, for each class, we collect the nearest distance for each virus within the class, and get the .75 quantile of those nearest distances. To predict the class label of a virus, we first find its nearest neighbor which belongs to class I, for example, then compare its nearest distance with the .75th quantile of the nearest distances of class I. We make the prediction only if the nearest distance is less than the .75th quantile, called a prediction with .75-cutoff. Based on this cut-off setting, for classes I and VII, the inconsistency rate for circular viruses is updated to 2/(177+44) = 2/221 = 0.90%. For classes IV, V, and VI, the inconsistency rate for linear viruses is updated to 16/(563+66+58) = 16/687 = 2.33%. Thus the overall inconsistency rate is 18/(599+221+687+1) = 1.19%. Therefore, in this study we calculate a .75 cut-off for predicting Baltimore class, family, subfamily, and genus labels to avoid unreliable predictions. For cut-off quantile other than .75, see Tables S4–S5 in File SI.

Secondly, given a virus with only sequence information, we can predict its Baltimore class label with exceptional results. As shown in Table 1, of the 2,044 viruses only 4+14+5+21+2+7+1 = 54 are labelled by the nearest neighbor predictor with the incorrect Baltimore class; an inconsistency rate of 2.64%. The inconsistency rates for classes III (0.11) and VI (0.12) are much higher than other classes due to their smaller class sizes (45 for III and 58 for VI). With more virus samples added into the database, the inconsistency rate could be reduced further. For any virus missing Baltimore class information we check its nearest-neighbor's label. If the distance is sufficiently small we say with confidence that the missing label is the same as that of its nearest-neighbor. Using this method we obtain the Baltimore class prediction results presented in Table S6 of File SI.

### Predict family, subfamily, and genus label

We next go one level deeper and check the predictability of family labels given their Baltimore classification with the NV using the same nearest neighbor prediction framework. Given a virus with only sequence information, we can predict its family label according to Baltimore class as shown in Table 1. Of the 2,044 viruses, only 58+11 = 69 are assigned inconsistent family labels relative to their nearest neighbors; an inconsistency rate of 3.38%. We can again perform the same process with subfamily and genus labels given the family information. Those results are presented in Table S7 of File SI. Of the 2,044 viruses, only 6 are assigned inconsistent subfamily labels relative to their nearest neighbors; an inconsistency rate of 0.29%. Similarly, only 57 viruses are assigned inconsistent genus labels; an inconsistency rate of 2.79%.

Using our method we are also able to make predictions for viruses with no assigned family, subfamily, or genus labels. For any virus missing any of the above information we check its nearest-neighbor's label. If the distance is sufficiently small we say with confidence that the missing label is the same as that of its nearest-neighbor. Using this method we obtain the results presented in Tables S8–S10 in File SI.

### Natural graphical representation of viral phylogeny

**Figure 2:**
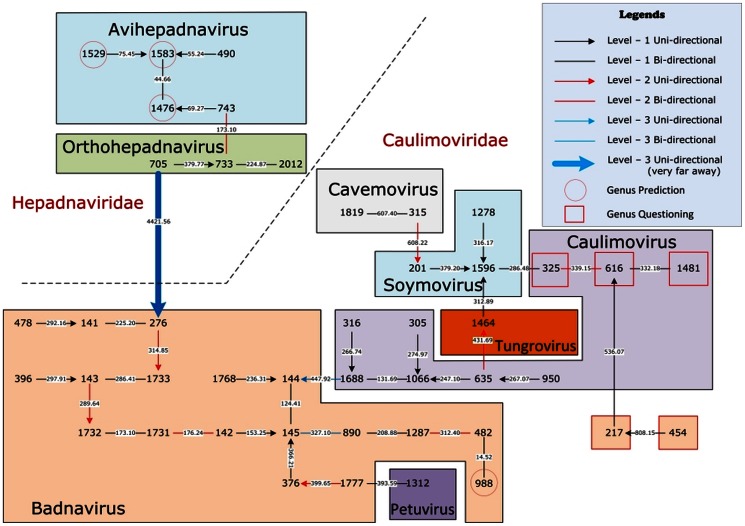
The natural graphical representation for the 44 single-segment referenced viruses of Baltimore VII.

In Figure 2, we give the natural graphical representation for the 44 single-segment referenced viruses of Baltimore VII. Each integer represents a virus (see Supporting Information) and each real number on an arrow is the distance between the two viruses. The two families *Hepadnaviridae* and *Caulimoviridae* are clearly separated in the graph. In the *Hepadnaviridae* family there are two genera *Avihepadnavirus* and *Orthohepadnavirus*. Viruses #1476 (Ross's goose hepatitis B), #1529 (Sheldgoose hepatitis B), and #1583 (Snow goose hepatitis B) are not assigned ICTV genus labels. In the figure we can see their nearest neighbors are all in *Avihepadnavirus* genus, thus we predict that they belong to the genus *Avihepadnavirus*. These predictions are consistent with other researchers' work [18]–[20]. There are six genera (*Badnavirus*, *Petuvirus*, *Caulimovirus*, *Cavemovirus*, *Soymovirus*, and *Tungrovirus*) in the family *Caulimoviridae*. Virus #988 (Lucky bamboo bacilliform) has no genus label and its nearest neighbor is virus #482 (Dracaena mottle). The distance between the two viruses is only 14.52, far less than the other distances, thus we predict confidently virus #988 is also in the *Badnavirus* genus. This prediction is consistent with the result obtained by Chen et al. [21]. Virus #217 (Bougainvillea spectabilis chlorotic vein-banding) and virus #454 (Cycad leaf necrosis) are labelled as *Badnaviruses* by ICTV. However, in our genome space these two viruses are far away from all the other *Badnaviruses*. Similarly, viruses #325 (Cestrum yellow leaf curling), #616 (Eupatorium vein clearing), and #1481 (Rudbeckia flower distortion) are far away from all the other *Caulimoviruses*. Thus we question the ICTV genus classifications for these viruses as shown in the figure.

**Figure 3:**
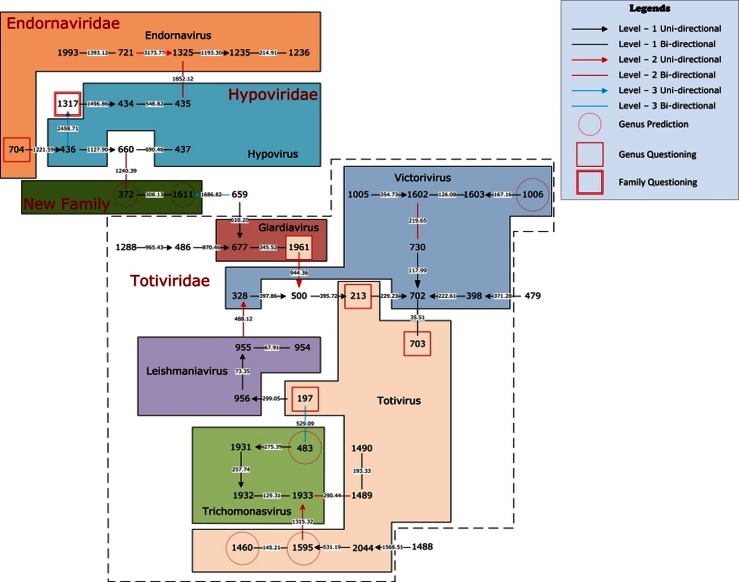
The natural graphical representation for the 45 single-segment referenced viruses of Baltimore III.

In Figure 3, we also give the natural graphical representation for the 45 single-segment referenced viruses of Baltimore III. The three families *Endornaviridae*, *Hypoviridae*, and *Totiviridae* are clearly separated in this graph. Virus #372 (Circulifer tenellus virus 1) and #1611 (Spissistilus festinus virus 1) are not assigned with ICTV family labels. They are very close together (distance 308.13), but far away from the other three families. Thus we predict these two viruses belong to a new family. This prediction is consistent with Spear et al.'s work [22]. ICTV puts virus #1317 (Phlebiopsis gigantean mycovirus dsRNA 1) into *Totiviridae* family, however, in our 12-dimensional genome space it is far away from the majority of *Totiviridae* family. Thus we question the family classification for this virus. Similarly, Lim et al. [23] studied the complete genome sequence of this virus and agree that it may be from a novel family. Viruses #483 (Drosophila A), #1006 (Magnaporthe oryzae virus 2), #1460 (Rhododendron virus A), and #1595 (Southern tomato) are not assigned ICTV genus labels. Our predictions are consistent with the work of other researchers [24]–[27]. Virus #704 (Gremmeniella abietina type B RNA virus XL1) is put into *Endornaviridae* family and *Endornavirus* genus by [28]. However, the authors make this decision by analyzing the alignments of only conserved gene regions of viruses. On the other hand, our NV is constructed using the entire genome sequence leading us to question this classification result. Similarly, we question the genus labels of viruses #197 (Black raspberry virus F), #213 (Botryotinia fuckeliana totivirus 1), #703 (Gremmeniella abietina RNA virus L2), and #1961 (Ustilago maydis virus H1) in Totivirus. It should be noted that the *Totiviridae* family includes not only single-segment but also multi-segment genomes (Helicobasidium mompa No. 17 dsRNA virus). Here we only focus on our dataset of single-segment viruses. Further study of multi-segment viruses may provide an explanation for the scattering of the *Totiviridae* family members.

**Figure 4:**
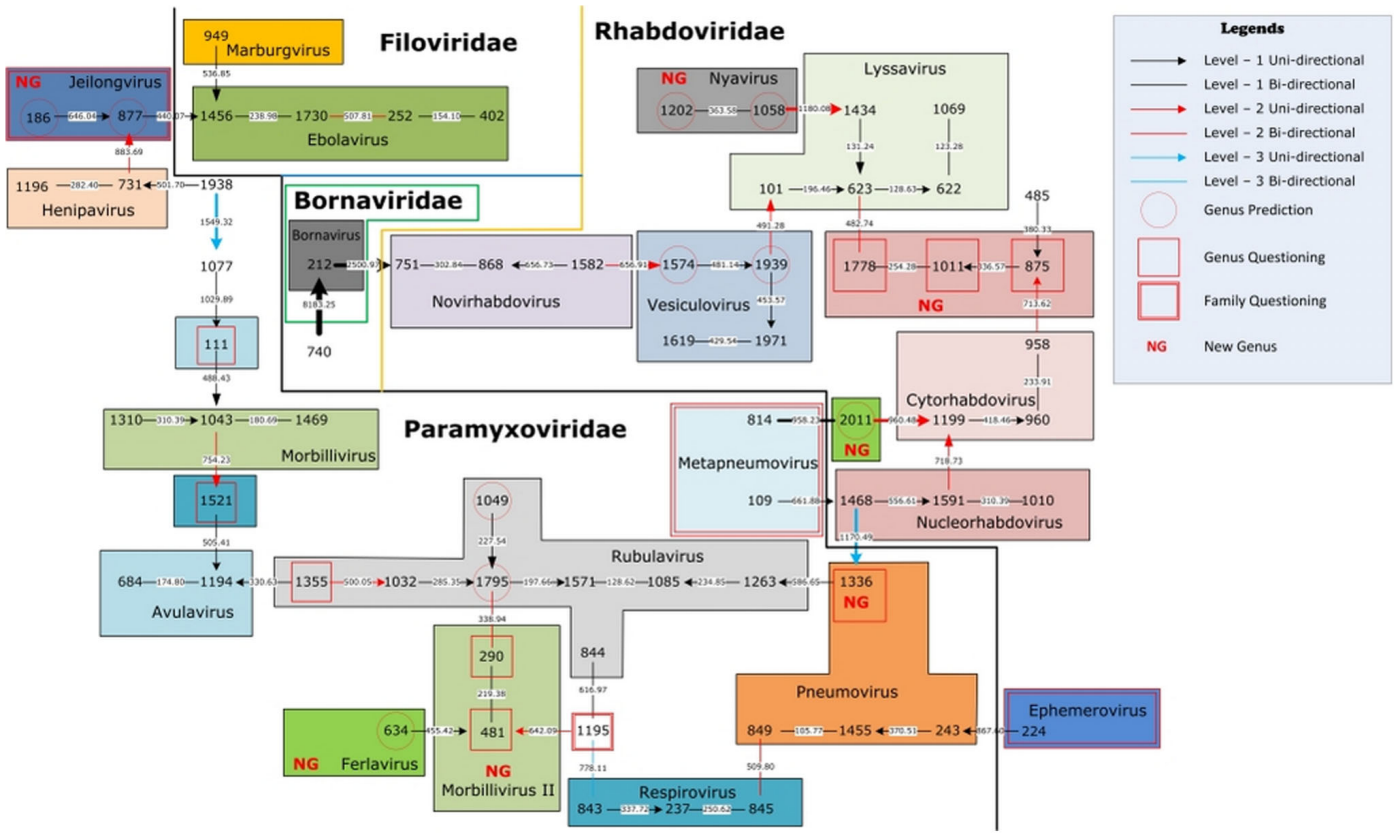
The natural graphical representation for the 67 single-segment referenced viruses of Baltimore V.

In Figure 4, we give the natural graphical representation for the 67 single-segment referenced viruses of Baltimore V. The four families *Filoviridae*, *Rhabdoviridae*, *Bornaviridae*, and *Paramyxoviridae* are clearly separated in this graph. We predict that viruses #186 (Beilong virus) and #877 (J virus) form a new genus since these two close viruses are far away from other genera in *Paramyxoviridae* family. Actually, this prediction is precisely consistent with other researchers' work [29], [30], and these authors have named this new genus as *Jeilongvirus*. Viruse #634 (Fer de lance virus) also belongs to a new genus according to our work, and other researchers [31] have named this new genus as *Ferlavirus*. Viruses #1202 (Nyamanini virus) and #1058 (Midway virus) form a new genus named *Nyavirus* according to the literature [32]. This is consistent with our prediction. Similarly, according to our graphical representation we also suggest several new genera. Viruses #290 (Canine distemper virus) and #481 (Dolphin morbillivirus) form a new genus, we name it as *Morbillivirus* II; viruses #875 (Iranian maize mosaic nucleorhabdovirus), #1011 (Maize mosaic virus), and #1778 (Taro vein chlorosis virus) form a new genus; virus #2011 (Wongabel virus) form a new genus, which is consistent with Gubala et al.'s work [33]; virus #1336 (Pneumonia virus of mice J3666) form a new genus. We also question genus classifications for several viruses, including viruses #111 (Avian paramyxovirus 6), #1521 (Sendai virus), #1355 (Porcine rubulavirus). The authors [34]–[36] make these classification decisions by analyzing specific gene coding regions or protein sequences, while our natural vector uses the global sequence information of genomes. Furthermore, for genera *Ephemerovirus* and *Jeilongvirus*, we question their family classifications provided by ICTV because they are closer to other families (*Jeilongvirus* to family *Filoviridae* and *Ephemerovirus* to family *Paramyxoviridae*) based on our graphical representation. For two *Metapneumovirus* memebers #109 (Avian metapneumovirus) and #814 (Human metapneumovirus), ICTV puts them into the *Paramyxoviridae* family, while our results show that they are closer to the *Rhabdoviridae* family. In addition, these two viruses are not connected in our graphical representation. One possible reason is that there might be some metapneumovirus viruses from animals other than human and avian that may be missing from our dataset.

For Baltimore classes I (776 viruses), II (328 viruses), IV (563 viruses), and VI (58 viruses), we provide complete nearest-neighbor relationships in the graph description of Baltimore classes at Supporting Information. It is computationally difficult, if not infeasible, for multiple sequence alignment to handle these large classes.

**Figure 5:**
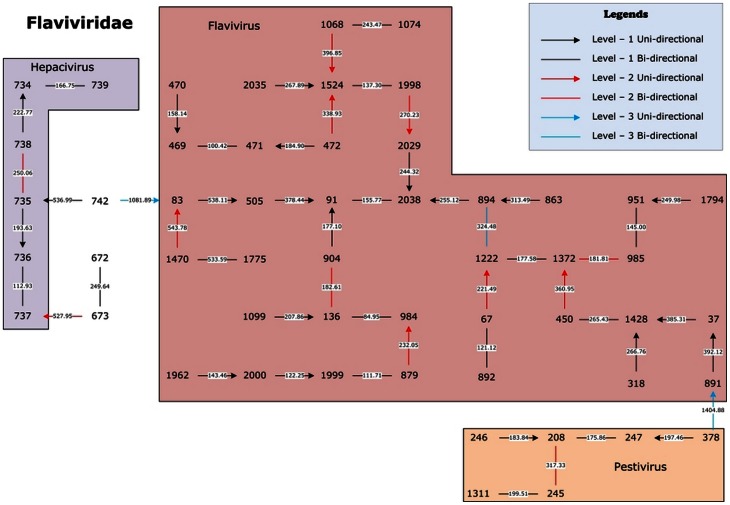
The natural graphical representation for 53 viruses of the *Flaviviridae* family.

West Nile virus (WNv) is a single-stranded plus-sense RNA virus that is classified within the family *Flaviviridae* and genus *Flavivirus* by the ICTV. Recent reports of widespread transmission of this mosquito-borne virus in humans in the United States highlight this threat to public health. During August 2012, the Centers for Disease Control and Prevention reported 1,590 human cases and 65 deaths nationwide [37]. To study the origin of this virus we use our method to visualize 53 viruses of *Flaviviridae* family in our dataset. In Figure 5, the three genera *Hepacivirus*, *Flavivirus*, and *Pestivirus* are clearly separated. Viruses #1999 (NC_001563.2) and #2000 (NC_009942.1) are two West Nile viruses, and viruses #879 (Japanese encephalitis virus, NC_001437.1) and #1962 (Usutu virus, NC_006551.1) are closest to them, respectively. This is consistent with [38] that both WNv and Usutu virus belong to the Japanese encephalitis antigenic complex. Thus, the phylogenetic relationship of *Flaviviridae* family revealed in our findings could provide more information helpful to public health officials for prevention and treatment.

## Discussion

As a comparison, we also do Multiple Sequence Alignment (MSA) analysis for three small Baltimore classes (III, dsRNA; V, ssRNA(−); VII, dsDNA(RT)) to test if each virus' nearest neighbor belongs to the same family as itself. We use the ClustalW program from the MEGA 5.0 software [39] to do the alignment for these three groups and then check the label consistency of the viruses with its nearest neighbor. There are no inconsistent family labels found in Baltimore classes V and VII, and 1 inconsistent family label in Baltimore class III. The inconsistency might be caused by MSA's inability to differentiate between families of viruses with different mutation rates (3). However, one of the most serious problems for MSA is the computational time. MSA requires approximately 2 hours, 10 hours, and 1 hour to generate the alignment results for Baltimore classes III, V and VII, respectively, on a PC computer (CPU 1.67 GHz, 3 GB of RAM). Using our method, it takes 2.1 seconds, 9.4 seconds, and 1.7 seconds respectively to get similar results (see Table 1) on the same computer. For Baltimore classes I (776 viruses), II (328 viruses), and IV (563 viruses) it is computationally difficult, if not infeasible, for MSA to handle these large classes. Using our method, it takes about 76.7 minutes, 5.2 seconds, and 45.1 seconds, respectively. Note that most of the computational time is spent calculating the natural vectors. To classify a new virus, typically it takes less than one second to calculate its natural vector and determine its classifications. Our approach outperforms MSA with respect to computational efficiency since there is no need to recalculate the natural vectors again for the known viruses. As for Baltimore class VI, there is no need for a check because all the 58 viruses belong to one family.

In conclusion, there are four major advantages to our method: (1) Once a virus' NV has been calculated, it can be stored in a database. It is unnecessary to recalculate the NV of a virus for any subsequent applications, whereas in multiple alignment methods realignment is necessary when additional sequences are added to the previously aligned group. (2) Our method is much faster than alignment methods and easier to manipulate. The complexity of our method is 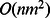 with length n and number m of viral genome sequences which is much faster in providing accurate comparisons than other known methods such as multiple sequence alignment with 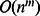. (3) One can have a global comparison of all viral genomes simultaneously. Using our new two-dimensional phylogenetic graph, the results can be displayed and viewed clearly; this is user-friendly and allows even non-experts to understand the relationship among different genomes via the graph of the genome space. (4) The NV method is robust with respect to deletion, duplication, and inversion. Typically, the change of natural vector measured in Euclidean distance is around *k* with respect to mutations involving *k* letters (*k* is negligible with respect to the length *n*). See Section 4 Simulated evaluation of (12-dimensional) genome space including Table S11 in File SI for more details.

## Supporting Information

File SI **This file SI contains: (1) Dataset, including Table S1–S3; (2) Discussion on cut-off setting, including Table S4–S5; (3) Predictions by our method, including Table S6–S10; (4) Simulated evaluation of (12-dimensional) genome space, including Table S11; (5) Graph descriptions of Baltimore I, II, IV, VI; (6) List of virus information used in this paper; (7) Supplementary references.**
(PDF)
