# Supplementary material for: Real Time Classification of Viruses in 12 Dimensions
Source: PLoS One. 2013 May 22;8(5):e64328. doi: 10.1371/journal.pone.0064328 (PMC3661469; doi:10.1371/journal.pone.0064328)
Supplement: File SI — This file SI contains: (1) Dataset, including Table S1–S3; (2) Discussion on cut-off setting, including Table S4–S5; (3) Predictions by our method, including Table S6–S10; (4) Simulated evaluation of (12-dimensional) genome space, including Table S11; (5) Graph descriptions of Baltimore I, II, IV, VI; (6) List of virus information used in this paper; (7) Supplementary references. (PDF) [file pone.0064328.s001.pdf]

# Supporting Information for

## Real Time Classification of Viruses in 12 Dimensions

Chenglong Yu, Troy Hernandez, Hui Zheng, Shek-Chung Yau, Hsin-Hsiung Huang, Rong Lucy He,

Jie Yang, Stephen S.-T. Yau*

*To whom correspondence should be addressed. Email: yau@uic.edu

**This PDF file includes:**


| | |
|---|---|
| (1) Dataset, including Table S1-S3 | Pages 1 - 3 |
| (2) Discussion on cut-off setting, including Table S4-S5 | Page 4 |
| (3) Predictions by our method, including Table S6-S10 | Pages 5 - 9 |
| (4) Simulated evaluation of (12-dimensional) genome space, including Table S11 | Pages 10 - 16 |
| (5) Graph descriptions of Baltimore I, II, IV, VI | Pages 17 - 72 |
| (6) List of virus information | Pages 73 - 114 |
| (7) Supplementary references. | Page 114 |


## 1. Dataset

The composition and structure of viral genomes is more varied than bacterial, plant, or animal kingdoms. The viral genomes may be single-stranded or double-stranded, linear or circular, and in single-segmented or multi-segmented configuration. There are 2,418 viral genomes in the current GenBank collection of reference sequences (up to 2012-4-6). Among the 2,418 reference records, there are 2,044 single-segment viruses that we focus on in this study. We leave the remaining 374 multi-segment viruses for future study.

Baltimore classification is a classification system that places viruses into one of seven groups depending on a combination of their nucleic acid (DNA or RNA), stranded-ness (single-stranded or double-stranded), sense (+ or -), and method of replication (4). In **Supplementary Table 1**, we show the original Baltimore classification information of the 2,044 single-segmented referenced viruses.

The classification information is originally based on the NCBI annotations of these viruses. Satellites have no Baltimore classes and <NA> refers to unknown classifications.

The International Committee on Taxonomy of Viruses (ICTV) also develops a universal taxonomic scheme for viruses and aims to describe all of the viruses of living organisms by assigning them taxa, order, family, subfamily, genus, and species (5). All viruses belonging to the same family should have the same Baltimore classification. After checking the consistency between Baltimore classification and ICTV families, we find that the original records of the viruses in the *Retroviridae* family contain erroneous DNA label information that we correct when determining the Baltimore classification labels. Additionally, there are 17 families having both circular virus(es) and linear virus(es) (see **Supplementary Table 2**). This violates the ICTV classification criteria (5). We use within-family majority voting to determine the correct shape labels. Therefore we make corrections to the shape information of these viruses. After corrections, among the 2,044 single-segmented viruses, there are 605 circular viruses and 1,439 linear viruses. The updated summary of the Baltimore classification information is shown in **Table 1** of the main text. Satellites have no Baltimore class labels and <NA> refers to unknown classifications. (Note: The Deltavirus was assigned to group V and an unassigned ssRNA virus (NC_007518.1) was assigned to group IV based on the literature (40, 41)). The list of all 2,044 virus' information is at the end of this document.

According to ICTV classification, of the 2,044 viruses, there are 248 viruses without family labels. The remaining 1,796 viruses belong to 72 families. The missing label rate is 12.1%. There are 1,732 viruses without subfamily labels. The remaining 312 viruses belong to 17 subfamilies. The missing label rate is 84.7%. Only 9 families have subfamilies. There are 604 viruses without genus labels. The remaining 1,440 viruses belong to 258 genera. The missing label rate is 30.0%. Due to the dramatically increased rate of virus sequencing, the expert time and technical resources of the ICTV are too restricted to provide detailed classification information for all viruses.

**Supplementary Table 1**: The original Baltimore classification information of the 2,044 single-segmented referenced viruses based on the NCBI annotations of these viruses.

| Class | I | II | III | IV | V | VI | VII | Satellite | <NA> |
|---|---|---|---|---|---|---|---|---|---|
| Name | dsDNA | ssDNA | dsRNA | ssRNA (+) | ssRNA (-) | ssRNA (RT) | dsDNA (RT) | | |
| Linear | 558 | 65 | 45 | 562 | 67 | 38 | 30 | 33 | 20 |
| Circular | 218 | 263 | 0 | 0 | 1 | 0 | 33 | 103 | 8 |
| Total | 776 | 328 | 45 | 562 | 68 | 38 | 63 | 136 | 28 |

**Supplementary Table 2**: 17 families containing different shape labels based on NCBI annotations.

| Family | circular | linear |
|---|---|---|
| *Anelloviridae* | 35 | 1 |
| *Ascoviridae* | 3 | 1 |
| *Baculoviridae* | 52 | 1 |
| *Caulimoviridae* | 26 | 10 |
| *Geminiviridae* | 160 | 7 |
| *Hepadnaviridae* | 7 | 1 |
| *Herpesviridae* | 5 | 38 |
| *Inoviridae* | 24 | 3 |
| *Iridoviridae* | 1 | 8 |
| *Myoviridae* | 18 | 85 |
| *Papillomaviridae* | 52 | 17 |
| *Parvoviridae* | 2 | 54 |
| *Phycodnaviridae* | 2 | 8 |
| *Podoviridae* | 13 | 75 |
| *Polyomaviridae* | 21 | 2 |
| *Rudiviridae* | 1 | 3 |
| *Siphoviridae* | 22 | 229 |

**Supplementary Table 3**: IUPAC nucleotide code table.

| IUPAC Code | Nucleotide Base |
|---|---|
| A | Adenine |
| C | Cytosine |
| G | Guanine |
| T (or U) | Thymine (or Uracil) |
| R | A or G |
| Y | C or T |
| S | G or C |
| W | A or T |
| K | G or T |
| M | A or C |
| B | C or G or T |
| D | A or G or T |
| H | A or C or T |
| V | A or C or G |
| N | A or C or G or T |

## 2. Discussion on cut-off setting

In Section Result of the paper, we use a 75% cut-off to avoid unreliable predictions based on distant neighbors. **Supplementary Table 4 and 5** below show prediction results with options other than 75%. Based on the case “IV, V, VI linear” (to classify all linear viruses in Baltimore classes IV, V, VI) in the two tables, the number of errors do not increase much as the cut-off point increases from 0.5, 0.6, or 0.7 to 0.75 (the corresponding numbers of errors are 7, 10, 15, and 16). After the 0.75 cut-off point, the increase in the number of errors becomes significant (the corresponding numbers of errors are 22, 36, and 43). To keep a reasonably good accuracy rate for prediction without removing too many predictions due to distant neighbors, we suggest 75% as a cut-off point.

**Supplementary Table 4**: Number of errors for different cut-off points.

| Case | 0.50 | 0.60 | 0.70 | 0.75 | 0.80 | 0.90 | 0.95 | 0.99 | no cut-off |
|---|---|---|---|---|---|---|---|---|---|
| I&VII, linear | 0 | 0 | 0 | 0 | 0 | 0 | 0 | 0 | 0 |
| I&VII, circular | 0 | 2 | 2 | 2 | 2 | 2 | 2 | 2 | 3 |
| IV,V,VI, linear | 7 | 10 | 15 | 16 | 22 | 36 | 43 | 44 | 45 |
| IV,V,VI, circular | 0 | 0 | 0 | 0 | 0 | 0 | 0 | 0 | 0 |

**Supplementary Table 5**: Error rates (%) for different cut-off points

| Case | 0.50 | 0.60 | 0.70 | 0.75 | 0.80 | 0.90 | 0.95 | 0.99 | no cut-off | Total number of Viruses |
|---|---|---|---|---|---|---|---|---|---|---|
| I&VII, linear | 0 | 0 | 0 | 0 | 0 | 0 | 0 | 0 | 0 | 599 |
| I&VII, circular | 0 | 0.9 | 0.9 | 0.9 | 0.9 | 0.9 | 0.9 | 0.9 | 1.36 | 221 |
| IV,V,VI, linear | 1.02 | 1.46 | 2.18 | 2.33 | 3.20 | 5.24 | 6.26 | 6.40 | 6.55 | 687 |
| IV,V,VI, circular | 0 | 0 | 0 | 0 | 0 | 0 | 0 | 0 | 0 | 1 |

## 3. Predictions by our method

Using our method we are also able to make predictions for viruses with no assigned Baltimore class or family, subfamily, or genus labels. For any virus missing any of the above information we check its nearest-neighbor's label and confirm that the distance is below the cutoff for that class. If the distance is sufficiently small we say with confidence that the missing label is the same as that of its nearest-neighbor. Using this method we obtain the results presented in **Supplementary Table 6, 8-10**. The overall prediction accuracy rate is 96.1% for Baltimore class labels, 94.3% for family labels, 97.1% for subfamily labels, and 92.9% for genus labels. **Supplementary Table 7** is the genus and subfamily classification prediction information given Family information.

**Supplementary Table 6**: Baltimore class label predictions of 19 viruses by our new method.

| Accession No. | Virus | Baltimore class prediction |
|---|---|---|
| NC_004821.1 | Bacillus phage phBC6A52 | I |
| NC_007193.2 | Chaetoceros salsugineum DNA virus | IV |
| NC_014473.1 | Croton yellow vein virus | II |
| NC_009552.2 | Geobacillus virus E2 | I |
| NC_014321.1 | Hyperthermophilic Archaeal Virus 2 | I |
| NC_005964.1 | Mycoplasma phage phiMFV1 | I |
| NC_010392.1 | Phage Gifsy-1 | I |
| NC_010393.1 | Phage Gifsy-2 | I |
| NC_010391.1 | Salmonella phage Fels-1 | I |
| NC_007902.1 | Sodalis phage phiSG1 | I |
| NC_011132.1 | Sputnik virophage | I |
| NC_009762.2 | Staphylococcus phage tp310-2 | I |
| NC_009763.2 | Staphylococcus phage tp310-3 | I |
| NC_004584.1 | Streptococcus pyogenes phage 315.1 | I |
| NC_004585.1 | Streptococcus pyogenes phage 315.2 | I |
| NC_004586.1 | Streptococcus pyogenes phage 315.3 | I |
| NC_004587.1 | Streptococcus pyogenes phage 315.4 | I |
| NC_004588.1 | Streptococcus pyogenes phage 315.5 | I |
| NC_004589.1 | Streptococcus pyogenes phage 315.6 | I |

**Supplementary Table 7**: Genus and Subfamily classification prediction information given Family information.

| Family names | Count | Subfamily Inconsistencies | Inconsistency Rate | Genus Inconsistencies | Inconsistency Rate |
|---|---|---|---|---|---|
| *Adenoviridae* | 26 | 0 | 0 | 1 | 0.038 |
| *Alloherpesviridae* | 5 | 0 | 0 | 0 | 0 |
| *Alphaflexiviridae* | 40 | 0 | 0 | 1 | 0.025 |
| *Alvernaviridae* | 1 | 0 | 0 | 0 | 0 |
| *Ampullaviridae* | 1 | 0 | 0 | 0 | 0 |
| *Anelloviridae* | 36 | 0 | 0 | 0 | 0 |
| *Arteriviridae* | 4 | 0 | 0 | 0 | 0 |
| *Ascoviridae* | 4 | 0 | 0 | 0 | 0 |
| *Asfarviridae* | 1 | 0 | 0 | 0 | 0 |
| *Astroviridae* | 11 | 0 | 0 | 2 | 0.181 |
| *Bacillariornaviridae* | 1 | 0 | 0 | 0 | 0 |
| *Baculoviridae* | 53 | 0 | 0 | 3 | 0.057 |
| *Barnaviridae* | 1 | 0 | 0 | 0 | 0 |
| *Betaflexiviridae* | 46 | 0 | 0 | 0 | 0 |
| *Bicaudaviridae* | 2 | 0 | 0 | 0 | 0 |
| *Bornaviridae* | 1 | 0 | 0 | 0 | 0 |
| *Caliciviridae* | 19 | 0 | 0 | 2 | 0.105 |
| *Caulimoviridae* | 36 | 0 | 0 | 2 | 0.056 |
| *Circoviridae* | 14 | 0 | 0 | 0 | 0 |
| *Closteroviridae* | 14 | 0 | 0 | 1 | 0.071 |
| *Coronaviridae* | 31 | 1 | 0.032258 | 4 | 0.129 |
| *Corticoviridae* | 1 | 0 | 0 | 0 | 0 |
| *Dicistroviridae* | 14 | 0 | 0 | 0 | 0 |
| *Endornaviridae* | 6 | 0 | 0 | 0 | 0 |
| *Filoviridae* | 5 | 0 | 0 | 0 | 0 |
| *Flaviviridae* | 53 | 0 | 0 | 0 | 0 |
| *Flexiviridae* | 1 | 0 | 0 | 0 | 0 |
| *Fuselloviridae* | 9 | 0 | 0 | 0 | 0 |
| *Gammaflexiviridae* | 1 | 0 | 0 | 0 | 0 |
| *Geminiviridae* | 167 | 0 | 0 | 0 | 0 |
| *Globuloviridae* | 2 | 0 | 0 | 0 | 0 |
| *Hepadnaviridae* | 8 | 0 | 0 | 0 | 0 |
| *Hepeviridae* | 1 | 0 | 0 | 0 | 0 |
| *Herpesviridae* | 43 | 3 | 0.069767 | 9 | 0.209 |
| *Hypoviridae* | 4 | 0 | 0 | 0 | 0 |
| *Iflaviridae* | 8 | 0 | 0 | 0 | 0 |
| *Inoviridae* | 27 | 0 | 0 | 0 | 0 |
| *Iridoviridae* | 9 | 0 | 0 | 1 | 0.111 |
| *Labyrnaviridae* | 1 | 0 | 0 | 0 | 0 |

| | | | | | |
|---|---|---|---|---|---|
| *Leviviridae* | 7 | 0 | 0 | 0 | 0 |
| *Lipothrixviridae* | 8 | 0 | 0 | 0 | 0 |
| *Luteoviridae* | 21 | 0 | 0 | 2 | 0.095 |
| *Malacoherpesviridae* | 1 | 0 | 0 | 0 | 0 |
| *Marnaviridae* | 1 | 0 | 0 | 0 | 0 |
| *Microviridae* | 14 | 0 | 0 | 0 | 0 |
| *Myoviridae* | 103 | 0 | 0 | 0 | 0 |
| *Nanoviridae* | 1 | 0 | 0 | 0 | 0 |
| *Narnaviridae* | 10 | 0 | 0 | 0 | 0 |
| *Nimaviridae* | 1 | 0 | 0 | 0 | 0 |
| *Papillomaviridae* | 69 | 0 | 0 | 8 | 0.116 |
| *Paramyxoviridae* | 33 | 0 | 0 | 1 | 0.030 |
| *Parvoviridae* | 56 | 2 | 0.035714 | 1 | 0.018 |
| *Phycodnaviridae* | 10 | 0 | 0 | 0 | 0 |
| *Picornaviridae* | 58 | 0 | 0 | 3 | 0.052 |
| *Plasmaviridae* | 1 | 0 | 0 | 0 | 0 |
| *Podoviridae* | 88 | 0 | 0 | 1 | 0.011 |
| *Polyomaviridae* | 23 | 0 | 0 | 0 | 0 |
| *Potyviridae* | 79 | 0 | 0 | 3 | 0.039 |
| *Poxviridae* | 27 | 0 | 0 | 1 | 0.037 |
| *Retroviridae* | 58 | 0 | 0 | 5 | 0.086 |
| *Rhabdoviridae* | 25 | 0 | 0 | 0 | 0 |
| *Roniviridae* | 1 | 0 | 0 | 0 | 0 |
| *Rudiviridae* | 4 | 0 | 0 | 0 | 0 |
| *Secoviridae* | 3 | 0 | 0 | 0 | 0 |
| *Siphoviridae* | 251 | 0 | 0 | 1 | 0.004 |
| *Tectiviridae* | 4 | 0 | 0 | 0 | 0 |
| *Tetraviridae* | 3 | 0 | 0 | 0 | 0 |
| *Togaviridae* | 17 | 0 | 0 | 0 | 0 |
| *Tombusviridae* | 40 | 0 | 0 | 2 | 0.05 |
| *Totiviridae* | 26 | 0 | 0 | 3 | 0.115 |
| *Tymoviridae* | 22 | 0 | 0 | 0 | 0 |
| *Virgaviridae* | 24 | 0 | 0 | 0 | 0 |

**Supplementary Table 8**:  Family label predictions of 21 viruses by our new method.

| Accession No. | Virus | Family prediction |
|---|---|---|
| NC_010356.1 | Glossina pallidipes salivary gland hypertrophy virus | *Iridoviridae* |
| NC_014322.1 | Hyperthermophilic Archaeal Virus 1 | *Globuloviridae* |
| NC_013756.1 | Marseillevirus | *Phycodnaviridae* |
| NC_010671.1 | Musca domestica salivary gland hypertrophy virus | *Baculoviridae* |

| | | |
|---|---|---|
| NC_011588.1 | Oryctes rhinoceros virus | *Baculoviridae* |
| NC_007808.1 | Pseudomonas phage PA11 | *Siphoviridae* |
| NC_009597.1 | Pyrococcus abyssi virus 1 | *Podoviridae* |
| NC_004313.1 | Salmonella phage ST64B | *Siphoviridae* |
| NC_003324.1 | Sinorhizobium phage PBC5 | *Siphoviridae* |
| NC_013195.1 | Staphylococcus phage P954 | *Siphoviridae* |
| NC_012784.1 | Staphylococcus phage phiPVL-CN125 | *Siphoviridae* |
| NC_007045.1 | Staphylococcus phage PT1028 | *Siphoviridae* |
| NC_007058.1 | Staphylococcus phage ROSA | *Siphoviridae* |
| NC_014099.1 | Sulfolobus turreted icosahedral virus 2 | *Rudiviridae* |
| NC_007710.1 | Xanthomonas phage OP2 | *Myoviridae* |
| NC_013028.1 | Circovirus-like genome CB-A | *Anelloviridae* |
| NC_012958.1 | Drosophila A virus | *Totiviridae* |
| NC_013469.1 | Fusarium graminearum dsRNA mycovirus-3 | *Hypoviridae* |
| NC_009757.1 | Marine RNA virus JP-A | *Dicistroviridae* |
| NC_009758.1 | Marine RNA virus JP-B | *Dicistroviridae* |
| NC_011187.1 | Rubus chlorotic mottle virus | *Tombusviridae* |

**Supplementary Table 9**: Subfamily label predictions of 4 viruses by our new method.

| Accession No. | Virus | Subfamily prediction |
|---|---|---|
| NC_004775.1 | Salmonella phage epsilon15 | *Autographivirinae* |
| NC_007804.2 | Escherichia phage phiV10 | *Autographivirinae* |
| NC_005344.1 | Shigella phage Sf6 | *Autographivirinae* |
| NC_010363.1 | Lactococcus phage asccphi28 | *Picovirinae* |

**Supplementary Table 10**: Genus label predictions of 37 viruses by our new method.

| Accession No. | Virus | Genus prediction |
|---|---|---|
| NC_013060.1 | Astrovirus VA1 | *Mamastrovirus* |
| NC_011062.1 | Potato virus T | *Capillovirus* |
| NC_006875.1 | Calicivirus isolate TCG | *Nebovirus* |
| NC_004541.1 | Walrus calicivirus | *Vesivirus* |
| NC_009568.1 | Lucky bamboo bacilliform virus | *Badnavirus* |
| NC_001836.1 | Little cherry virus 1 | *Closterovirus* |
| NC_014324.1 | Turnip curly top virus | *Curtovirus* |
| NC_012757.1 | Vibrio phage VEJphi | *Inovirus* |
| NC_008294.1 | Pseudomonas phage PRR1 | *Levivirus* |
| NC_009760.1 | Bacillus phage 0305phi8-36 | *phiKZ-like viruses* |

| | | |
|---|---|---|
| NC_012530.1 | Lactobacillus phage Lb338-1 | *SPO1-like viruses* |
| NC_010811.2 | Ralstonia phage RSL1 | *phiKZ-like viruses* |
| NC_004735.1 | Rhodothermus phage RM378 | *SPO1-like viruses* |
| NC_014143.1 | Bettongia penicillata papillomavirus 1 | *Deltapapillomavirus* |
| NC_004074.1 | Tioman virus | *Rubulavirus* |
| NC_004290.1 | Acheta domestica densovirus | *Densovirus* |
| NC_012685.1 | Culex pipiens densovirus | *Densovirus* |
| NC_007218.1 | Penaeus merguiensis densovirus | *Parvovirus* |
| NC_004775.1 | Salmonella phage epsilon15 | *T7-like viruses* |
| NC_007804.2 | Escherichia phage phiV10 | *T7-like viruses* |
| NC_010363.1 | Lactococcus phage asccphi28 | *AHJD-like viruses* |
| NC_001825.1 | Streptococcus phage Cp-1 | *Phi29-like viruses* |
| NC_014037.1 | Sugarcane streak mosaic virus | *Potyvirus* |
| NC_008030.1 | Crocodilepox virus | *Molluscipoxvirus* |
| NC_001993.1 | Melanoplus sanguinipes entomopoxvirus | *Betaentomopoxvirus* |
| NC_005947.1 | Avian endogenous retrovirus EAV-HP | *Alpharetrovirus* |
| NC_007020.1 | Tupaia virus | *Dimarhabdovirus supergroup* |
| NC_010583.1 | Enterobacteria phage EPS7 | *T5-like viruses* |
| NC_006949.1 | Enterobacteria phage ES18 | *N15-like viruses* |
| NC_004112.1 | Lactobacillus phage A2 | *Lambda-like viruses* |
| NC_008203.1 | Mycobacterium phage Che12 | *L5-like viruses* |
| NC_005178.1 | Pseudomonas phage D3112 | *Lambda-like viruses* |
| NC_007805.1 | Pseudomonas phage F10 | *Lambda-like viruses* |
| NC_011613.1 | Pseudomonas phage MP29 | *Lambda-like viruses* |
| NC_005069.1 | Yersinia phage PY54 | *T1-like viruses* |
| NC_010246.1 | Magnaporthe oryzae virus 2 | *Victorivirus* |
| NC_012484.1 | Grapevine Syrah Virus-1 | *Marafivirus* |

## 4. Simulated evaluation of (12-dimensional) genome space

We choose 2 real viral genome sequences:

(1) HIV-1 E9 from the USA, complete genome (AF070521, GI: 3378121). Length: 9699.

(2) HIV-2 isolate MCN13, complete genome (AY509259, GI: 41056775). Length: 9713.

The 12-dimensional natural vectors for these two:

| | na | ua | Da | ng | ug | Dg | nt | ut | Dt | nc | uc | Dc |
|---|---|---|---|---|---|---|---|---|---|---|---|---|
| HIV-1 | 3417 | 4758.5 | 729.38 | 2355 | 4861.1 | 867.16 | 2164 | 4992.7 | 798.33 | 1763 | 4831.8 | 890.29 |
| HIV-2 | 3305 | 4742.8 | 751.62 | 2412 | 4854.2 | 860.94 | 2020 | 5031.2 | 802.16 | 1976 | 4868.6 | 845.16 |

The distance between these two sequences is 295.99. We evaluate whether or not our 12-dimensional natural vector is robust to genetic mutations, i.e., deletion, duplication, and inversion. All simulated mutations will occur in HIV-1 viral genome.

### 4.1.1 One-letter deletion: 5 mutated sequences from HIV-1.

HIV-1(-2): the 2nd base is deleted from the HIV-1 genome sequence.
HIV-1(-2002): the 2002nd base is deleted from the HIV-1 genome sequence.
HIV-1(-4002): the 4002nd base is deleted from the HIV-1 genome sequence.
HIV-1(-6002): the 6002nd base is deleted from the HIV-1 genome sequence.
HIV-1(-8002): the 8002nd base is deleted from the HIV-1 genome sequence.

Distance Matrix for these 7 sequences:

| | HIV-1 | HIV-1(-2) | HIV-1(-2002) | HIV-1(-4002) | HIV-1(-6002) | HIV-1(-8002) | HIV-2 |
|---|---|---|---|---|---|---|---|
| HIV-1 | 0 | 2.34 | 1.79 | 1.48 | 1.64 | 1.86 | 295.99 |
| HIV-1(-2) | 2.34 | 0 | 2.88 | 1.70 | 2.28 | 3.00 | 296.41 |
| HIV-1(-2002) | 1.79 | 2.88 | 0 | 1.97 | 1.88 | 1.98 | 295.46 |
| HIV-1(-4002) | 1.48 | 1.70 | 1.97 | 0 | 1.62 | 1.53 | 296.29 |
| HIV-1(-6002) | 1.64 | 2.28 | 1.88 | 1.62 | 0 | 2.06 | 296.91 |
| HIV-1(-8002) | 1.86 | 3.00 | 1.98 | 1.53 | 2.06 | 0 | 296.18 |
| HIV-2 | 295.99 | 296.41 | 295.46 | 296.29 | 296.91 | 296.18 | 0 |

The five one-letter-mutated sequences are still very close the original HIV-1 virus sequence.

### 4.1.2 Two-letter deletion: 5 mutated sequences from HIV-1.

HIV-1(-2,3): the 2nd and 3rd bases are deleted from the HIV-1 genome sequence.
HIV-1(-2002,3): the 2002nd and 2003rd bases are deleted from the HIV-1 genome sequence.
HIV-1(-4002,3): the 4002nd and 4003rd bases are deleted from the HIV-1 genome sequence.
HIV-1(-6002,3): the 6002nd and 6003rd bases are deleted from the HIV-1 genome sequence.

HIV-1(-8002,3): the 8002nd and 8003rd bases are deleted from the HIV-1 genome sequence.

Distance Matrix for these 7 sequences:

| | HIV-1 | HIV-1(-2,3) | HIV-1(-2002,3) | HIV-1(-4002,3) | HIV-1(-6002,3) | HIV-1(-8002,3) | HIV-2 |
|---|---|---|---|---|---|---|---|
| HIV-1 | 0 | 4.69 | 2.69 | 2.48 | 2.59 | 3.72 | 295.99 |
| HIV-1(-2,3) | 4.69 | 0 | 3.59 | 3.94 | 4.24 | 6.01 | 296.85 |
| HIV-1(-2002,3) | 2.69 | 3.59 | 0 | 2.07 | 2.22 | 3.53 | 295.81 |
| HIV-1(-4002,3) | 2.48 | 3.94 | 2.07 | 0 | 1.70 | 2.97 | 297.12 |
| HIV-1(-6002,3) | 2.59 | 4.24 | 2.22 | 1.70 | 0 | 3.64 | 296.57 |
| HIV-1(-8002,3) | 3.72 | 6.01 | 3.53 | 2.97 | 3.64 | 0 | 296.38 |
| HIV-2 | 295.99 | 296.85 | 295.81 | 297.12 | 296.57 | 296.38 | 0 |

The five two-letters-mutated sequences are still very close the original HIV-1 virus sequence.

### 4.1.3 Three-letter deletion: 5 mutated sequences from HIV-1.

HIV-1(-2_4): from 2nd to 4th bases are deleted from the HIV-1 genome sequence.
HIV-1(-2002_4): from 2002nd to 2004th bases are deleted from the HIV-1 genome sequence.
HIV-1(-4002_4): from 4002nd to 4004th bases are deleted from the HIV-1 genome sequence.
HIV-1(-6002_4): from 6002nd to 6004th bases are deleted from the HIV-1 genome sequence.
HIV-1(-8002_4): from 8002nd to 8004th bases are deleted from the HIV-1 genome sequence.

Distance Matrix for these 7 sequences:

| | HIV-1 | HIV-1(-2_4) | HIV-1(-2002_4) | HIV-1(-4002_4) | HIV-1(-6002_4) | HIV-1(-8002_4) | HIV-2 |
|---|---|---|---|---|---|---|---|
| HIV-1 | 0 | 5.31 | 3.45 | 3.42 | 3.59 | 4.29 | 295.99 |
| HIV-1(-2_4) | 5.31 | 0 | 4.47 | 4.15 | 3.96 | 5.66 | 296.78 |
| HIV-1(-2002_4) | 3.45 | 4.47 | 0 | 1.03 | 2.14 | 3.14 | 296.48 |
| HIV-1(-4002_4) | 3.42 | 4.15 | 1.03 | 0 | 1.62 | 2.97 | 296.67 |
| HIV-1(-6002_4) | 3.59 | 3.96 | 2.14 | 1.62 | 0 | 2.59 | 296.82 |
| HIV-1(-8002_4) | 4.29 | 5.66 | 3.14 | 2.97 | 2.59 | 0 | 296 |
| HIV-2 | 295.99 | 296.78 | 296.48 | 296.67 | 296.82 | 296 | 0 |

The five three-letters-mutated sequences are still very close the original HIV-1 virus sequence.

### 4.1.4 50-letter deletion: 5 mutated sequences from HIV-1.

HIV-1(-2_51): from 2nd to 51st bases are deleted from the HIV-1 genome sequence.
HIV-1(-2002_51): from 2002nd to 2051st bases are deleted from the HIV-1 genome sequence.
HIV-1(-4002_51): from 4002nd to 4051st bases are deleted from the HIV-1 genome sequence.
HIV-1(-6002_51): from 6002nd to 6051st bases are deleted from the HIV-1 genome sequence.
HIV-1(-8002_51): from 8002nd to 8051st bases are deleted from the HIV-1 genome sequence.

Distance Matrix for these 7 sequences:

| | HIV-1 | HIV-1(-2_51) | HIV-1(-2002_51) | HIV-1(-4002_51) | HIV-1(-6002_51) | HIV-1(-8002_51) | HIV-2 |
|---|---|---|---|---|---|---|---|
| HIV-1 | 0 | 57.21 | 58.90 | 57.09 | 57.35 | 58.35 | 295.99 |
| HIV-1(-2_51) | 57.21 | 0 | 10.82 | 7.28 | 8.16 | 11.50 | 302.83 |
| HIV-1(-2002_51) | 58.90 | 10.82 | 0 | 14.82 | 16.03 | 16.96 | 305.75 |
| HIV-1(-4002_51) | 57.09 | 7.28 | 14.82 | 0 | 5.48 | 9.92 | 301.55 |
| HIV-1(-6002_51) | 57.35 | 8.16 | 16.03 | 5.48 | 0 | 9.93 | 302.43 |
| HIV-1(-8002_51) | 58.35 | 11.50 | 16.96 | 9.92 | 9.93 | 0 | 304.63 |
| HIV-2 | 295.99 | 302.83 | 305.75 | 301.55 | 302.43 | 304.63 | 0 |

### 4.1.5 100-letter deletion: 5 mutated sequences from HIV-1.

HIV-1(-2_101): from 2nd to 101st bases are deleted from the HIV-1 genome sequence.
HIV-1(-2002_101): from 2002nd to 2101st bases are deleted from the HIV-1 genome sequence.
HIV-1(-4002_101): from 4002nd to 4101st bases are deleted from the HIV-1 genome sequence.
HIV-1(-6002_101): from 6002nd to 6101st bases are deleted from the HIV-1 genome sequence.
HIV-1(-8002_101): from 8002nd to 8101st bases are deleted from the HIV-1 genome sequence.

Distance Matrix for these 7 sequences:

| | HIV-1 | HIV-1(-2_101) | HIV-1(-2002_101) | HIV-1(-4002_101) | HIV-1(-6002_101) | HIV-1(-8002_101) | HIV-2 |
|---|---|---|---|---|---|---|---|
| HIV-1 | 0 | 112.30 | 115.30 | 114.28 | 114.84 | 115.14 | 295.99 |
| HIV-1(-2_101) | 112.30 | 0 | 31.83 | 22.24 | 25.71 | 24.65 | 322.06 |
| HIV-1(-2002_101) | 115.30 | 31.83 | 0 | 15.79 | 19.81 | 22.05 | 318.66 |
| HIV-1(-4002_101) | 114.28 | 22.24 | 15.79 | 0 | 10.38 | 14.17 | 317 |
| HIV-1(-6002_101) | 114.84 | 25.71 | 19.81 | 10.38 | 0 | 19.34 | 313.26 |
| HIV-1(-8002_101) | 115.14 | 24.65 | 22.05 | 14.17 | 19.34 | 0 | 318.60 |
| HIV-2 | 295.99 | 322.06 | 318.66 | 317 | 313.26 | 318.60 | 0 |

The ten 50-letters and 100-letters-mutated sequences are still close the original HIV-1 virus sequence. However, the distances become larger significantly.

### 4.2.1 One-letter Duplication: 5 mutated sequences from HIV-1.

HIV-1(-2): the 2nd base is duplicated from the HIV-1 genome sequence.
HIV-1(-2002): the 2002nd base is duplicated from the HIV-1 genome sequence.
HIV-1(-4002): the 4002nd base is duplicated from the HIV-1 genome sequence.
HIV-1(-6002): the 6002nd base is duplicated from the HIV-1 genome sequence.
HIV-1(-8002): the 8002nd base is duplicated from the HIV-1 genome sequence.

Distance Matrix for these 7 sequences:

| | HIV-1 | HIV-1(-2) | HIV-1(-2002) | HIV-1(-4002) | HIV-1(-6002) | HIV-1(-8002) | HIV-2 |
|---|---|---|---|---|---|---|---|
| HIV-1 | 0 | 2.34 | 1.79 | 1.48 | 1.64 | 1.86 | 295.99 |
| HIV-1(-2) | 2.34 | 0 | 2.88 | 1.70 | 2.28 | 3.00 | 295.58 |
| HIV-1(-2002) | 1.79 | 2.88 | 0 | 1.97 | 1.88 | 1.97 | 296.53 |

| | | | | | | | |
|---|---|---|---|---|---|---|---|
| HIV-1(-4002) | 1.48 | 1.70 | 1.97 | 0 | 1.62 | 1.53 | 295.69 |
| HIV-1(-6002) | 1.64 | 2.28 | 1.88 | 1.62 | 0 | 2.06 | 295.06 |
| HIV-1(-8002) | 1.86 | 3.00 | 1.97 | 1.53 | 2.06 | 0 | 295.80 |
| HIV-2 | 295.99 | 295.58 | 296.53 | 295.69 | 295.06 | 295.80 | 0 |

### 4.2.2 Two-letter Duplication: 5 mutated sequences from HIV-1.

HIV-1(-2,3): the 2nd and 3rd bases are duplicated from the HIV-1 genome sequence.
HIV-1(-2002,3): the 2002nd and 2003rd bases are duplicated from the HIV-1 genome sequence.
HIV-1(-4002,3): the 4002nd and 4003rd bases are duplicated from the HIV-1 genome sequence.
HIV-1(-6002,3): the 6002nd and 6003rd bases are duplicated from the HIV-1 genome sequence.
HIV-1(-8002,3): the 8002nd and 8003rd bases are duplicated from the HIV-1 genome sequence.

Distance Matrix for these 7 sequences:

| | HIV-1 | HIV-1(-2,3) | HIV-1(-2002,3) | HIV-1(-4002,3) | HIV-1(-6002,3) | HIV-1(-8002,3) | HIV-2 |
|---|---|---|---|---|---|---|---|
| HIV-1 | 0 | 4.68 | 2.69 | 2.48 | 2.59 | 3.71 | 295.99 |
| HIV-1(-2,3) | 4.68 | 0 | 3.59 | 3.93 | 4.24 | 6.00 | 295.19 |
| HIV-1(-2002,3) | 2.69 | 3.59 | 0 | 2.07 | 2.22 | 3.52 | 296.18 |
| HIV-1(-4002,3) | 2.48 | 3.93 | 2.07 | 0 | 1.70 | 2.97 | 294.87 |
| HIV-1(-6002,3) | 2.59 | 4.24 | 2.22 | 1.70 | 0 | 3.63 | 295.42 |
| HIV-1(-8002,3) | 3.71 | 6.00 | 3.52 | 2.97 | 3.63 | 0 | 295.63 |
| HIV-2 | 295.99 | 295.19 | 296.18 | 294.87 | 295.42 | 295.63 | 0 |

### 4.2.3 Three-letter Duplication: 5 mutated sequences from HIV-1.

HIV-1(-2_4): from 2nd to 4th bases are duplicated from the HIV-1 genome sequence.
HIV-1(-2002_4): from 2002nd to 2004th bases are duplicated from the HIV-1 genome sequence.
HIV-1(-4002_4): from 4002nd to 4004th bases are duplicated from the HIV-1 genome sequence.
HIV-1(-6002_4): from 6002nd to 6004th bases are duplicated from the HIV-1 genome sequence.
HIV-1(-8002_4): from 8002nd to 8004th bases are duplicated from the HIV-1 genome sequence.

Distance Matrix for these 7 sequences:

| | HIV-1 | HIV-1(-2_4) | HIV-1(-2002_4) | HIV-1(-4002_4) | HIV-1(-6002_4) | HIV-1(-8002_4) | HIV-2 |
|---|---|---|---|---|---|---|---|
| HIV-1 | 0 | 5.31 | 3.45 | 3.42 | 3.59 | 4.29 | 295.99 |
| HIV-1(-2_4) | 5.31 | 0 | 4.47 | 4.15 | 3.96 | 5.65 | 295.29 |
| HIV-1(-2002_4) | 3.45 | 4.47 | 0 | 1.03 | 2.14 | 3.13 | 295.53 |
| HIV-1(-4002_4) | 3.42 | 4.15 | 1.03 | 0 | 1.62 | 2.96 | 295.34 |
| HIV-1(-6002_4) | 3.59 | 3.96 | 2.14 | 1.62 | 0 | 2.59 | 295.19 |
| HIV-1(-8002_4) | 4.29 | 5.65 | 3.13 | 2.96 | 2.59 | 0 | 296.03 |
| HIV-2 | 295.99 | 295.29 | 295.53 | 295.34 | 295.19 | 296.03 | 0 |

### 4.2.4 50-letter Duplication: 5 mutated sequences from HIV-1.

HIV-1(-2_51): from 2nd to 51st bases are duplicated from the HIV-1 genome sequence.
HIV-1(-2002_51): from 2002nd to 2051st bases are duplicated from the HIV-1 genome sequence.

HIV-1(-4002_51): from 4002nd to 4051st bases are duplicated from the HIV-1 genome sequence.
HIV-1(-6002_51): from 6002nd to 6051st bases are duplicated from the HIV-1 genome sequence.
HIV-1(-8002_51): from 8002nd to 8051st bases are duplicated from the HIV-1 genome sequence.

Distance Matrix for these 7 sequences:

| | HIV-1 | HIV-1(-2_51) | HIV-1(-2002_51) | HIV-1(-4002_51) | HIV-1(-6002_51) | HIV-1(-8002_51) | HIV-2 |
|---|---|---|---|---|---|---|---|
| HIV-1 | 0 | 57.21 | 58.91 | 57.09 | 57.35 | 58.31 | 295.99 |
| HIV-1(-2_51) | 57.21 | 0 | 10.74 | 7.21 | 8.14 | 11.41 | 300.10 |
| HIV-1(-2002_51) | 58.91 | 10.74 | 0 | 14.70 | 15.97 | 16.83 | 297.78 |
| HIV-1(-4002_51) | 57.09 | 7.21 | 14.70 | 0 | 5.46 | 9.88 | 301.33 |
| HIV-1(-6002_51) | 57.35 | 8.14 | 15.97 | 5.46 | 0 | 9.89 | 300.55 |
| HIV-1(-8002_51) | 58.31 | 11.41 | 16.83 | 9.88 | 9.89 | 0 | 298.70 |
| HIV-2 | 295.99 | 300.10 | 297.78 | 301.33 | 300.55 | 298.70 | 0 |

#### 4.2.5 100-letter Duplication: 5 mutated sequences from HIV-1.

HIV-1(-2_101): from 2nd to 101st bases are duplicated from the HIV-1 genome sequence.
HIV-1(-2002_101): from 2002nd to 2101st bases are duplicated from the HIV-1 genome sequence.
HIV-1(-4002_101): from 4002nd to 4101st bases are duplicated from the HIV-1 genome sequence.
HIV-1(-6002_101): from 6002nd to 6101st bases are duplicated from the HIV-1 genome sequence.
HIV-1(-8002_101): from 8002nd to 8101st bases are duplicated from the HIV-1 genome sequence.

Distance Matrix for these 7 sequences:

| | HIV-1 | HIV-1(-2_101) | HIV-1(-2002_101) | HIV-1(-4002_101) | HIV-1(-6002_101) | HIV-1(-8002_101) | HIV-2 |
|---|---|---|---|---|---|---|---|
| HIV-1 | 0 | 112.31 | 115.31 | 114.28 | 114.82 | 115.06 | 295.99 |
| HIV-1(-2_101) | 112.31 | 0 | 31.17 | 21.72 | 25.24 | 24.12 | 310.87 |
| HIV-1(-2002_101) | 115.31 | 31.17 | 0 | 15.56 | 19.73 | 21.66 | 316.66 |
| HIV-1(-4002_101) | 114.28 | 21.72 | 15.56 | 0 | 10.34 | 14.03 | 317.56 |
| HIV-1(-6002_101) | 114.82 | 25.24 | 19.73 | 10.34 | 0 | 19.20 | 321.65 |
| HIV-1(-8002_101) | 115.06 | 24.12 | 21.66 | 14.03 | 19.20 | 0 | 316.56 |
| HIV-2 | 295.99 | 310.87 | 316.66 | 317.56 | 321.65 | 316.56 | 0 |

The duplication-mutated sequences are still close the original HIV-1 virus sequence. The results are very similar to the deletion-mutated results.

#### 4.3.1 50-letter Inversion: 5 mutated sequences from HIV-1.

HIV-1(-2_51): from 2nd to 51st bases are inversed from the HIV-1 genome sequence.
HIV-1(-2002_51): from 2002nd to 2051st bases are inversed from the HIV-1 genome sequence.
HIV-1(-4002_51): from 4002nd to 4051st bases are inversed from the HIV-1 genome sequence.
HIV-1(-6002_51): from 6002nd to 6051st bases are inversed from the HIV-1 genome sequence.
HIV-1(-8002_51): from 8002nd to 8051st bases are inversed from the HIV-1 genome sequence.

Distance Matrix for these 7 sequences:

| | HIV-1 | HIV-1(-2_51) | HIV-1(-2002_51) | HIV-1(-4002_51) | HIV-1(-6002_51) | HIV-1(-8002_51) | HIV-2 |
|---|---|---|---|---|---|---|---|
| HIV-1 | 0 | 0.06 | 0.10 | 0.09 | 0.09 | 0.09 | 295.99 |
| HIV-1(-2_51) | 0.06 | 0 | 0.14 | 0.11 | 0.11 | 0.11 | 295.99 |
| HIV-1(-2002_51) | 0.10 | 0.14 | 0 | 0.12 | 0.08 | 0.11 | 295.96 |
| HIV-1(-4002_51) | 0.09 | 0.11 | 0.12 | 0 | 0.14 | 0.15 | 295.98 |
| HIV-1(-6002_51) | 0.09 | 0.12 | 0.08 | 0.14 | 0 | 0.06 | 295.98 |
| HIV-1(-8002_51) | 0.09 | 0.11 | 0.11 | 0.15 | 0.06 | 0 | 295.99 |
| HIV-2 | 295.99 | 295.99 | 295.96 | 295.98 | 295.98 | 295.99 | 0 |

### 4.3.2 100-letter Inversion: 5 mutated sequences from HIV-1.

HIV-1(-2_101): from 2nd to 101st bases are inversed from the HIV-1 genome sequence.
HIV-1(-2002_101): from 2002nd to 2101st bases are inversed from the HIV-1 genome sequence.
HIV-1(-4002_101): from 4002nd to 4101st bases are inversed from the HIV-1 genome sequence.
HIV-1(-6002_101): from 6002nd to 6101st bases are inversed from the HIV-1 genome sequence.
HIV-1(-8002_101): from 8002nd to 8101st bases are inversed from the HIV-1 genome sequence.

Distance Matrix for these 7 sequences:

| | HIV-1 | HIV-1(-2_101) | HIV-1(-2002_101) | HIV-1(-4002_101) | HIV-1(-6002_101) | HIV-1(-8002_101) | HIV-2 |
|---|---|---|---|---|---|---|---|
| HIV-1 | 0 | 0.34 | 0.32 | 0.10 | 0.32 | 0.30 | 295.99 |
| HIV-1(-2_101) | 0.34 | 0 | 0.57 | 0.39 | 0.57 | 0.49 | 296.05 |
| HIV-1(-2002_101) | 0.32 | 0.57 | 0 | 0.34 | 0.34 | 0.43 | 295.98 |
| HIV-1(-4002_101) | 0.11 | 0.39 | 0.34 | 0 | 0.28 | 0.28 | 295.97 |
| HIV-1(-6002_101) | 0.32 | 0.57 | 0.34 | 0.28 | 0 | 0.21 | 295.96 |
| HIV-1(-8002_101) | 0.30 | 0.49 | 0.42 | 0.28 | 0.21 | 0 | 295.97 |
| HIV-2 | 295.99 | 296.05 | 295.98 | 295.97 | 295.96 | 295.97 | 0 |

The inversion-mutation sequences are very close the original HIV-1 virus sequence.

### 4.4 Simultaneous Insertion and Deletion: 4 mutated sequences from HIV-1.

HIV-1(mutated 1): for the original HIV-1 genome sequence, delete a length-100 part (101st to 200th), then insert this part between 2000th and 2001st bases.
HIV-1(mutated 2): for the original HIV-1 genome sequence, delete a length-100 part (101st to 200th), then insert this part between 4000th and 4001st bases.
HIV-1(mutated 3): for the original HIV-1 genome sequence, delete a length-100 part (101st to 200th), then insert this part between 6000th and 6001st bases.
HIV-1(mutated 4): for the original HIV-1 genome sequence, delete a length-100 part (101st to 200th), then insert this part between 8000th and 8001st bases.

Distance Matrix for these 6 sequences:

| | HIV-1 | HIV-1(mutated 1) | HIV-1(mutated 2) | HIV-1(mutated 3) | HIV-1(mutated 4) | HIV-2 |
|---|---|---|---|---|---|---|
| HIV-1 | 0 | 3.58 | 9.25 | 14.72 | 21.60 | 295.99 |
| HIV-1(mutated 1) | 3.58 | 0 | 6.99 | 12.86 | 19.90 | 296.66 |
| HIV-1(mutated 2) | 9.25 | 6.99 | 0 | 6.16 | 13.81 | 296.78 |
| HIV-1(mutated 3) | 14.72 | 12.86 | 6.16 | 0 | 8.00 | 297.13 |
| HIV-1(mutated 4) | 21.60 | 19.90 | 13.81 | 8.00 | 0 | 298.26 |
| HIV-2 | 295.99 | 296.66 | 296.78 | 297.13 | 298.26 | 0 |

The mutated sequences are very close to the original HIV-1 virus sequence.

We also use the randomly simulated sequences to evaluate our 12-dimensional genome space. For sequence (1): HIV-1 E9 from the USA, complete genome (AF070521, GI: 3378121, Length: 9699). We generate 1000 randomly simulated sequences which have the same length as (1) and the equal probabilities of nucleotides (Length: 9699). We calculate the Euclidean distances between HIV-1 (1) and these 1000 simulated sequences. The result is shown in **Supplementary Table 11**. For the length-preserving simulated sequences, the natural vector can distinguish them easily from the original virus given significant distance differences.

**Supplementary Table 11**: The statistical results of the distances between HIV-1 and 1000 random simulated sequences.

| | | HIV-1 |
|---|---|---|
| 1000 random simulated sequences which have the same length as HIV-1 and equal probabilities of nucleotides. | Minimum distance | 1078.58 |
| | Median distance | 1247.36 |
| | Maximum distance | 1406.21 |
| | Average distance | 1248.03 |

## Graph Descriptions of Baltimore I, II, IV, VI

**Examples of the graph descriptions below (Baltimore class I):**

In Level 1, the record “Graph 1-0006 : 0009 -> 0221 (2170.941218), 0221 -> 1241 (1959.370864), 1241 -> 0221 (1959.370864)” indicates (1) It's a level-1 graph consisting of three viruses, #0009, #0221, #1241. (Please see the list of virus information at the end of the supplementary materials for name, and other information of the labeled viruses.) (2) “0009 -> 0221 (2170.941218)” means there is a directed line from #0009 to #0221 indicating the nearest neighbor of #0009 is #0221. The distance between the two viruses is 2170.941218.

In Level 2, the record “Graph 2-0008: 1-0010 -> 1-0081 ([0385] -> [0383], 3726.253127), 1-0081 -> 1-0010 ([0383] -> [0385], 3726.253127)” indicates (1) It's a level-2 graph formed by connecting two level-1 graphs, 1-0010 and 1-0081. (2) “1-0010 -> 1-0081 ([0385] -> [0383], 3726.253127)” means the nearest neighbor of level-1 graph 1-0010 is graph 1-0081 and the two level-1 graphs are connected by a directed line from virus #0385 to virus #0383 with distance 3726.253127. (3) “1-0081 -> 1-0010 ([0383] -> [0385], 3726.253127)” means that the directed line in (2) is actually bi-directed since the nearest neighbor of level-1 graph 1-0081 is just graph 1-0010.

**Graph description of Baltimore class I:**

Level 1

=======

Graph 1-0001 : 0001 -> 1710 (1933.436337), 1031 -> 1690 (1436.383454), 1690 -> 1698 (518.386962), 1694 -> 1698 (264.543324), 1698 -> 1694 (264.543324), 1710 -> 1698 (1625.733130),

Graph 1-0002 : 0003 -> 0459 (30962.142779), 0459 -> 0003 (30962.142779),

Graph 1-0003 : 0005 -> 1174 (754.704034), 1174 -> 0005 (754.704034),

Graph 1-0004 : 0007 -> 0015 (1708.012035), 0015 -> 0016 (1122.534487), 0016 -> 0015 (1122.534487), 1054 -> 0015 (2708.848350),

Graph 1-0005 : 0008 -> 1697 (2290.337468), 0115 -> 0127 (889.842501), 0127 -> 0115 (889.842501), 1697 -> 0127 (1288.036269),

Graph 1-0006 : 0009 -> 0221 (2170.941218), 0221 -> 1241 (1959.370864), 1241 -> 0221 (1959.370864),

Graph 1-0007 : 0010 -> 0011 (979.607191), 0011 -> 0010 (979.607191),

Graph 1-0008 : 0012 -> 0013 (1586.463671), 0013 -> 0012 (1586.463671),

Graph 1-0009 : 0014 -> 1742 (847.156150), 1742 -> 0014 (847.156150),

Graph 1-0010 : 0017 -> 0940 (5688.317057), 0123 -> 0923 (4271.689514), 0385 -> 0387 (3379.498080), 0387 -> 0385 (3379.498080), 0923 -> 0385 (3924.600271), 0940 -> 0387 (4534.324500),

Graph 1-0011 : 0020 -> 1427 (1198.390543), 1427 -> 0020 (1198.390543),

Graph 1-0012 : 0021 -> 1129 (3032.700974), 1108 -> 1129 (455.282545), 1129 -> 1108 (455.282545),

Graph 1-0013 : 0023 -> 0979 (1887.219335), 0134 -> 1991 (1266.019988), 0979 -> 1978 (1630.982806), 1783 -> 1978 (2489.139648), 1978 -> 1991 (1155.485199), 1991 -> 1978 (1155.485199),

Graph 1-0014 : 0032 -> 0034 (1760.457763), 0034 -> 0032 (1760.457763), 0097 -> 0034 (5294.546163), 1494 -> 0032 (3466.529653),

Graph 1-0015 : 0033 -> 0359 (6183.546794), 0359 -> 0999 (4450.222649), 0544 -> 1334 (9964.588634), 0708 -> 0033 (6954.353299), 0999 -> 0359 (4450.222649), 1334 -> 0033 (6311.622742),

Graph 1-0016 : 0038 -> 0560 (3856.287537), 0533 -> 0548 (2466.474892), 0548 -> 0560 (349.719755), 0560 -> 0548 (349.719755),

Graph 1-0017 : 0039 -> 0040 (756.226122), 0040 -> 0039 (756.226122),

Graph 1-0018 : 0041 -> 1977 (18011.245019), 1977 -> 0041 (18011.245019),

Graph 1-0019 : 0042 -> 1413 (2592.154597), 0715 -> 0042 (4201.628022), 1387 -> 1402 (200.591243), 1402 -> 1387 (200.591243), 1413 -> 1402 (1556.781130),

Graph 1-0020 : 0043 -> 1570 (127.064052), 1570 -> 0043 (127.064052),

Graph 1-0021 : 0046 -> 0531 (3708.222753), 0529 -> 0531 (991.722206), 0531 -> 0529 (991.722206), 0532 -> 0531 (1119.431993), 0722 -> 0532 (3440.691336),

Graph 1-0022 : 0060 -> 1614 (3097.247951), 0626 -> 1530 (4957.363205), 0997 -> 0626 (5823.314338), 1045 -> 0060 (6201.647098), 1530 -> 1614 (3342.773657), 1614 -> 0060 (3097.247951),

Graph 1-0023 : 0061 -> 0495 (2309.130995), 0129 -> 1615 (7399.099812), 0495 -> 1435 (2190.252386), 0724 -> 0726 (1153.904652), 0725 -> 0727 (280.937207), 0726 -> 0727 (651.705727), 0727 -> 0725 (280.937207), 0914 -> 0495 (6668.492222), 1435 -> 0726 (682.166222), 1615 -> 1435 (1900.421378),

Graph 1-0024 : 0062 -> 1618 (2822.427899), 1618 -> 0062 (2822.427899),

Graph 1-0025 : 0064 -> 0357 (3357.839817), 0081 -> 0861 (2157.058399), 0357 -> 0081 (2203.904583), 0627 -> 0861 (4505.749311), 0861 -> 0081 (2157.058399),

Graph 1-0026 : 0074 -> 1586 (1769.463779), 0657 -> 1586 (178.550440), 1586 -> 0657 (178.550440),

Graph 1-0027 : 0075 -> 1044 (5410.912535), 1044 -> 0075 (5410.912535),

Graph 1-0028 : 0078 -> 0806 (18437.936757), 0317 -> 0806 (9014.690007), 0806 -> 1441 (5636.196153), 1087 -> 1441 (3673.178982), 1088 -> 1087 (24323.045723), 1252 -> 0806 (12405.450059), 1441 -> 1087 (3673.178982),

Graph 1-0029 : 0080 -> 0358 (5451.069409), 0358 -> 0080 (5451.069409),

Graph 1-0030 : 0090 -> 1415 (1888.056760), 1412 -> 1419 (1614.647762), 1415 -> 1419 (504.442684), 1419 -> 1420 (89.896538), 1420 -> 1419 (89.896538),

Graph 1-0031 : 0102 -> 1335 (650.684747), 1335 -> 0102 (650.684747),

Graph 1-0032 : 0112 -> 1315 (2619.990444), 1106 -> 1150 (28.597516), 1115 -> 1106 (75.044774), 1150 -> 1106 (28.597516), 1169 -> 1115 (112.189609), 1315 -> 1150 (2253.256577),

Graph 1-0033 : 0113 -> 1237 (20116.499183), 0285 -> 0497 (4979.287066), 0424 -> 0872 (15799.075148), 0497 -> 0285 (4979.287066), 0872 -> 0497 (11415.043230), 1237 -> 0285 (11717.152064),

Graph 1-0034 : 0114 -> 0122 (685.922694), 0116 -> 0122 (138.449094), 0122 -> 0116 (138.449094), 0554 -> 0122 (1525.538679),

Graph 1-0035 : 0117 -> 1703 (1396.702327), 0132 -> 0972 (957.307981), 0972 -> 1629 (877.157585), 1629 -> 1709 (863.804372), 1701 -> 1703 (1294.747346), 1703 -> 1709 (1187.924258), 1709 -> 1629 (863.804372), 1782 -> 1629 (1346.754048),

Graph 1-0036 : 0118 -> 0121 (727.384870), 0119 -> 0121 (844.125362), 0121 -> 0118 (727.384870), 0381 -> 0119 (2202.828781), 0971 -> 0119 (858.481060),

Graph 1-0037 : 0120 -> 0934 (1854.384609), 0582 -> 0120 (1857.890885), 0934 -> 0936 (1107.908535), 0936 -> 0934 (1107.908535),

Graph 1-0038 : 0126 -> 0918 (1474.524542), 0915 -> 0918 (1032.276692), 0918 -> 0915 (1032.276692),

Graph 1-0039 : 0128 -> 0974 (5981.451193), 0911 -> 0974 (8383.028953), 0974 -> 0128 (5981.451193), 1651 -> 0128 (9930.756168), 2027 -> 0128 (7644.121428),

Graph 1-0040 : 0130 -> 0909 (2347.746790), 0551 -> 1985 (1771.002437), 0566 -> 1989 (1140.862173), 0611 -> 1989 (1786.756833), 0909 -> 0566 (1699.212505), 1985 -> 1989 (1516.256701), 1989 -> 0566 (1140.862173),

Graph 1-0041 : 0131 -> 1708 (952.326730), 0588 -> 1708 (1030.220943), 0920 -> 1708 (1249.965562), 1691 -> 1708 (982.631043), 1708 -> 0131 (952.326730),

Graph 1-0042 : 0133 -> 0674 (651.701294), 0674 -> 0133 (651.701294), 0913 -> 0133 (2059.591170),

Graph 1-0043 : 0135 -> 1649 (1515.152321), 1635 -> 1644 (1060.194522), 1637 -> 1644 (625.315470), 1644 -> 1637 (625.315470), 1649 -> 1644 (1370.738267),

Graph 1-0044 : 0146 -> 0147 (102.540064), 0147 -> 0146 (102.540064),

Graph 1-0045 : 0188 -> 1246 (238.696220), 0232 -> 0234 (230.904323), 0233 -> 0234 (214.418986), 0234 -> 0233 (214.418986), 0420 -> 0232 (243.542318), 0654 -> 0234 (243.749581), 1246 -> 0234 (222.234647),

Graph 1-0046 : 0195 -> 1230 (96.853269), 0283 -> 1176 (157.388577), 0884 -> 0195 (100.705499), 0898 -> 1623 (210.802207), 1176 -> 0884 (126.720477), 1230 -> 1569 (94.656569), 1551 -> 1569 (45.829489), 1569 -> 1551 (45.829489), 1623 -> 0195 (153.399930), 1930 -> 1230 (115.624944), 2014 -> 0195 (179.199502),

Graph 1-0047 : 0204 -> 0207 (1901.516752), 0207 -> 0204 (1901.516752), 0321 -> 0204 (5344.614706), 1234 -> 0207 (2172.456811), 1461 -> 1234 (3429.357084),

Graph 1-0048 : 0209 -> 0210 (45.233241), 0210 -> 0209 (45.233241), 0211 -> 0209 (175.326705),

Graph 1-0049 : 0219 -> 1928 (996.189614), 0771 -> 0219 (1115.054233), 0772 -> 0219 (1096.594544), 0773 -> 0772 (1197.014107), 0777 -> 0772 (1225.511356), 1344 -> 1928 (1243.002250), 1928 -> 0219 (996.189614),

Graph 1-0050 : 0220 -> 1549 (1141.736721), 1549 -> 1550 (675.703453), 1550 -> 1549 (675.703453),

Graph 1-0051 : 0226 -> 0228 (5396.486079), 0228 -> 0226 (5396.486079), 1741 -> 0228 (6933.136181),

Graph 1-0052 : 0227 -> 0514 (4509.307914), 0514 -> 1038 (2218.001232), 1038 -> 0514 (2218.001232),

Graph 1-0053 : 0235 -> 1218 (213.543661), 1218 -> 0235 (213.543661),

Graph 1-0054 : 0236 -> 1231 (6594.547759), 1231 -> 0236 (6594.547759), 1379 -> 1231 (6771.006852),

Graph 1-0055 : 0240 -> 1340 (300.601895), 1096 -> 1340 (354.179972), 1340 -> 1341 (104.002406), 1341 -> 1340 (104.002406),

Graph 1-0056 : 0253 -> 1394 (1357.811566), 0401 -> 1394 (4396.025526), 1142 -> 0253 (4846.384251), 1394 -> 0253 (1357.811566), 1400 -> 1394 (3181.067915),

Graph 1-0057 : 0254 -> 0258 (269.284995), 0257 -> 0258 (252.675706), 0258 -> 0257 (252.675706), 0265 -> 0257 (745.004934), 0270 -> 0254 (2778.703627), 2017 -> 0265 (1720.624903),

Graph 1-0058 : 0255 -> 1398 (2348.582474), 1396 -> 1414 (307.131394), 1398 -> 1396 (447.909660), 1414 -> 1396 (307.131394), 1440 -> 1396 (969.806553),

Graph 1-0059 : 0256 -> 0262 (1573.888788), 0262 -> 0256 (1573.888788), 1390 -> 0256 (2918.901802),

Graph 1-0060 : 0259 -> 1406 (1877.116000), 1406 -> 0259 (1877.116000), 1770 -> 0259 (1919.764830),

Graph 1-0061 : 0260 -> 1136 (2091.395464), 1107 -> 1113 (570.796811), 1113 -> 1136 (461.305432), 1136 -> 1113 (461.305432),

Graph 1-0062 : 0261 -> 0902 (3040.141763), 0902 -> 0261 (3040.141763),

Graph 1-0063 : 0263 -> 0266 (1194.744310), 0266 -> 1386 (1036.897876), 0269 -> 0271 (1360.371870), 0271 -> 1721 (1257.952767), 0273 -> 0271 (1291.608739), 0274 -> 1386 (1544.181686), 1384 -> 0266 (1088.372648), 1386 -> 1404 (841.350555), 1403 -> 1404 (321.605566), 1404 -> 1403 (321.605566), 1718 -> 1721 (2685.071051), 1721 -> 1404 (1029.143570),

Graph 1-0064 : 0264 -> 1575 (1419.168577), 0379 -> 0264 (2497.335124), 1385 -> 0264 (2376.566718), 1575 -> 0264 (1419.168577), 2021 -> 1385 (3660.048037),

Graph 1-0065 : 0267 -> 1389 (1473.753519), 0716 -> 1389 (1626.613828), 1389 -> 0267 (1473.753519), 1439 -> 1389 (1706.240549), 1684 -> 1389 (1591.538892),

Graph 1-0066 : 0268 -> 0272 (1686.234423), 0272 -> 0268 (1686.234423), 1109 -> 0272 (3628.526616),

Graph 1-0067 : 0284 -> 0663 (3807.184701), 0593 -> 0663 (5361.406668), 0663 -> 0284 (3807.184701),

Graph 1-0068 : 0287 -> 1267 (39386.615013), 0503 -> 1267 (45477.122509), 1037 -> 1267 (15313.503550), 1267 -> 1037 (15313.503550),

Graph 1-0069 : 0288 -> 1091 (973.112567), 1090 -> 1091 (747.895927), 1091 -> 1090 (747.895927),

Graph 1-0070 : 0292 -> 0297 (426.889320), 0297 -> 0610 (231.565002), 0547 -> 1378 (2117.280126), 0610 -> 0297 (231.565002), 1378 -> 0292 (1804.731668),

Graph 1-0071 : 0293 -> 0295 (174.601490), 0295 -> 0293 (174.601490),

Graph 1-0072 : 0294 -> 0632 (250.405652), 0632 -> 0294 (250.405652),

Graph 1-0073 : 0296 -> 1041 (187.735405), 0463 -> 0625 (338.442464), 0625 -> 1478 (318.331214), 1041 -> 0296 (187.735405), 1478 -> 0296 (247.979211),

Graph 1-0074 : 0300 -> 1432 (149.154766), 0609 -> 0300 (176.266949), 1432 -> 0300 (149.154766),

Graph 1-0075 : 0304 -> 0331 (100.376568), 0331 -> 0304 (100.376568),

Graph 1-0076 : 0319 -> 1261 (6805.002956), 1000 -> 1261 (2609.039757), 1261 -> 1000 (2609.039757),

Graph 1-0077 : 0320 -> 1001 (6600.553846), 1001 -> 0320 (6600.553846), 1388 -> 1001 (13173.954177), 1445 -> 0320 (11994.319687), 1446 -> 0320 (9441.995108),

Graph 1-0078 : 0361 -> 0723 (5363.595365), 0723 -> 1029 (2628.928571), 1029 -> 0723 (2628.928571), 1030 -> 1232 (3353.561113), 1232 -> 0723 (3350.831346), 1430 -> 1030 (4013.811754),

Graph 1-0079 : 0377 -> 1935 (2291.567232), 1935 -> 0377 (2291.567232),

Graph 1-0080 : 0382 -> 0998 (18022.809093), 0680 -> 0998 (6411.079488), 0998 -> 0680 (6411.079488),

Graph 1-0081 : 0383 -> 0386 (2968.553736), 0386 -> 0383 (2968.553736),

Graph 1-0082 : 0384 -> 0944 (2971.463122), 0942 -> 0944 (1386.401461), 0944 -> 0942 (1386.401461), 1743 -> 0384 (3488.094547),

Graph 1-0083 : 0397 -> 0824 (142.127772), 0823 -> 0397 (198.681259), 0824 -> 0397 (142.127772),

Graph 1-0084 : 0400 -> 2018 (1533.187503), 0901 -> 1383 (1580.543563), 1383 -> 0400 (1536.349952), 2018 -> 0400 (1533.187503),

Graph 1-0085 : 0427 -> 1070 (3397.817218), 0592 -> 0427 (11137.628517), 1070 -> 0427 (3397.817218), 1940 -> 1070 (10593.555644),

Graph 1-0086 : 0432 -> 0683 (275.592506), 0637 -> 1097 (255.932454), 0683 -> 1097 (184.522399), 1097 -> 0683 (184.522399),

Graph 1-0087 : 0439 -> 1326 (2898.919674), 1326 -> 0439 (2898.919674),

Graph 1-0088 : 0451 -> 0453 (2340.258050), 0453 -> 0451 (2340.258050), 0870 -> 0453 (5634.331313), 1147 -> 0870 (7523.857664),

Graph 1-0089 : 0455 -> 1101 (3339.013136), 0803 -> 0455 (5371.944432), 1101 -> 0455 (3339.013136),

Graph 1-0090 : 0464 -> 0465 (5263.228606), 0465 -> 0464 (5263.228606),

Graph 1-0091 : 0467 -> 0996 (5449.917897), 0665 -> 0996 (7749.571745), 0996 -> 0467 (5449.917897), 1423 -> 0996 (6387.351594),

Graph 1-0092 : 0473 -> 1089 (3709.692225), 1089 -> 0473 (3709.692225),

Graph 1-0093 : 0487 -> 1240 (1520.060210), 1240 -> 0487 (1520.060210),

Graph 1-0094 : 0496 -> 1266 (28228.845253), 1266 -> 1382 (7181.152708), 1382 -> 1266 (7181.152708), 1531 -> 1382 (18421.663657),

Graph 1-0095 : 0506 -> 2031 (702.197270), 0522 -> 2031 (1305.519217), 0564 -> 0506 (986.714948), 1075 -> 2031 (590.517177), 2031 -> 1075 (590.517177),

Graph 1-0096 : 0507 -> 0577 (978.632363), 0510 -> 0577 (3480.513675), 0539 -> 1724 (1765.146822), 0577 -> 0507 (978.632363), 1724 -> 0507 (1171.922697),

Graph 1-0097 : 0509 -> 0903 (426.658983), 0575 -> 0509 (426.799927), 0903 -> 0509 (426.658983), 1472 -> 0575 (1279.345124),

Graph 1-0098 : 0512 -> 0516 (1490.203624), 0516 -> 0542 (1427.323184), 0537 -> 0512 (2078.667204), 0542 -> 1399 (1341.607570), 1399 -> 1585 (1301.696038), 1408 -> 1399 (1642.969868), 1585 -> 1399 (1301.696038), 1773 -> 1399 (2665.515798), 1992 -> 1399 (1628.057480),

Graph 1-0099 : 0513 -> 2033 (515.280605), 0536 -> 2033 (444.445233), 0552 -> 0513 (800.330272), 0572 -> 1501 (856.998161), 1501 -> 0513 (636.805146), 2030 -> 0572 (1020.302906), 2033 -> 0536 (444.445233),

Graph 1-0100 : 0515 -> 0523 (697.514141), 0521 -> 1504 (1072.844145), 0523 -> 0515 (697.514141), 0567 -> 0515 (2591.311094), 1504 -> 0523 (1012.583070),

Graph 1-0101 : 0517 -> 0579 (2693.853684), 0579 -> 0517 (2693.853684), 0612 -> 0517 (4803.924893),

Graph 1-0102 : 0518 -> 0545 (1854.151726), 0545 -> 0518 (1854.151726),

Graph 1-0103 : 0530 -> 1498 (2308.925996), 0919 -> 1380 (2337.829514), 1321 -> 1407 (1317.545421), 1380 -> 1407 (563.744701), 1407 -> 1380 (563.744701), 1498 -> 1407 (1741.982720),

Graph 1-0104 : 0534 -> 0535 (1003.523563), 0535 -> 0534 (1003.523563),

Graph 1-0105 : 0541 -> 0614 (615.708431), 0565 -> 0614 (1250.457327), 0614 -> 0541 (615.708431),

Graph 1-0106 : 0543 -> 1474 (5616.704450), 0549 -> 1473 (6067.862720), 1473 -> 1474 (1645.921038), 1474 -> 1473 (1645.921038),

Graph 1-0107 : 0546 -> 1497 (868.035655), 1497 -> 0546 (868.035655), 1499 -> 0546 (1478.558795), 1503 -> 1499 (1887.229941),

Graph 1-0108 : 0550 -> 0576 (3961.575490), 0571 -> 1405 (1441.826202), 0576 -> 1405 (1730.851554), 0873 -> 0571 (1829.897165), 1405 -> 0571 (1441.826202),

Graph 1-0109 : 0555 -> 2032 (707.568103), 2032 -> 0555 (707.568103),

Graph 1-0110 : 0556 -> 0558 (567.412923), 0558 -> 0556 (567.412923), 0562 -> 0558 (4324.617490),

Graph 1-0111 : 0557 -> 0899 (3923.018967), 0559 -> 0557 (4326.794440), 0664 -> 0557 (4022.720463), 0899 -> 0557 (3923.018967),

Graph 1-0112 : 0561 -> 0573 (970.803754), 0573 -> 0561 (970.803754),

Graph 1-0113 : 0563 -> 2034 (1210.816886), 2034 -> 0563 (1210.816886),

Graph 1-0114 : 0569 -> 1502 (698.651182), 0570 -> 0569 (832.265505), 1502 -> 0569 (698.651182),

Graph 1-0115 : 0574 -> 1616 (3691.252508), 0590 -> 0574 (4589.335073), 1323 -> 0574 (5348.610149), 1616 -> 0574 (3691.252508),

Graph 1-0116 : 0583 -> 1672 (555.732141), 0975 -> 0583 (727.510552), 1630 -> 1641 (418.025227), 1631 -> 1679 (727.768857), 1632 -> 1640 (850.679855), 1640 -> 1672 (630.712972), 1641 -> 1672 (237.911697), 1645 -> 1641 (304.980516), 1646 -> 1630 (558.193657), 1647 -> 1672 (678.351503), 1648 -> 1641 (644.709993), 1653 -> 1648 (749.627602), 1655 -> 0583 (1054.220854), 1672 -> 1641 (237.911697), 1679 -> 1645 (494.364027),

Graph 1-0117 : 0584 -> 1650 (8474.952546), 1650 -> 0584 (8474.952546), 1678 -> 1650 (9374.383031),

Graph 1-0118 : 0585 -> 0587 (953.558720), 0587 -> 0585 (953.558720), 0922 -> 0587 (1080.947282), 1652 -> 0922 (1468.142469),

Graph 1-0119 : 0586 -> 0941 (830.403802), 0638 -> 0586 (1602.712599), 0928 -> 0586 (955.081585), 0930 -> 0948 (531.263040), 0941 -> 0930 (672.005394), 0946 -> 0930 (919.297385), 0948 -> 0930 (531.263040), 0970 -> 0946 (935.277772), 1744 -> 0638 (3021.862925),

Graph 1-0120 : 0591 -> 0594 (2262.934204), 0594 -> 0591 (2262.934204),

Graph 1-0121 : 0599 -> 0604 (35.752737), 0600 -> 0599 (350.269860), 0604 -> 0599 (35.752737), 0651 -> 0604 (227.101770), 0714 -> 0600 (422.475177), 1424 -> 1758 (391.682724), 1758 -> 0651 (292.243316),

Graph 1-0122 : 0615 -> 1617 (2920.052524), 0620 -> 1617 (4638.896359), 1573 -> 0615 (8289.836044), 1613 -> 0615 (3894.256872), 1617 -> 0615 (2920.052524),

Graph 1-0123 : 0647 -> 0648 (1837.941827), 0648 -> 0647 (1837.941827),

Graph 1-0124 : 0649 -> 1416 (22055.367850), 1416 -> 0649 (22055.367850),

Graph 1-0125 : 0656 -> 1944 (930.024492), 1944 -> 0656 (930.024492),

Graph 1-0126 : 0666 -> 0832 (45.373297), 0829 -> 0832 (113.956289), 0832 -> 0666 (45.373297),

Graph 1-0127 : 0681 -> 1528 (672.634583), 0995 -> 0681 (1480.186938), 1528 -> 0681 (672.634583),

Graph 1-0128 : 0711 -> 1699 (1671.141833), 1699 -> 1702 (1461.066516), 1702 -> 1699 (1461.066516),

Graph 1-0129 : 0712 -> 0713 (1008.390827), 0713 -> 0712 (1008.390827), 1055 -> 1270 (2352.612822), 1270 -> 0713 (1569.042788), 1375 -> 1426 (3939.757571), 1426 -> 1055 (2871.200495), 1700 -> 0713 (1494.348707), 1704 -> 0713 (1209.523990),

Graph 1-0130 : 0717 -> 0719 (2012.655873), 0719 -> 0717 (2012.655873), 1172 -> 0717 (4278.113123),

Graph 1-0131 : 0720 -> 1050 (123.784621), 1050 -> 0720 (123.784621),

Graph 1-0132 : 0728 -> 1239 (3530.210048), 0871 -> 0728 (6877.029027), 1238 -> 0871 (7097.497351), 1239 -> 0728 (3530.210048), 1772 -> 1238 (8389.012738),

Graph 1-0133 : 0752 -> 1752 (790.328978), 0753 -> 1751 (931.829333), 0932 -> 1752 (829.185388), 1745 -> 1746 (226.719595), 1746 -> 1745 (226.719595), 1747 -> 1746 (583.060320), 1751 -> 1747 (709.394802), 1752 -> 1745 (426.480202), 1754 -> 1747 (1017.541849),

Graph 1-0134 : 0770 -> 0775 (829.918287), 0775 -> 0770 (829.918287), 1343 -> 0775 (2829.601068),

Graph 1-0135 : 0774 -> 0776 (1045.052454), 0776 -> 0774 (1045.052454),

Graph 1-0136 : 0801 -> 0802 (4725.438769), 0802 -> 0801 (4725.438769),

Graph 1-0137 : 0804 -> 0805 (1085.854809), 0805 -> 0804 (1085.854809), 1002 -> 0805 (5015.150954),

Graph 1-0138 : 0807 -> 0808 (3174.633059), 0808 -> 0807 (3174.633059),

Graph 1-0139 : 0809 -> 1790 (8287.573189), 1790 -> 0809 (8287.573189),

Graph 1-0140 : 0810 -> 1244 (4524.440477), 1003 -> 1244 (4520.050188), 1244 -> 1003 (4520.050188),

Graph 1-0141 : 0815 -> 0818 (247.902703), 0816 -> 0833 (140.908813), 0818 -> 0836 (247.478802), 0825 -> 0836 (141.148554), 0826 -> 0833 (260.895445), 0833 -> 0836 (132.031069), 0836 -> 0833 (132.031069), 0837 -> 0825 (189.178972),

Graph 1-0142 : 0817 -> 0819 (242.334899), 0819 -> 1320 (180.460407), 0840 -> 0819 (189.738468), 1320 -> 0819 (180.460407), 1458 -> 0840 (271.893197),

Graph 1-0143 : 0820 -> 0821 (159.898787), 0821 -> 0820 (159.898787),

Graph 1-0144 : 0822 -> 0835 (307.348903), 0835 -> 0838 (140.870306), 0838 -> 0835 (140.870306),

Graph 1-0145 : 0827 -> 0834 (162.591981), 0834 -> 0827 (162.591981),

Graph 1-0146 : 0828 -> 1100 (153.937522), 1040 -> 1100 (98.853802), 1100 -> 1040 (98.853802), 1449 -> 1100 (189.520450),

Graph 1-0147 : 0830 -> 0841 (192.394036), 0831 -> 0830 (246.750776), 0839 -> 0842 (114.950830), 0841 -> 0842 (95.631011), 0842 -> 0841 (95.631011),

Graph 1-0148 : 0859 -> 1785 (2280.537076), 1785 -> 0859 (2280.537076),

Graph 1-0149 : 0862 -> 1233 (3301.566256), 1233 -> 0862 (3301.566256),

Graph 1-0150 : 0900 -> 1322 (1780.419331), 1322 -> 0900 (1780.419331),

Graph 1-0151 : 0910 -> 1706 (946.272447), 0921 -> 0910 (1243.373982), 0947 -> 1706 (741.765014), 1705 -> 1706 (1469.734853), 1706 -> 0947 (741.765014),

Graph 1-0152 : 0912 -> 1711 (1190.829513), 0977 -> 1693 (661.153224), 0978 -> 1695 (481.693246), 1692 -> 1693 (1490.864148), 1693 -> 1695 (304.820847), 1695 -> 1693 (304.820847), 1711 -> 0977 (1096.294221),

Graph 1-0153 : 0916 -> 0973 (844.925304), 0973 -> 0916 (844.925304), 1707 -> 0916 (1196.546266),

Graph 1-0154 : 0917 -> 1659 (1275.028778), 1627 -> 1658 (648.559322), 1658 -> 1663 (234.126657), 1659 -> 1658 (424.289759), 1663 -> 1658 (234.126657), 1669 -> 1658 (850.132507),

Graph 1-0155 : 0924 -> 0926 (1395.665776), 0926 -> 0939 (902.124995), 0927 -> 0924 (1500.847151), 0937 -> 0945 (1131.848505), 0939 -> 0945 (131.295181), 0943 -> 0937 (1949.503292), 0945 -> 0939 (131.295181),

Graph 1-0156 : 0925 -> 1626 (1103.694978), 1626 -> 1673 (370.455713), 1639 -> 1673 (1262.966558), 1673 -> 1626 (370.455713),

Graph 1-0157 : 0929 -> 1668 (1082.075657), 1657 -> 1660 (216.878528), 1660 -> 1657 (216.878528), 1668 -> 1660 (734.552702),

Graph 1-0158 : 0931 -> 0933 (840.861661), 0933 -> 1670 (805.957905), 1173 -> 1670 (812.812518), 1670 -> 0933 (805.957905),

Graph 1-0159 : 0935 -> 1628 (922.574938), 1628 -> 0935 (922.574938),

Graph 1-0160 : 0938 -> 1190 (4855.744716), 1189 -> 1191 (2751.307478), 1190 -> 1189 (3061.460361), 1191 -> 1189 (2751.307478), 1755 -> 0938 (5705.520831),

Graph 1-0161 : 0961 -> 1057 (8815.640135), 1057 -> 1179 (4854.527489), 1179 -> 1057 (4854.527489),

Graph 1-0162 : 0976 -> 1633 (2753.338481), 1633 -> 1680 (1414.999126), 1654 -> 1667 (512.001715), 1667 -> 1654 (512.001715), 1680 -> 1667 (952.252580),

Graph 1-0163 : 1056 -> 1114 (1867.683140), 1114 -> 1056 (1867.683140),

Graph 1-0164 : 1073 -> 1779 (1350.356716), 1771 -> 1963 (10313.567603), 1779 -> 1073 (1350.356716), 1963 -> 1073 (3278.833007), 1964 -> 1963 (10334.967449),

Graph 1-0165 : 1105 -> 1140 (1536.144318), 1126 -> 1140 (400.988857), 1140 -> 1126 (400.988857), 1158 -> 1126 (554.946087),

Graph 1-0166 : 1110 -> 1159 (1529.935123), 1139 -> 1159 (1520.177040), 1159 -> 1139 (1520.177040),

Graph 1-0167 : 1111 -> 1131 (777.607053), 1116 -> 1137 (558.261554), 1131 -> 1144 (189.724198), 1137 -> 1171 (494.781965), 1144 -> 1131 (189.724198), 1171 -> 1144 (311.333703),

Graph 1-0168 : 1112 -> 1133 (689.180376), 1123 -> 1170 (947.464660), 1133 -> 1170 (297.699213), 1149 -> 1170 (429.223089), 1162 -> 1170 (464.471953), 1170 -> 1133 (297.699213), 1459 -> 1123 (2859.089049),

Graph 1-0169 : 1117 -> 1132 (905.477588), 1119 -> 1132 (346.300332), 1132 -> 1119 (346.300332), 1145 -> 1117 (10066.353065), 1167 -> 1119 (596.027779),

Graph 1-0170 : 1118 -> 1151 (613.656241), 1151 -> 1118 (613.656241),

Graph 1-0171 : 1120 -> 1163 (185.005429), 1163 -> 1120 (185.005429), 1165 -> 1163 (284.377712),

Graph 1-0172 : 1121 -> 1148 (51.664061), 1148 -> 1121 (51.664061), 1152 -> 1121 (628.379529), 1153 -> 1148 (1045.963759),

Graph 1-0173 : 1122 -> 1141 (576.649700), 1138 -> 1160 (1550.894353), 1141 -> 1122 (576.649700), 1160 -> 1141 (952.317733),

Graph 1-0174 : 1124 -> 1422 (2339.932385), 1187 -> 1422 (1988.939829), 1401 -> 1422 (1280.221491), 1422 -> 1401 (1280.221491),

Graph 1-0175 : 1125 -> 1143 (1317.785358), 1143 -> 1157 (648.458402), 1157 -> 1143 (648.458402),

Graph 1-0176 : 1127 -> 1146 (1148.503579), 1146 -> 1127 (1148.503579),

Graph 1-0177 : 1128 -> 1154 (1908.816650), 1154 -> 1155 (410.092462), 1155 -> 1154 (410.092462),

Graph 1-0178 : 1130 -> 1166 (920.013373), 1166 -> 1130 (920.013373),

Graph 1-0179 : 1134 -> 1168 (1644.850245), 1168 -> 1134 (1644.850245),

Graph 1-0180 : 1135 -> 1156 (128.515186), 1156 -> 1135 (128.515186),

Graph 1-0181 : 1161 -> 1164 (326.026099), 1164 -> 1161 (326.026099),

Graph 1-0182 : 1178 -> 1722 (2234.097630), 1722 -> 1178 (2234.097630),

Graph 1-0183 : 1264 -> 1265 (15958.735052), 1265 -> 1264 (15958.735052),

Graph 1-0184 : 1373 -> 1934 (8537.040892), 1934 -> 1373 (8537.040892),

Graph 1-0185 : 1377 -> 2023 (2737.354848), 1774 -> 2023 (3778.631082), 2023 -> 1377 (2737.354848),

Graph 1-0186 : 1381 -> 1421 (350.566224), 1397 -> 1421 (299.869371), 1421 -> 1397 (299.869371),

Graph 1-0187 : 1391 -> 1409 (331.412004), 1393 -> 1409 (1537.409581), 1409 -> 1391 (331.412004),

Graph 1-0188 : 1392 -> 2024 (1571.296190), 1988 -> 1392 (1641.064969), 2024 -> 1392 (1571.296190),

Graph 1-0189 : 1395 -> 1719 (1455.335928), 1719 -> 1720 (524.106263), 1720 -> 1719 (524.106263),

Graph 1-0190 : 1634 -> 1696 (1138.643212), 1696 -> 1634 (1138.643212),

Graph 1-0191 : 1636 -> 1638 (354.372741), 1638 -> 1636 (354.372741),

Graph 1-0192 : 1642 -> 1671 (528.903364), 1671 -> 1642 (528.903364), 1674 -> 1671 (565.541499),

Graph 1-0193 : 1643 -> 1661 (751.025129), 1661 -> 1665 (625.984501), 1665 -> 1666 (614.628320), 1666 -> 1665 (614.628320),

Graph 1-0194 : 1662 -> 1664 (337.702012), 1664 -> 1662 (337.702012),

Graph 1-0195 : 1723 -> 1725 (732.443255), 1725 -> 1723 (732.443255),

Graph 1-0196 : 1726 -> 1727 (1247.669024), 1727 -> 1726 (1247.669024),

Graph 1-0197 : 1748 -> 1753 (316.368430), 1749 -> 1750 (1247.861562), 1750 -> 1748 (1220.495382), 1753 -> 1748 (316.368430),

Graph 1-0198 : 1769 -> 1776 (2546.917506), 1776 -> 2026 (116.372395), 2026 -> 1776 (116.372395),

Graph 1-0199 : 1786 -> 1788 (2956.014299), 1788 -> 1786 (2956.014299),

Graph 1-0200 : 1787 -> 1789 (993.417147), 1789 -> 1787 (993.417147),

Graph 1-0201 : 1974 -> 1975 (252.663327), 1975 -> 1974 (252.663327),

Graph 1-0202 : 1986 -> 1987 (106.390056), 1987 -> 1986 (106.390056),

Graph 1-0203 : 2016 -> 2020 (813.185433), 2019 -> 2020 (262.750224), 2020 -> 2019 (262.750224),

Total Graphs For Level - 1 : 203

Level 2

=======

Graph 2-0001 : 1-0001 -> 1-0152 ([1698] -> [1693], 903.981896), 1-0042 -> 1-0001 ([0674] -> [1031], 1757.888374), 1-0152 -> 1-0001 ([1693] -> [1698], 903.981896),

Graph 2-0002 : 1-0002 -> 1-0094 ([0003] -> [1531], 32777.256059), 1-0068 -> 1-0183 ([1267] -> [1265], 27255.736612), 1-0094 -> 1-0183 ([1266] -> [1264], 19183.463878), 1-0124 -> 1-0094 ([1416] -> [1531], 30498.780352), 1-0183 -> 1-0094 ([1264] -> [1266], 19183.463878),

Graph 2-0003 : 1-0003 -> 1-0070 ([0005] -> [0547], 2341.190217), 1-0045 -> 1-0146 ([1246] -> [0828], 223.358183), 1-0070 -> 1-0072 ([0610] -> [0632], 373.286391), 1-0072 -> 1-0045 ([0294] -> [0232], 283.855664), 1-0074 -> 1-0146 ([0609] -> [1100], 179.936982), 1-0146 -> 1-0074 ([1100] -> [0609], 179.936982),

Graph 2-0004 : 1-0004 -> 1-0125 ([0015] -> [0656], 2038.617121), 1-0125 -> 1-0004 ([0656] -> [0015], 2038.617121), 1-0148 -> 1-0004 ([0859] -> [0016], 2750.459051),

Graph 2-0005 : 1-0005 -> 1-0156 ([1697] -> [0925], 1638.101083), 1-0011 -> 1-0197 ([1427] -> [1748], 1696.248130), 1-0034 -> 1-0133 ([0114] -> [0752], 960.486414), 1-0037 -> 1-0005 ([0120] -> [0008], 2445.993755), 1-0133 -> 1-0158 ([0932] -> [0933], 911.054725), 1-0156 -> 1-0190 ([1673] -> [1634], 1333.509788), 1-0158 -> 1-0133 ([0933] -> [0932], 911.054725), 1-0190 -> 1-0133 ([1696] -> [1754], 1161.495692), 1-0197 -> 1-0133 ([1753] -> [1754], 1256.079397), 1-0199 -> 1-0011 ([1788] -> [0020], 3226.228946),

Graph 2-0006 : 1-0006 -> 1-0082 ([0009] -> [0942], 2336.883153), 1-0082 -> 1-0129 ([0942] -> [0712], 1734.543457), 1-0129 -> 1-0082 ([0712] -> [0942], 1734.543457), 1-0155 -> 1-0006 ([0927] -> [0009], 2546.281003),

Graph 2-0007 : 1-0007 -> 1-0009 ([0010] -> [0014], 1419.814801), 1-0008 -> 1-0007 ([0013] -> [0011], 1655.484244), 1-0009 -> 1-0007 ([0014] -> [0010], 1419.814801),

Graph 2-0008 : 1-0010 -> 1-0081 ([0385] -> [0383], 3726.253127), 1-0081 -> 1-0010 ([0383] -> [0385], 3726.253127),

Graph 2-0009 : 1-0012 -> 1-0174 ([1108] -> [1401], 2202.972860), 1-0174 -> 1-0012 ([1401] -> [1108], 2202.972860),

Graph 2-0010 : 1-0013 -> 1-0097 ([1991] -> [1472], 1614.465555), 1-0038 -> 1-0095 ([0918] -> [1075], 1735.546165), 1-0095 -> 1-0097 ([0506] -> [0903], 734.340643), 1-0097 -> 1-0099 ([0509] -> [0536], 569.692755), 1-0099 -> 1-0097 ([0536] -> [0509], 569.692755), 1-0100 -> 1-0099 ([0515] -> [2033], 869.100928), 1-0103 -> 1-0107 ([1321] -> [1499], 1690.251139), 1-0105 -> 1-0099 ([0614] -> [2030], 1473.083685), 1-0107 -> 1-0100 ([1497] -> [0521], 1093.413312), 1-0150 -> 1-0100 ([0900] -> [1504], 1780.958810), 1-0202 -> 1-0099 ([1987] -> [0536], 2028.548292),

Graph 2-0011 : 1-0014 -> 1-0087 ([0034] -> [0439], 3139.493284), 1-0015 -> 1-0087 ([0359] -> [1326], 4897.964790), 1-0052 -> 1-0014 ([1038] -> [0032], 3961.219079), 1-0087 -> 1-0014 ([0439] -> [0034], 3139.493284),

Graph 2-0012 : 1-0016 -> 1-0078 ([0038] -> [1430], 4495.016007), 1-0078 -> 1-0016 ([1430] -> [0038], 4495.016007), 1-0138 -> 1-0016 ([0807] -> [0038], 8019.156782), 1-0139 -> 1-0078 ([0809] -> [0723], 8508.786854), 1-0161 -> 1-0016 ([1179] -> [0038], 5438.770715),

Graph 2-0013 : 1-0017 -> 1-0111 ([0040] -> [0557], 4805.941342), 1-0111 -> 1-0017 ([0557] -> [0040], 4805.941342), 1-0132 -> 1-0111 ([1239] -> [0559], 7024.698661), 1-0184 -> 1-0185 ([1934] -> [1377], 8733.207773), 1-0185 -> 1-0111 ([1377] -> [0899], 5312.322442),

Graph 2-0014 : 1-0018 -> 1-0077 ([0041] -> [1001], 21336.613410), 1-0028 -> 1-0077 ([0317] -> [1446], 9627.310250), 1-0077 -> 1-0028 ([1446] -> [0317], 9627.310250),

Graph 2-0015 : 1-0019 -> 1-0063 ([1402] -> [1403], 332.914299), 1-0063 -> 1-0019 ([1403] -> [1402], 332.914299), 1-0065 -> 1-0063 ([1389] -> [1384], 1616.534075), 1-0188 -> 1-0065 ([1988] -> [0716], 1645.549687),

Graph 2-0016 : 1-0020 -> 1-0046 ([0043] -> [1551], 139.369699), 1-0046 -> 1-0020 ([1551] -> [0043], 139.369699), 1-0055 -> 1-0046 ([1340] -> [1176], 189.516987), 1-0086 -> 1-0046 ([0432] -> [1176], 325.542153), 1-0131 -> 1-0046 ([1050] -> [1930], 202.495270),

Graph 2-0017 : 1-0021 -> 1-0110 ([0532] -> [0562], 4364.224138), 1-0090 -> 1-0112 ([0464] -> [0561], 15154.657203), 1-0110 -> 1-0112 ([0558] -> [0561], 2882.300427), 1-0112 -> 1-0110 ([0561] -> [0558], 2882.300427),

Graph 2-0018 : 1-0022 -> 1-0091 ([0997] -> [0467], 6183.336922), 1-0085 -> 1-0137 ([0592] -> [0804], 14311.451229), 1-0091 -> 1-0022 ([0467] -> [0997], 6183.336922), 1-0120 -> 1-0022 ([0591] -> [0997], 8782.417913), 1-0137 -> 1-0091 ([1002] -> [1423], 10797.055987),

Graph 2-0019 : 1-0023 -> 1-0079 ([0724] -> [1935], 2381.134732), 1-0025 -> 1-0047 ([0081] -> [1461], 3842.974270), 1-0029 -> 1-0025 ([0358] -> [0357], 5963.747123), 1-0031 -> 1-0079 ([1335] -> [1935], 2499.346109), 1-0039 -> 1-0122 ([0911] -> [0620], 8433.781107), 1-0047 -> 1-0023 ([0207] -> [0727], 3431.913538), 1-0079 -> 1-0023 ([1935] -> [0724], 2381.134732), 1-0089 -> 1-0047 ([1101] -> [1234], 5067.572870), 1-0092 -> 1-0089 ([1089] -> [0455], 5664.919600), 1-0115 -> 1-0047 ([1616] -> [0204], 3968.292351), 1-0117 -> 1-0122 ([0584] -> [0620], 12548.732200), 1-0122 -> 1-0025 ([1613] -> [0861], 4333.844538),

Graph 2-0020 : 1-0024 -> 1-0067 ([0062] -> [0663], 5375.936540), 1-0067 -> 1-0024 ([0663] -> [0062], 5375.936540),

Graph 2-0021 : 1-0026 -> 1-0088 ([0074] -> [0453], 3511.259680), 1-0088 -> 1-0026 ([0453] -> [0074], 3511.259680),

Graph 2-0022 : 1-0027 -> 1-0033 ([0075] -> [0872], 36532.416423), 1-0033 -> 1-0164 ([0285] -> [1779], 8756.740798), 1-0080 -> 1-0164 ([0680] -> [1964], 11236.687553), 1-0164 -> 1-0033 ([1779] -> [0285], 8756.740798),

Graph 2-0023 : 1-0030 -> 1-0058 ([1420] -> [1396], 309.697172), 1-0048 -> 1-0189 ([0211] -> [1719], 2027.224533), 1-0058 -> 1-0030 ([1396] -> [1420], 309.697172), 1-0061 -> 1-0058 ([1136] -> [1414], 1911.666075), 1-0189 -> 1-0030 ([1395] -> [1415], 1462.407562),

Graph 2-0024 : 1-0032 -> 1-0180 ([1169] -> [1156], 204.492728), 1-0059 -> 1-0032 ([0262] -> [0112], 3351.971100), 1-0180 -> 1-0032 ([1156] -> [1169], 204.492728),

Graph 2-0025 : 1-0035 -> 1-0191 ([1629] -> [1636], 924.070178), 1-0043 -> 1-0116 ([1637] -> [1653], 797.970008), 1-0116 -> 1-0191 ([1646] -> [1638], 598.435861), 1-0128 -> 1-0035 ([1702] -> [1782], 1492.546416), 1-0191 -> 1-0116 ([1638] -> [1646], 598.435861), 1-0192 -> 1-0191 ([1674] -> [1636], 857.214777),

Graph 2-0026 : 1-0036 -> 1-0119 ([0119] -> [0970], 948.721909), 1-0041 -> 1-0119 ([0588] -> [0946], 1038.309192), 1-0119 -> 1-0036 ([0970] -> [0119], 948.721909), 1-0151 -> 1-0119 ([0947] -> [0946], 1083.625272), 1-0153 -> 1-0036 ([0973] -> [0971], 973.106156),

Graph 2-0027 : 1-0040 -> 1-0104 ([0566] -> [0534], 1266.053698), 1-0104 -> 1-0040 ([0534] -> [0566], 1266.053698), 1-0108 -> 1-0113 ([0873] -> [0563], 1887.818690), 1-0113 -> 1-0104 ([2034] -> [0535], 1725.346727), 1-0114 -> 1-0040 ([1502] -> [0551], 1795.889574),

Graph 2-0028 : 1-0044 -> 1-0145 ([0146] -> [0827], 276.139618), 1-0053 -> 1-0147 ([1218] -> [0839], 229.680869), 1-0075 -> 1-0144 ([0304] -> [0822], 429.011280), 1-0126 -> 1-0145 ([0832] -> [0834], 168.410507), 1-0143 -> 1-0144 ([0821] -> [0822], 320.084514), 1-0144 -> 1-0145 ([0835] -> [0827], 169.302975), 1-0145 -> 1-0126 ([0834] -> [0832], 168.410507), 1-0147 -> 1-0145 ([0841] -> [0827], 210.024505),

Graph 2-0029 : 1-0049 -> 1-0050 ([0777] -> [1549], 1462.508648), 1-0050 -> 1-0134 ([1549] -> [0770], 1214.649660), 1-0093 -> 1-0049 ([1240] -> [0771], 1771.919497), 1-0102 -> 1-0049 ([0518] -> [1928], 1871.735511), 1-0134 -> 1-0135 ([0775] -> [0776], 1209.654698), 1-0135 -> 1-0134 ([0776] -> [0775], 1209.654698), 1-0201 -> 1-0049 ([1975] -> [1928], 2091.034199),

Graph 2-0030 : 1-0051 -> 1-0076 ([1741] -> [0319], 9371.030124), 1-0054 -> 1-0136 ([1379] -> [0801], 10368.005590), 1-0076 -> 1-0136 ([1000] -> [0802], 7208.310224), 1-0136 -> 1-0076 ([0802] -> [1000], 7208.310224),

Graph 2-0031 : 1-0056 -> 1-0130 ([1400] -> [0719], 3341.070253), 1-0106 -> 1-0056 ([1473] -> [1400], 4232.940127), 1-0130 -> 1-0056 ([0719] -> [1400], 3341.070253), 1-0165 -> 1-0130 ([1140] -> [0719], 6285.725068), 1-0166 -> 1-0056 ([1110] -> [1394], 3737.330912), 1-0200 -> 1-0130 ([1789] -> [1172], 6338.813885),

Graph 2-0032 : 1-0057 -> 1-0163 ([0265] -> [1114], 2228.326726), 1-0163 -> 1-0057 ([1114] -> [0265], 2228.326726),

Graph 2-0033 : 1-0060 -> 1-0098 ([1406] -> [1585], 2257.396015), 1-0084 -> 1-0098 ([2018] -> [1585], 1598.507765), 1-0098 -> 1-0203 ([1399] -> [2019], 1585.750333), 1-0123 -> 1-0203 ([0648] -> [2019], 2765.832407), 1-0203 -> 1-0098 ([2019] -> [1399], 1585.750333),

Graph 2-0034 : 1-0062 -> 1-0066 ([0261] -> [0272], 3255.564754), 1-0066 -> 1-0173 ([0272] -> [1138], 2607.097549), 1-0167 -> 1-0173 ([1111] -> [1122], 1072.020375), 1-0170 -> 1-0173 ([1151] -> [1122], 1013.996552), 1-0173 -> 1-0170 ([1122] -> [1151], 1013.996552), 1-0178 -> 1-0167 ([1166] -> [1116], 1207.409993), 1-0179 -> 1-0173 ([1168] -> [1138], 3455.656534), 1-0182 -> 1-0178 ([1178] -> [1166], 2415.377753),

Graph 2-0035 : 1-0064 -> 1-0175 ([1575] -> [1125], 2574.177719), 1-0168 -> 1-0175 ([1112] -> [1143], 1386.023248), 1-0175 -> 1-0168 ([1143] -> [1112], 1386.023248),

Graph 2-0036 : 1-0069 -> 1-0109 ([0288] -> [0555], 2509.420741), 1-0109 -> 1-0069 ([0555] -> [0288], 2509.420741),

Graph 2-0037 : 1-0071 -> 1-0073 ([0295] -> [0296], 243.005988), 1-0073 -> 1-0071 ([0296] -> [0295], 243.005988), 1-0121 -> 1-0071 ([0604] -> [0293], 267.411170), 1-0142 -> 1-0071 ([0817] -> [0295], 289.171506),

Graph 2-0038 : 1-0083 -> 1-0141 ([0824] -> [0833], 194.114553), 1-0141 -> 1-0083 ([0833] -> [0824], 194.114553),

Graph 2-0039 : 1-0096 -> 1-0195 ([0577] -> [1725], 3114.702582), 1-0195 -> 1-0196 ([1725] -> [1726], 1836.459551), 1-0196 -> 1-0195 ([1726] -> [1725], 1836.459551),

Graph 2-0040 : 1-0101 -> 1-0160 ([0517] -> [1191], 5193.582583), 1-0160 -> 1-0101 ([1191] -> [0517], 5193.582583),

Graph 2-0041 : 1-0118 -> 1-0159 ([0587] -> [0935], 1006.895750), 1-0159 -> 1-0118 ([0935] -> [0587], 1006.895750),

Graph 2-0042 : 1-0127 -> 1-0198 ([0681] -> [1769], 5446.289898), 1-0198 -> 1-0127 ([1769] -> [0681], 5446.289898),

Graph 2-0043 : 1-0140 -> 1-0149 ([1003] -> [1233], 5434.308439), 1-0149 -> 1-0140 ([1233] -> [1003], 5434.308439),

Graph 2-0044 : 1-0154 -> 1-0157 ([1659] -> [1657], 462.835599), 1-0157 -> 1-0154 ([1657] -> [1659], 462.835599),

Graph 2-0045 : 1-0162 -> 1-0193 ([1654] -> [1666], 756.002022), 1-0193 -> 1-0162 ([1666] -> [1654], 756.002022), 1-0194 -> 1-0193 ([1662] -> [1643], 893.373438),

Graph 2-0046 : 1-0169 -> 1-0171 ([1167] -> [1165], 1032.906777), 1-0171 -> 1-0169 ([1165] -> [1167], 1032.906777),

Graph 2-0047 : 1-0172 -> 1-0177 ([1152] -> [1155], 874.100734), 1-0176 -> 1-0177 ([1146] -> [1154], 1152.718239), 1-0177 -> 1-0172 ([1155] -> [1152], 874.100734), 1-0181 -> 1-0172 ([1164] -> [1153], 1451.815786),

Graph 2-0048 : 1-0186 -> 1-0187 ([1381] -> [1391], 544.170778), 1-0187 -> 1-0186 ([1391] -> [1381], 544.170778),

Total Graphs For Level - 2 : 48

Level 3

=======

Graph 3-0001 : 2-0001 -> 2-0026 (1-0152 -> 1-0041 ([1711] -> [0920], 1351.424769)), 2-0004 -> 2-0006 (1-0125 -> 1-0155 ([1944] -> [0937], 2546.416621)), 2-0005 -> 2-0004 (1-0037 -> 1-0004 ([0934] -> [0007], 2559.706552)), 2-0006 -> 2-0001 (1-0082 -> 1-0001 ([0944] -> [1698], 1970.052643)), 2-0026 -> 2-0041 (1-0151 -> 1-0118 ([0947] -> [0585], 1106.269141)), 2-0029 -> 2-0001 (1-0201 -> 1-0042 ([1975] -> [0913], 2178.205714)), 2-0036 -> 2-0029 (1-0069 -> 1-0049

([1091] -> [1344], 2578.299999)), 2-0041 -> 2-0026  (1-0118 -> 1-0151  ([0585] -> [0947], 1106.269141)),

Graph 3-0002 : 2-0002 -> 2-0014  (1-0124 -> 1-0018  ([1416] -> [1977], 43839.982369)), 2-0012 -> 2-0017  (1-0016 -> 1-0110  ([0533] -> [0562], 5425.202925)), 2-0013 -> 2-0017  (1-0111 -> 1-0021 ([0899] -> [0529], 4954.484804)), 2-0014 -> 2-0022  (1-0077 -> 1-0033  ([1388] -> [0113], 21907.513197)), 2-0017 -> 2-0013  (1-0021 -> 1-0111  ([0529] -> [0899], 4954.484804)), 2-0018 -> 2-0012  (1-0022 -> 1-0161  ([1045] -> [1057], 7014.872002)), 2-0020 -> 2-0018  (1-0067 -> 1-0022 ([0663] -> [0060], 8293.523454)), 2-0022 -> 2-0013  (1-0164 -> 1-0132  ([1771] -> [1772], 11435.016520)), 2-0042 -> 2-0012  (1-0198 -> 1-0139  ([1769] -> [1790], 10785.708174)),

Graph 3-0003 : 2-0003 -> 2-0028  (1-0074 -> 1-0053  ([0609] -> [1218], 244.323150)), 2-0016 -> 2-0028  (1-0131 -> 1-0075  ([0720] -> [0331], 1814.534448)), 2-0028 -> 2-0003  (1-0053 -> 1-0074 ([1218] -> [0609], 244.323150)), 2-0037 -> 2-0003  (1-0073 -> 1-0146  ([1041] -> [0828], 284.208839)), 2-0038 -> 2-0028  (1-0141 -> 1-0147  ([0815] -> [0831], 276.118011)),

Graph 3-0004 : 2-0007 -> 2-0010  (1-0008 -> 1-0013  ([0012] -> [1783], 2682.338557)), 2-0010 -> 2-0027  (1-0107 -> 1-0040  ([1499] -> [1989], 1696.920664)), 2-0027 -> 2-0010  (1-0040 -> 1-0107 ([1989] -> [1499], 1696.920664)), 2-0032 -> 2-0033  (1-0057 -> 1-0060  ([2017] -> [1406], 2442.989718)), 2-0033 -> 2-0027  (1-0098 -> 1-0040  ([0542] -> [0611], 1865.245425)),

Graph 3-0005 : 2-0008 -> 2-0045  (1-0081 -> 1-0162  ([0386] -> [0976], 5244.441711)), 2-0025 -> 2-0044  (1-0116 -> 1-0157  ([0975] -> [1668], 1064.599341)), 2-0044 -> 2-0045  (1-0154 -> 1-0193 ([1658] -> [1643], 805.968221)), 2-0045 -> 2-0044  (1-0193 -> 1-0154  ([1643] -> [1658], 805.968221)),

Graph 3-0006 : 2-0009 -> 2-0035  (1-0174 -> 1-0064  ([1124] -> [1575], 2752.026831)), 2-0024 -> 2-0009  (1-0032 -> 1-0012  ([0112] -> [1129], 3388.598069)), 2-0031 -> 2-0048  (1-0166 -> 1-0186 ([1139] -> [1397], 4167.954802)), 2-0034 -> 2-0035  (1-0066 -> 1-0064  ([0268] -> [1385],

2663.672853)), 2-0035 -> 2-0034 (1-0064 -> 1-0066 ([1385] -> [0268], 2663.672853)), 2-0039 -> 2-0035 (1-0096 -> 1-0064 ([0510] -> [2021], 4324.503928)), 2-0040 -> 2-0031 (1-0160 -> 1-0106 ([1755] -> [0549], 6340.883438)), 2-0047 -> 2-0024 (1-0172 -> 1-0059 ([1153] -> [1390], 5897.345272)), 2-0048 -> 2-0024 (1-0187 -> 1-0032 ([1393] -> [1169], 3718.036798)),

Graph 3-0007 : 2-0011 -> 2-0019 (1-0014 -> 1-0115 ([0032] -> [1323], 7057.176882)), 2-0019 -> 2-0043 (1-0029 -> 1-0140 ([0358] -> [1003], 6531.062131)), 2-0021 -> 2-0011 (1-0088 -> 1-0052 ([0451] -> [0227], 10584.777851)), 2-0043 -> 2-0019 (1-0140 -> 1-0029 ([1003] -> [0358], 6531.062131)),

Graph 3-0008 : 2-0015 -> 2-0023 (1-0065 -> 1-0189 ([1684] -> [1720], 1849.793791)), 2-0023 -> 2-0015 (1-0189 -> 1-0065 ([1720] -> [1684], 1849.793791)),

Graph 3-0009 : 2-0030 -> 2-0046 (1-0136 -> 1-0171 ([0801] -> [1165], 8384.632950)), 2-0046 -> 2-0030 (1-0171 -> 1-0136 ([1165] -> [0801], 8384.632950)),

Total Graphs For Level - 3 : 9

Level 4

=======

Graph 4-0001 : 3-0001 -> 3-0005 (2-0041 -> 2-0025 (1-0159 -> 1-0035 ([0935] -> [0972], 1279.602698))), 3-0003 -> 3-0001 (2-0003 -> 2-0005 (1-0003 -> 1-0133 ([0005] -> [0752], 2922.386415))), 3-0004 -> 3-0001 (2-0010 -> 2-0026 (1-0038 -> 1-0151 ([0918] -> [1705], 1824.135014))), 3-0005 -> 3-0001 (2-0025 -> 2-0041 (1-0035 -> 1-0159 ([0972] -> [0935], 1279.602698))), 3-0006 -> 3-0004 (2-0034 -> 2-0032 (1-0178 -> 1-0057 ([1130] -> [0270], 2945.295839))), 3-0008 -> 3-0004 (2-0015 -> 2-0010 (1-0188 -> 1-0105 ([2024] -> [0541], 2043.874559))),

Graph 4-0002 : 3-0002 -> 3-0007 (2-0020 -> 2-0019 (1-0067 -> 1-0122 ([0593] -> [1573], 9085.150778))), 3-0007 -> 3-0002 (2-0019 -> 2-0020 (1-0122 -> 1-0067 ([1573] -> [0593], 9085.150778))), 3-0009 -> 3-0002 (2-0046 -> 2-0018 (1-0169 -> 1-0091 ([1117] -> [1423], 10929.806145))),

Total Graphs For Level - 4 : 2

Level 5

=======

Graph 5-0001 : 4-0001 -> 4-0002 (3-0006 -> 3-0007 (2-0040 -> 2-0011 (1-0101 -> 1-0015 ([0579] -> [0544], 10551.866808)))), 4-0002 -> 4-0001 (3-0007 -> 3-0006 (2-0011 -> 2-0040 (1-0015 -> 1-0101 ([0544] -> [0579], 10551.866808)))),

Total Graphs For Level - 5 : 1

**Graph description of Baltimore class II:**

Level 1

=======

Graph 1-0001 : 0004 -> 0200 (234.911219), 0200 -> 1308 (181.125814), 0298 -> 1328 (369.924281), 0848 -> 1180 (331.800146), 1180 -> 0200 (272.869413), 1308 -> 0200 (181.125814), 1328 -> 1180 (348.498424),

Graph 1-0002 : 0006 -> 0065 (413.465633), 0065 -> 1082 (218.052653), 1080 -> 1081 (39.440275), 1081 -> 1082 (23.534984), 1082 -> 1081 (23.534984), 1083 -> 1080 (42.954825),

Graph 1-0003 : 0025 -> 0030 (52.330958), 0027 -> 0025 (145.981549), 0028 -> 0030 (104.397354), 0030 -> 0025 (52.330958),

Graph 1-0004 : 0026 -> 0104 (122.724569), 0103 -> 0104 (23.730099), 0104 -> 0103 (23.730099),

Graph 1-0005 : 0029 -> 0218 (131.435617), 0218 -> 0029 (131.435617),

Graph 1-0006 : 0031 -> 0155 (350.330105), 0155 -> 0031 (350.330105),

Graph 1-0007 : 0035 -> 0869 (353.690158), 0869 -> 1912 (333.774142), 1912 -> 1927 (114.293287), 1913 -> 1927 (62.941447), 1914 -> 1927 (50.071918), 1925 -> 1926 (113.295230), 1926 -> 1914 (107.056155), 1927 -> 1914 (50.071918),

Graph 1-0008 : 0036 -> 0079 (84.512417), 0079 -> 0036 (84.512417),

Graph 1-0009 : 0047 -> 1437 (32.598405), 0369 -> 1868 (118.996965), 0618 -> 1810 (40.669488), 0756 -> 0047 (35.460141), 1437 -> 1861 (30.221000), 1810 -> 1861 (20.942156), 1861 -> 1810 (20.942156), 1863 -> 1437 (49.120081), 1864 -> 0047 (50.010434), 1868 -> 1864 (55.951582), 1882 -> 1810 (32.559092),

Graph 1-0010 : 0050 -> 1892 (26.858514), 0053 -> 1856 (55.450234), 0054 -> 1892 (32.156046), 1604 -> 0054 (54.647792), 1621 -> 0050 (36.030862), 1805 -> 1837 (25.693894), 1834 -> 1869 (44.743825), 1837 -> 1892 (20.380245), 1841 -> 1892 (26.584678), 1856 -> 1837 (28.744676), 1869 -> 1892 (34.579097), 1892 -> 1837 (20.380245),

Graph 1-0011 : 0055 -> 1804 (29.797787), 1804 -> 1829 (19.445339), 1829 -> 1804 (19.445339),

Graph 1-0012 : 0056 -> 0058 (24.310495), 0058 -> 0056 (24.310495),

Graph 1-0013 : 0068 -> 0405 (37.494151), 0405 -> 0068 (37.494151), 0480 -> 1020 (97.610139), 1020 -> 0068 (40.711423),

Graph 1-0014 : 0073 -> 1255 (28.306218), 0413 -> 1255 (53.148656), 1024 -> 0073 (31.385084), 1053 -> 1255 (38.272824), 1255 -> 0073 (28.306218), 1842 -> 0073 (42.884230), 1855 -> 0073 (48.663732),

Graph 1-0015 : 0148 -> 2002 (51.755436), 0366 -> 2002 (155.134417), 1206 -> 2002 (46.367046), 2002 -> 1206 (46.367046),

Graph 1-0016 : 0165 -> 1580 (515.206987), 0352 -> 0355 (19.066960), 0355 -> 0352 (19.066960), 1580 -> 0352 (375.978209), 1609 -> 0355 (494.776617),

Graph 1-0017 : 0166 -> 0489 (121.171391), 0395 -> 0709 (123.901478), 0489 -> 1084 (20.297312), 0709 -> 1084 (90.786349), 1084 -> 0489 (20.297312), 1102 -> 0489 (59.812591), 1681 -> 0709 (107.542180), 1897 -> 1681 (160.590639),

Graph 1-0018 : 0170 -> 0339 (31.405258), 0339 -> 0170 (31.405258), 0340 -> 0339 (45.618915), 1822 -> 0170 (51.587164),

Graph 1-0019 : 0175 -> 1885 (107.702635), 1867 -> 1894 (42.159886), 1885 -> 1894 (44.362878), 1894 -> 1957 (30.732086), 1957 -> 1894 (30.732086),

Graph 1-0020 : 0176 -> 1951 (89.359285), 0768 -> 0176 (116.196318), 1951 -> 0176 (89.359285),

Graph 1-0021 : 0177 -> 0180 (65.584864), 0180 -> 1294 (46.958833), 1294 -> 1605 (29.363666), 1304 -> 1294 (61.545087), 1605 -> 1294 (29.363666),

Graph 1-0022 : 0189 -> 0417 (39.336557), 0408 -> 0417 (36.999036), 0417 -> 0408 (36.999036),

Graph 1-0023 : 0190 -> 0619 (55.957752), 0619 -> 1217 (32.700828), 0687 -> 1217 (26.109109), 1023 -> 0687 (41.461199), 1217 -> 0687 (26.109109), 1865 -> 1217 (33.294579),

Graph 1-0024 : 0192 -> 1051 (41.452955), 0403 -> 0192 (43.791254), 0414 -> 0418 (28.689810), 0418 -> 0414 (28.689810), 1051 -> 0418 (30.341771),

Graph 1-0025 : 0203 -> 0781 (189.014769), 0291 -> 0779 (195.886479), 0779 -> 0780 (71.064348), 0780 -> 0779 (71.064348), 0781 -> 0779 (128.635663), 0782 -> 0780 (90.866870), 0800 -> 0291 (253.531870),

Graph 1-0026 : 0206 -> 0312 (137.197740), 0312 -> 0468 (90.119319), 0468 -> 0312 (90.119319), 1353 -> 0206 (286.247323),

Graph 1-0027 : 0238 -> 0239 (393.329430), 0239 -> 0238 (393.329430),

Graph 1-0028 : 0282 -> 0363 (133.297083), 0337 -> 1515 (303.860787), 0363 -> 0282 (133.297083), 0365 -> 1515 (170.450327), 0368 -> 0282 (196.937458), 1515 -> 0363 (151.359081), 1578 -> 0363 (227.169783),

Graph 1-0029 : 0286 -> 1451 (105.079598), 0636 -> 0286 (109.741111), 1451 -> 0286 (105.079598), 1518 -> 0286 (192.460747),

Graph 1-0030 : 0330 -> 0605 (69.461338), 0364 -> 0330 (208.799394), 0605 -> 0330 (69.461338),

Graph 1-0031 : 0348 -> 0883 (43.899173), 0882 -> 1295 (119.891357), 0883 -> 1847 (34.167640), 0968 -> 0883 (35.332343), 1295 -> 1847 (29.555810), 1544 -> 1295 (39.699298), 1828 -> 1847 (40.294781), 1847 -> 1295 (29.555810),

Graph 1-0032 : 0351 -> 0354 (22.824101), 0353 -> 0354 (538.322108), 0354 -> 0351 (22.824101),

Graph 1-0033 : 0356 -> 1491 (65.933089), 1067 -> 1960 (129.573135), 1251 -> 1960 (73.814038), 1491 -> 1960 (46.349664), 1960 -> 1491 (46.349664),

Graph 1-0034 : 0362 -> 0367 (198.761595), 0367 -> 0362 (198.761595),

Graph 1-0035 : 0370 -> 1346 (171.942076), 0371 -> 0370 (178.008654), 0393 -> 0370 (534.004074), 0682 -> 1346 (112.783088), 1345 -> 1346 (51.846943), 1346 -> 1345 (51.846943),

Graph 1-0036 : 0380 -> 1832 (35.953419), 1832 -> 0380 (35.953419),

Graph 1-0037 : 0425 -> 1871 (32.151028), 0608 -> 1871 (29.414301), 1028 -> 0608 (38.783810), 1297 -> 0425 (42.510879), 1425 -> 1871 (40.389309), 1597 -> 1871 (30.139483), 1836 -> 1871 (22.036531), 1845 -> 1597 (31.559395), 1871 -> 1836 (22.036531),

Graph 1-0038 : 0430 -> 1849 (40.294821), 0896 -> 1849 (32.219054), 1026 -> 0896 (41.736643), 1800 -> 1843 (31.751234), 1843 -> 1849 (21.272802), 1849 -> 1843 (21.272802), 1854 -> 1843 (37.636096),

Graph 1-0039 : 0452 -> 0887 (236.351552), 0476 -> 0662 (121.720462), 0662 -> 0476 (121.720462), 0887 -> 0476 (210.014473), 1177 -> 0662 (128.594073),

Graph 1-0040 : 0477 -> 1014 (66.816543), 1014 -> 0477 (66.816543),

Graph 1-0041 : 0502 -> 1523 (49.730987), 1523 -> 1548 (45.417360), 1546 -> 1548 (28.755820), 1548 -> 1546 (28.755820), 1887 -> 1548 (39.209104),

Graph 1-0042 : 0508 -> 0578 (56.793228), 0568 -> 0508 (58.882449), 0578 -> 0508 (56.793228), 1411 -> 0578 (369.436545), 1972 -> 0508 (439.246790),

Graph 1-0043 : 0520 -> 0526 (141.888187), 0525 -> 0526 (145.194130), 0526 -> 0520 (141.888187), 0553 -> 0525 (383.278219),

Graph 1-0044 : 0524 -> 0528 (327.586760), 0528 -> 0524 (327.586760), 0538 -> 0524 (438.986486),

Graph 1-0045 : 0527 -> 1973 (475.775303), 1973 -> 1982 (396.539241), 1982 -> 1973 (396.539241),

Graph 1-0046 : 0606 -> 1737 (37.325748), 1735 -> 1737 (45.488471), 1737 -> 0606 (37.325748), 1738 -> 0606 (56.421973),

Graph 1-0047 : 0633 -> 1290 (298.925631), 1289 -> 1290 (83.063688), 1290 -> 1289 (83.063688),

Graph 1-0048 : 0685 -> 1103 (81.330106), 0847 -> 1103 (309.479199), 1103 -> 0685 (81.330106),

Graph 1-0049 : 0710 -> 0994 (94.822220), 0994 -> 1079 (71.347739), 1065 -> 1079 (67.843569), 1079 -> 1065 (67.843569),

Graph 1-0050 : 0718 -> 2015 (483.397749), 1374 -> 1444 (1090.373415), 1410 -> 2015 (326.495581), 1438 -> 2015 (573.366499), 1444 -> 1438 (727.821471), 2015 -> 1410 (326.495581),

Graph 1-0051 : 0761 -> 1884 (29.508430), 1306 -> 1811 (44.106396), 1811 -> 0761 (35.120618), 1884 -> 0761 (29.508430),

Graph 1-0052 : 0763 -> 1803 (50.525617), 1257 -> 1831 (46.512331), 1757 -> 1850 (34.198618), 1803 -> 1850 (27.363933), 1831 -> 1850 (31.099042), 1839 -> 1803 (37.179954), 1850 -> 1803 (27.363933),

Graph 1-0053 : 0764 -> 1807 (43.011141), 1282 -> 1860 (26.154954), 1807 -> 1282 (34.006483), 1860 -> 1282 (26.154954),

Graph 1-0054 : 0874 -> 1765 (32.017929), 1763 -> 1765 (43.601723), 1765 -> 0874 (32.017929),

Graph 1-0055 : 0991 -> 1541 (59.344936), 1536 -> 1541 (23.668701), 1541 -> 1536 (23.668701), 1599 -> 1536 (50.302094),

Graph 1-0056 : 1018 -> 1851 (50.193204), 1833 -> 1851 (35.776938), 1838 -> 1870 (42.927177), 1851 -> 1870 (20.846076), 1870 -> 1851 (20.846076), 1877 -> 1833 (101.227861),

Graph 1-0057 : 1061 -> 1852 (24.744018), 1852 -> 1061 (24.744018),

Graph 1-0058 : 1213 -> 1532 (241.366224), 1532 -> 1878 (49.592979), 1827 -> 1878 (62.195330), 1878 -> 1532 (49.592979),

Graph 1-0059 : 1215 -> 1858 (33.446844), 1858 -> 1215 (33.446844),

Graph 1-0060 : 1254 -> 1840 (23.811049), 1299 -> 1254 (33.577343), 1625 -> 1299 (36.096385), 1840 -> 1254 (23.811049), 1866 -> 1254 (34.407428), 1883 -> 1840 (40.695232), 1969 -> 1883 (79.757763),

Graph 1-0061 : 1442 -> 1443 (102.360168), 1443 -> 1442 (102.360168),

Graph 1-0062 : 1537 -> 1539 (19.058587), 1539 -> 1537 (19.058587),

Graph 1-0063 : 1579 -> 1901 (176.826526), 1900 -> 1902 (60.629152), 1901 -> 1903 (128.962194), 1902 -> 1904 (51.870761), 1903 -> 1900 (113.344366), 1904 -> 1907 (43.973091), 1906 -> 1904 (72.351705), 1907 -> 1904 (43.973091), 1910 -> 1901 (221.511012),

Graph 1-0064 : 1606 -> 1607 (234.352610), 1607 -> 1606 (234.352610), 1608 -> 1606 (486.548584), 1610 -> 1607 (1152.005246),

Graph 1-0065 : 1761 -> 1766 (35.146916), 1762 -> 1764 (22.629838), 1764 -> 1762 (22.629838), 1766 -> 1764 (32.408221),

Graph 1-0066 : 1853 -> 1875 (24.057726), 1875 -> 1853 (24.057726),

Graph 1-0067 : 1895 -> 1909 (239.885402), 1909 -> 1895 (239.885402),

Graph 1-0068 : 1896 -> 1920 (208.535697), 1915 -> 1920 (146.245943), 1918 -> 1919 (125.031473), 1919 -> 1921 (77.589992), 1920 -> 1919 (143.396846), 1921 -> 1919 (77.589992), 1922 -> 1924 (134.109760), 1924 -> 1921 (89.691375),

Graph 1-0069 : 1898 -> 1899 (90.537298), 1899 -> 1898 (90.537298), 1911 -> 1899 (402.532009),

Graph 1-0070 : 1905 -> 1908 (55.292044), 1908 -> 1905 (55.292044),

Graph 1-0071 : 1916 -> 1923 (56.731837), 1917 -> 1916 (110.249047), 1923 -> 1916 (56.731837),

Graph 1-0072 : 1976 -> 1990 (300.044717), 1979 -> 1990 (272.933754), 1983 -> 1976 (353.252792), 1990 -> 1979 (272.933754),

Graph 1-0073 : 1980 -> 1981 (2.266095), 1981 -> 1980 (2.266095), 1984 -> 1980 (548.576201),

Total Graphs For Level - 1 : 73

Level 2

=======

Graph 2-0001 : 1-0001 -> 1-0026 ([0298] -> [0468], 371.710577), 1-0002 -> 1-0026 ([0065] -> [0312], 251.156365), 1-0025 -> 1-0049 ([0780] -> [1065], 236.084306), 1-0026 -> 1-0049 ([0206] -> [1079], 193.919203), 1-0027 -> 1-0001 ([0239] -> [0848], 441.893192), 1-0039 -> 1-0001 ([0452] -> [1180], 408.045427), 1-0043 -> 1-0001 ([0525] -> [1180], 377.075583), 1-0047 -> 1-0039 ([0633] -> [0662], 782.298774), 1-0048 -> 1-0049 ([1103] -> [0994], 235.377023), 1-0049 -> 1-0026 ([1079] -> [0206], 193.919203),

Graph 2-0002 : 1-0003 -> 1-0004 ([0027] -> [0026], 177.878386), 1-0004 -> 1-0003 ([0026] -> [0027], 177.878386), 1-0005 -> 1-0004 ([0218] -> [0104], 208.068402), 1-0006 -> 1-0004 ([0031] -> [0026], 361.350559),

Graph 2-0003 : 1-0007 -> 1-0071 ([1914] -> [1923], 66.893630), 1-0008 -> 1-0007 ([0079] -> [0869], 381.948331), 1-0068 -> 1-0071 ([1921] -> [1923], 99.767359), 1-0071 -> 1-0007 ([1923] -> [1914], 66.893630),

Graph 2-0004 : 1-0009 -> 1-0010 ([1810] -> [1892], 23.238210), 1-0010 -> 1-0009 ([1892] -> [1810], 23.238210), 1-0012 -> 1-0010 ([0056] -> [1837], 44.243262), 1-0014 -> 1-0037 ([1255] -> [1836], 33.513535), 1-0019 -> 1-0010 ([1894] -> [1856], 36.762062), 1-0022 -> 1-0009 ([0408] -> [0756], 42.224551), 1-0024 -> 1-0052 ([0418] -> [1803], 33.495117), 1-0036 -> 1-0052 ([1832] -> [1757], 40.510804), 1-0037 -> 1-0010 ([1871] -> [0050], 28.319318), 1-0041 -> 1-0019 ([1887] -> [1957], 39.734769), 1-0052 -> 1-0009 ([1831] -> [1861], 31.819391), 1-0060 -> 1-0009 ([1840] -> [1810], 29.758384), 1-0062 -> 1-0014 ([1537] -> [1842], 102.655905),

Graph 2-0005 : 1-0011 -> 1-0038 ([1804] -> [1843], 32.353905), 1-0038 -> 1-0011 ([1843] -> [1804], 32.353905), 1-0051 -> 1-0038 ([0761] -> [0896], 33.937426), 1-0056 -> 1-0051 ([1870] -> [0761], 43.461621),

Graph 2-0006 : 1-0013 -> 1-0023 ([0405] -> [1865], 39.686555), 1-0023 -> 1-0053 ([0619] -> [1860], 32.940696), 1-0030 -> 1-0055 ([0330] -> [1536], 76.267047), 1-0031 -> 1-0023 ([0968] -> [0619], 35.986718), 1-0053 -> 1-0023 ([1860] -> [0619], 32.940696), 1-0055 -> 1-0031 ([1541] -> [1544], 45.808115),

Graph 2-0007 : 1-0015 -> 1-0040 ([0148] -> [0477], 91.942081), 1-0040 -> 1-0015 ([0477] -> [0148], 91.942081),

Graph 2-0008 : 1-0016 -> 1-0032 ([0352] -> [0351], 33.403900), 1-0032 -> 1-0016 ([0351] -> [0352], 33.403900),

Graph 2-0009 : 1-0017 -> 1-0029 ([0489] -> [0286], 112.196798), 1-0028 -> 1-0017 ([1515] -> [1681], 193.189453), 1-0029 -> 1-0017 ([0286] -> [0489], 112.196798), 1-0034 -> 1-0035 ([0362] -> [0371], 228.442702), 1-0035 -> 1-0029 ([0682] -> [1451], 133.482447),

Graph 2-0010 : 1-0018 -> 1-0058 ([1822] -> [1827], 93.964751), 1-0058 -> 1-0018 ([1827] -> [1822], 93.964751),

Graph 2-0011 : 1-0020 -> 1-0021 ([0176] -> [0177], 99.128153), 1-0021 -> 1-0020 ([0177] -> [0176], 99.128153),

Graph 2-0012 : 1-0033 -> 1-0046 ([1960] -> [1737], 63.389300), 1-0046 -> 1-0033 ([1737] -> [1960], 63.389300),

Graph 2-0013 : 1-0042 -> 1-0044 ([1972] -> [0538], 456.520869), 1-0044 -> 1-0072 ([0524] -> [1990], 379.617550), 1-0045 -> 1-0073 ([0527] -> [1980], 785.763401), 1-0050 -> 1-0072 ([0718] -> [1983], 648.024458), 1-0061 -> 1-0045 ([1443] -> [1982], 1423.726095), 1-0064 -> 1-0044 ([1610] -> [0528], 1299.844768), 1-0072 -> 1-0044 ([1990] -> [0524], 379.617550), 1-0073 -> 1-0072 ([1984] -> [1976], 572.309674),

Graph 2-0014 : 1-0054 -> 1-0065 ([0874] -> [1762], 38.360403), 1-0065 -> 1-0054 ([1762] -> [0874], 38.360403),

Graph 2-0015 : 1-0057 -> 1-0066 ([1061] -> [1853], 26.090577), 1-0059 -> 1-0066 ([1858] -> [1853], 51.515055), 1-0066 -> 1-0057 ([1853] -> [1061], 26.090577),

Graph 2-0016 : 1-0063 -> 1-0070 ([1907] -> [1905], 55.918622), 1-0067 -> 1-0063 ([1909] -> [1910], 285.595591), 1-0069 -> 1-0070 ([1899] -> [1908], 406.726136), 1-0070 -> 1-0063 ([1905] -> [1907], 55.918622),

Total Graphs For Level - 2 : 16

Level 3

=======

Graph 3-0001 : 2-0001 -> 2-0008  (1-0002 -> 1-0016  ([1083] -> [1580], 423.615513)), 2-0002 -> 2-0008  (1-0004 -> 1-0016  ([0026] -> [1580], 496.693223)), 2-0008 -> 2-0001  (1-0016 -> 1-0002 ([1580] -> [1083], 423.615513)), 2-0013 -> 2-0001  (1-0042 -> 1-0039  ([0508] -> [1177], 627.559548)),

Graph 3-0002 : 2-0003 -> 2-0016  (1-0068 -> 1-0069  ([1922] -> [1911], 419.298555)), 2-0004 -> 2-0015  (1-0009 -> 1-0057  ([1861] -> [1061], 31.749695)), 2-0005 -> 2-0004  (1-0011 -> 1-0052 ([1804] -> [1803], 33.279358)), 2-0006 -> 2-0015  (1-0053 -> 1-0066  ([1860] -> [1875], 33.978579)), 2-0007 -> 2-0006  (1-0015 -> 1-0055  ([1206] -> [0991], 110.821376)), 2-0009 -> 2-0010  (1-0028 -> 1-0058  ([1515] -> [1213], 294.370415)), 2-0010 -> 2-0004  (1-0058 -> 1-0062  ([1878] -> [1539], 111.367467)), 2-0011 -> 2-0004  (1-0021 -> 1-0019  ([0180] -> [0175], 125.517346)), 2-0012 -> 2-0004  (1-0033 -> 1-0062  ([1067] -> [1537], 140.966743)), 2-0014 -> 2-0015  (1-0054 -> 1-0066 ([0874] -> [1875], 47.424032)), 2-0015 -> 2-0004  (1-0057 -> 1-0009  ([1061] -> [1861], 31.749695)), 2-0016 -> 2-0007  (1-0067 -> 1-0040  ([1895] -> [0477], 293.303452)),

Total Graphs For Level - 3 : 2

Level 4

=======

Graph 4-0001 : 3-0001 -> 3-0002  (2-0001 -> 2-0003  (1-0002 -> 1-0008  ([0006] -> [0036], 549.826655))), 3-0002 -> 3-0001  (2-0003 -> 2-0001  (1-0008 -> 1-0002  ([0036] -> [0006], 549.826655))),

Total Graphs For Level - 4 : 1

**Graph description of Baltimore class IV:**

Level 1

=======

Graph 1-0001 : 0018 -> 1048 (214.426340), 1048 -> 0018 (214.426340),

Graph 1-0002 : 0019 -> 0394 (149.219066), 0394 -> 0019 (149.219066), 1182 -> 0019 (226.841644),

Graph 1-0003 : 0022 -> 0893 (322.891545), 0876 -> 0893 (188.447311), 0893 -> 0876 (188.447311),

Graph 1-0004 : 0024 -> 1775 (311.805179), 1258 -> 1775 (249.459937), 1775 -> 1258 (249.459937),

Graph 1-0005 : 0037 -> 1428 (385.311063), 0318 -> 1428 (266.762313), 0450 -> 1428 (265.434219), 0891 -> 0037 (392.117593), 1428 -> 0450 (265.434219),

Graph 1-0006 : 0045 -> 0628 (315.959021), 0244 -> 0628 (257.275391), 0628 -> 0244 (257.275391), 0732 -> 0982 (463.171022), 0787 -> 0628 (269.434203), 0982 -> 0787 (278.970720), 1200 -> 0628 (369.264262), 1361 -> 0045 (355.930271),

Graph 1-0007 : 0059 -> 1363 (194.956029), 0767 -> 0059 (219.274728), 1186 -> 1363 (144.512242), 1363 -> 1186 (144.512242),

Graph 1-0008 : 0063 -> 1941 (351.997618), 1941 -> 0063 (351.997618),

Graph 1-0009 : 0066 -> 1305 (151.277736), 0139 -> 0349 (118.589511), 0349 -> 1305 (88.988838), 1076 -> 1305 (120.674501), 1268 -> 1303 (378.536172), 1303 -> 1305 (103.117596), 1305 -> 0349 (88.988838), 1370 -> 1305 (126.042049), 2009 -> 0139 (127.677163),

Graph 1-0010 : 0067 -> 0892 (121.123462), 0892 -> 0067 (121.123462),

Graph 1-0011 : 0069 -> 0070 (281.556898), 0070 -> 0388 (248.366525), 0388 -> 0070 (248.366525),

Graph 1-0012 : 0071 -> 0278 (167.746136), 0278 -> 0071 (167.746136), 2043 -> 0278 (173.102365),

Graph 1-0013 : 0076 -> 0492 (204.472963), 0350 -> 1224 (432.816961), 0456 -> 0076 (326.629251), 0492 -> 1517 (194.216618), 0613 -> 0076 (247.022975), 0650 -> 0456 (359.484247), 1224 -> 1517 (125.258748), 1330 -> 1517 (109.976829), 1338 -> 0076 (343.281167), 1517 -> 1330 (109.976829),

Graph 1-0014 : 0077 -> 1601 (62.666127), 1601 -> 0077 (62.666127),

Graph 1-0015 : 0082 -> 1462 (442.448276), 1462 -> 0082 (442.448276),

Graph 1-0016 : 0083 -> 0505 (538.107031), 0091 -> 2038 (144.765536), 0505 -> 0091 (378.444112), 0863 -> 0894 (313.493040), 0894 -> 2038 (255.119375), 0904 -> 0091 (177.096884), 2029 -> 2038 (244.319246), 2038 -> 0091 (144.765536),

Graph 1-0017 : 0084 -> 0087 (95.638169), 0087 -> 0084 (95.638169), 0140 -> 0087 (289.529807), 0488 -> 0084 (356.441301),

Graph 1-0018 : 0085 -> 1367 (137.021031), 1367 -> 0085 (137.021031),

Graph 1-0019 : 0086 -> 1034 (308.838451), 0327 -> 1034 (363.805156), 0484 -> 0749 (407.032024), 0749 -> 1034 (298.392910), 1034 -> 0749 (298.392910), 1276 -> 1034 (389.717867), 1577 -> 0327 (466.365250), 1929 -> 0484 (433.387603),

Graph 1-0020 : 0092 -> 1281 (59.402861), 0306 -> 1281 (33.009336), 0969 -> 1281 (37.452234), 1281 -> 0306 (33.009336), 1826 -> 0092 (60.106797),

Graph 1-0021 : 0093 -> 1510 (223.856555), 0215 -> 1510 (347.025154), 0216 -> 0093 (285.676503), 0959 -> 0093 (353.964720), 1510 -> 0093 (223.856555),

Graph 1-0022 : 0094 -> 0095 (46.219485), 0095 -> 0094 (46.219485),

Graph 1-0023 : 0096 -> 0754 (162.835134), 0754 -> 0096 (162.835134),

Graph 1-0024 : 0100 -> 0494 (236.542365), 0153 -> 0748 (283.037802), 0494 -> 0748 (178.244789), 0646 -> 2001 (265.371129), 0748 -> 2001 (79.078606), 1042 -> 1966 (214.174786), 1966 -> 2001 (172.012386), 2001 -> 0748 (79.078606),

Graph 1-0025 : 0106 -> 1356 (356.259785), 0788 -> 0789 (119.585117), 0789 -> 0788 (119.585117), 1033 -> 0106 (373.488272), 1356 -> 0788 (228.252220),

Graph 1-0026 : 0136 -> 0984 (84.946049), 0984 -> 0136 (84.946049), 1099 -> 0136 (207.862084),

Graph 1-0027 : 0138 -> 0769 (277.682958), 0769 -> 0138 (277.682958),

Graph 1-0028 : 0149 -> 0152 (67.108480), 0150 -> 0152 (465.587307), 0151 -> 0152 (99.406699), 0152 -> 0149 (67.108480),

Graph 1-0029 : 0154 -> 1183 (114.271983), 0447 -> 1622 (164.275846), 1183 -> 1622 (110.100212), 1622 -> 1183 (110.100212),

Graph 1-0030 : 0156 -> 0161 (106.597956), 0161 -> 0156 (106.597956),

Graph 1-0031 : 0157 -> 0158 (228.409419), 0158 -> 0157 (228.409419), 0248 -> 0157 (587.662190),

Graph 1-0032 : 0159 -> 0164 (1273.676689), 0162 -> 0159 (1557.063265), 0163 -> 0164 (785.749953), 0164 -> 0163 (785.749953),

Graph 1-0033 : 0160 -> 0783 (733.270045), 0783 -> 0160 (733.270045), 0785 -> 0783 (1584.466637),

Graph 1-0034 : 0167 -> 1600 (126.600179), 0652 -> 1600 (244.793218), 0655 -> 0167 (128.137494), 1600 -> 0167 (126.600179),

Graph 1-0035 : 0168 -> 1274 (124.667769), 1274 -> 0168 (124.667769),

Graph 1-0036 : 0169 -> 1598 (155.762460), 0341 -> 0169 (174.225089), 1598 -> 0169 (155.762460),

Graph 1-0037 : 0171 -> 0389 (114.439730), 0389 -> 0171 (114.439730),

Graph 1-0038 : 0173 -> 1418 (135.566854), 0511 -> 1418 (147.197022), 0540 -> 1417 (112.384624), 1417 -> 0540 (112.384624), 1418 -> 0540 (118.488055),

Graph 1-0039 : 0174 -> 0310 (138.762428), 0178 -> 0310 (105.312646), 0310 -> 0178 (105.312646), 2007 -> 0174 (528.051356),

Graph 1-0040 : 0179 -> 2042 (119.778694), 0460 -> 2042 (166.481212), 2003 -> 2042 (128.439025), 2042 -> 0179 (119.778694),

Graph 1-0041 : 0181 -> 0309 (105.858684), 0309 -> 0181 (105.858684), 1486 -> 1487 (426.411575), 1487 -> 0181 (356.745076),

Graph 1-0042 : 0182 -> 1046 (97.520762), 0448 -> 1046 (85.110707), 1046 -> 0448 (85.110707), 1956 -> 0448 (113.165906),

Graph 1-0043 : 0183 -> 1063 (263.421225), 0981 -> 0183 (1063.521878), 1063 -> 0183 (263.421225),

Graph 1-0044 : 0187 -> 1813 (56.872973), 0251 -> 0187 (83.134659), 1300 -> 0187 (80.681861), 1454 -> 1813 (51.552807), 1689 -> 1876 (191.544217), 1812 -> 1300 (98.186966), 1813 -> 1454 (51.552807), 1876 -> 1300 (94.706978),

Graph 1-0045 : 0193 -> 1967 (147.156117), 1248 -> 1967 (155.365359), 1967 -> 0193 (147.156117),

Graph 1-0046 : 0196 -> 0335 (284.580490), 0333 -> 0335 (178.147415), 0335 -> 0333 (178.147415), 0491 -> 0333 (312.449010), 0667 -> 0333 (182.868733), 1739 -> 0335 (337.164364),

Graph 1-0047 : 0198 -> 1760 (275.942625), 0895 -> 1760 (756.301234), 1589 -> 1767 (765.707730), 1760 -> 0198 (275.942625), 1767 -> 1760 (279.949860),

Graph 1-0048 : 0202 -> 0750 (185.570158), 0745 -> 0202 (188.452778), 0750 -> 1269 (163.196465), 0857 -> 1269 (147.734712), 0963 -> 0857 (191.443516), 0965 -> 0963 (264.161165), 1269 -> 0857 (147.734712), 1365 -> 1269 (211.239464),

Graph 1-0049 : 0208 -> 0247 (175.862213), 0246 -> 0208 (183.836822), 0247 -> 0208 (175.862213), 0378 -> 0247 (197.459772),

Graph 1-0050 : 0214 -> 1791 (145.506796), 1791 -> 0214 (145.506796),

Graph 1-0051 : 0222 -> 0241 (67.832940), 0241 -> 0791 (37.874568), 0242 -> 0791 (35.494774), 0596 -> 0241 (187.963312), 0784 -> 1351 (1894.301287), 0786 -> 0242 (259.024084), 0791 -> 0242 (35.494774), 1351 -> 0786 (352.298178),

Graph 1-0052 : 0223 -> 0796 (189.649526), 0794 -> 0796 (96.243353), 0796 -> 0794 (96.243353),

Graph 1-0053 : 0230 -> 1352 (313.354772), 1352 -> 0230 (313.354772),

Graph 1-0054 : 0245 -> 1311 (199.513519), 1311 -> 0245 (199.513519),

Graph 1-0055 : 0249 -> 0888 (650.271391), 0466 -> 0888 (41.089233), 0888 -> 0466 (41.089233), 1590 -> 0249 (722.782876),

Graph 1-0056 : 0250 -> 0964 (220.890172), 0964 -> 0250 (220.890172), 1483 -> 0964 (257.796127),

Graph 1-0057 : 0275 -> 0889 (232.228311), 0289 -> 0889 (327.179521), 0729 -> 0889 (163.082042), 0889 -> 0729 (163.082042),

Graph 1-0058 : 0277 -> 0445 (230.415743), 0441 -> 2041 (217.395766), 0442 -> 2041 (158.549687), 0445 -> 2041 (125.424255), 0906 -> 2041 (104.337618), 1519 -> 0445 (354.364201), 2041 -> 0906 (104.337618),

Graph 1-0059 : 0279 -> 1193 (37.239253), 0280 -> 0279 (53.442964), 0741 -> 1094 (509.036179), 1094 -> 0279 (162.150675), 1193 -> 0279 (37.239253),

Graph 1-0060 : 0281 -> 1201 (330.720045), 1201 -> 0281 (330.720045),

Graph 1-0061 : 0299 -> 0421 (175.708234), 0421 -> 1821 (159.850493), 0905 -> 1801 (185.695793), 1509 -> 0421 (186.812893), 1801 -> 0421 (160.383402), 1818 -> 1821 (129.433808), 1821 -> 1818 (129.433808),

Graph 1-0062 : 0302 -> 1949 (57.975857), 0707 -> 1949 (177.475033), 1949 -> 0302 (57.975857),

Graph 1-0063 : 0307 -> 1953 (140.047685), 1953 -> 0307 (140.047685),

Graph 1-0064 : 0308 -> 1484 (103.209623), 0390 -> 0308 (165.836050), 1016 -> 1272 (160.418827), 1272 -> 1484 (152.178397), 1484 -> 0308 (103.209623), 1797 -> 1484 (122.431388),

Graph 1-0065 : 0311 -> 0696 (1042.198842), 0375 -> 0695 (1759.250137), 0694 -> 0696 (172.538640), 0695 -> 1685 (1362.877152), 0696 -> 0694 (172.538640), 0980 -> 1685 (1145.142055), 1447 -> 0695 (1692.980739), 1685 -> 0694 (1112.658223),

Graph 1-0066 : 0313 -> 1759 (412.343011), 0426 -> 1759 (462.056214), 1318 -> 1759 (305.487839), 1759 -> 1318 (305.487839),

Graph 1-0067 : 0314 -> 1369 (210.332661), 1369 -> 0314 (210.332661),

Graph 1-0068 : 0322 -> 0323 (121.108178), 0323 -> 0322 (121.108178), 0621 -> 1471 (335.089738), 1247 -> 0322 (484.345604), 1271 -> 0323 (141.355690), 1471 -> 1271 (251.137021), 1513 -> 1271 (363.814837), 1514 -> 1513 (435.600535),

Graph 1-0069 : 0329 -> 0499 (240.759387), 0499 -> 0897 (178.534638), 0897 -> 0499 (178.534638), 1214 -> 0329 (368.886850), 1324 -> 0897 (519.630000), 1955 -> 0897 (216.699104),

Graph 1-0070 : 0332 -> 1184 (242.695517), 1184 -> 0332 (242.695517),

Graph 1-0071 : 0334 -> 1277 (151.866715), 1277 -> 0334 (151.866715),

Graph 1-0072 : 0336 -> 0846 (301.154504), 0846 -> 0850 (232.788156), 0850 -> 0851 (201.856544), 0851 -> 0850 (201.856544), 0852 -> 0850 (251.096948), 1360 -> 0852 (290.732361),

Graph 1-0073 : 0338 -> 0778 (200.709937), 0778 -> 0338 (200.709937), 1946 -> 0338 (331.685381),

Graph 1-0074 : 0345 -> 1203 (230.099892), 1203 -> 0345 (230.099892),

Graph 1-0075 : 0360 -> 0461 (259.307426), 0461 -> 1342 (136.614301), 1342 -> 0461 (136.614301),

Graph 1-0076 : 0373 -> 1485 (409.934022), 1319 -> 1453 (176.039407), 1453 -> 1319 (176.039407), 1482 -> 1453 (279.710143), 1485 -> 1482 (378.983433),

Graph 1-0077 : 0374 -> 0697 (420.708391), 0692 -> 1220 (1167.962668), 0697 -> 1013 (309.843864), 1013 -> 0697 (309.843864), 1204 -> 0697 (426.804007), 1220 -> 0374 (457.681038),

Graph 1-0078 : 0391 -> 0880 (137.827148), 0422 -> 0880 (73.556160), 0746 -> 1480 (119.410019), 0880 -> 0422 (73.556160), 1480 -> 0880 (115.914345), 1493 -> 0746 (126.422999),

Graph 1-0079 : 0392 -> 1333 (264.831909), 0757 -> 0392 (465.825981), 1333 -> 1736 (149.014979), 1736 -> 1333 (149.014979),

Graph 1-0080 : 0399 -> 1448 (1171.210589), 1092 -> 1448 (214.381288), 1093 -> 1092 (265.226617), 1448 -> 1092 (214.381288),

Graph 1-0081 : 0433 -> 2036 (59.601942), 1292 -> 2036 (154.982702), 1463 -> 2036 (27.817995), 1954 -> 1463 (42.506760), 2036 -> 1463 (27.817995),

Graph 1-0082 : 0438 -> 1225 (146.915889), 1225 -> 0438 (146.915889),

Graph 1-0083 : 0440 -> 0446 (162.211887), 0446 -> 1286 (68.958834), 0458 -> 0690 (88.561824), 0690 -> 1286 (39.966667), 1286 -> 0690 (39.966667), 1337 -> 0446 (236.797081),

Graph 1-0084 : 0443 -> 1371 (111.219282), 1371 -> 0443 (111.219282),

Graph 1-0085 : 0462 -> 2037 (185.119824), 2037 -> 0462 (185.119824),

Graph 1-0086 : 0469 -> 0471 (100.417850), 0470 -> 0469 (158.139289), 0471 -> 0469 (100.417850), 0472 -> 0471 (184.900770),

Graph 1-0087 : 0474 -> 1525 (383.456957), 0519 -> 1594 (142.281307), 0581 -> 1593 (138.218901), 1205 -> 1593 (140.526056), 1525 -> 1593 (62.686287), 1593 -> 1525 (62.686287), 1594 -> 1593 (108.321262),

Graph 1-0088 : 0475 -> 1188 (72.331278), 1188 -> 0475 (72.331278),

Graph 1-0089 : 0493 -> 1997 (146.694972), 0953 -> 0493 (221.237225), 1470 -> 0493 (255.218561), 1756 -> 0493 (205.285867), 1997 -> 0493 (146.694972),

Graph 1-0090 : 0498 -> 1307 (132.078318), 0867 -> 1307 (429.617968), 1307 -> 0498 (132.078318),

Graph 1-0091 : 0504 -> 1943 (419.997394), 0601 -> 1943 (238.251381), 1784 -> 0504 (471.248377), 1942 -> 1943 (208.640023), 1943 -> 1942 (208.640023),

Graph 1-0092 : 0595 -> 1465 (1304.316284), 1007 -> 1465 (718.190264), 1198 -> 1007 (954.812431), 1465 -> 1007 (718.190264),

Graph 1-0093 : 0602 -> 0603 (174.988542), 0603 -> 0602 (174.988542), 1511 -> 0602 (313.848550),

Graph 1-0094 : 0624 -> 1429 (133.252456), 1429 -> 0624 (133.252456),

Graph 1-0095 : 0639 -> 0644 (64.114349), 0640 -> 0639 (85.663998), 0641 -> 0639 (91.045341), 0642 -> 0640 (102.334594), 0644 -> 0639 (64.114349),

Graph 1-0096 : 0643 -> 0645 (54.149031), 0645 -> 0643 (54.149031),

Graph 1-0097 : 0661 -> 1816 (143.627960), 0952 -> 1816 (187.317623), 1219 -> 1221 (69.303800), 1221 -> 1219 (69.303800), 1815 -> 1221 (83.707426), 1816 -> 1219 (117.828831),

Graph 1-0098 : 0668 -> 1526 (225.176687), 1526 -> 0668 (225.176687),

Graph 1-0099 : 0669 -> 0670 (227.550673), 0670 -> 0669 (227.550673),

Graph 1-0100 : 0671 -> 1970 (313.301923), 1433 -> 1970 (192.830660), 1682 -> 1970 (79.315743), 1970 -> 1682 (79.315743), 1996 -> 1970 (90.991218),

Graph 1-0101 : 0672 -> 0673 (249.644876), 0673 -> 0672 (249.644876),

Graph 1-0102 : 0676 -> 1475 (256.720768), 1475 -> 0676 (256.720768), 1520 -> 0676 (297.985674), 1572 -> 1475 (264.544970),

Graph 1-0103 : 0679 -> 2006 (1825.612365), 2006 -> 0679 (1825.612365),

Graph 1-0104 : 0693 -> 1332 (800.756202), 0907 -> 0693 (1034.622802), 1327 -> 0693 (933.539293), 1332 -> 0693 (800.756202),

Graph 1-0105 : 0698 -> 0793 (358.647447), 0792 -> 0795 (34.842112), 0793 -> 0792 (68.933738), 0795 -> 0792 (34.842112), 0797 -> 0792 (72.008946),

Graph 1-0106 : 0699 -> 0700 (150.307442), 0700 -> 0699 (150.307442), 1945 -> 0700 (347.004502),

Graph 1-0107 : 0701 -> 1226 (168.904362), 1226 -> 0701 (168.904362),

Graph 1-0108 : 0734 -> 0739 (166.750654), 0738 -> 0734 (222.766352), 0739 -> 0734 (166.750654), 1479 -> 0739 (1220.698880),

Graph 1-0109 : 0735 -> 0736 (193.629788), 0736 -> 0737 (112.930927), 0737 -> 0736 (112.930927), 0742 -> 0735 (536.992908),

Graph 1-0110 : 0744 -> 1467 (194.727324), 0864 -> 1467 (149.732921), 0885 -> 1467 (159.548057), 1467 -> 0864 (149.732921),

Graph 1-0111 : 0747 -> 1208 (169.827595), 0790 -> 0747 (308.756470), 1208 -> 1262 (89.412366), 1209 -> 1262 (186.648245), 1262 -> 1208 (89.412366), 1293 -> 0790 (410.820045),

Graph 1-0112 : 0765 -> 0766 (152.248023), 0766 -> 0765 (152.248023), 1364 -> 0766 (153.516012), 1366 -> 0766 (170.917989),

Graph 1-0113 : 0798 -> 1553 (106.406161), 1339 -> 0798 (147.301437), 1349 -> 1553 (91.656633), 1553 -> 1349 (91.656633), 1554 -> 1349 (121.645636), 1555 -> 1349 (99.302384), 1561 -> 1349 (95.100117),

Graph 1-0114 : 0799 -> 1563 (176.355294), 1552 -> 1563 (213.651318), 1562 -> 1563 (70.291705), 1563 -> 1562 (70.291705),

Graph 1-0115 : 0813 -> 1495 (38.021346), 1495 -> 0813 (38.021346),

Graph 1-0116 : 0856 -> 1492 (213.556409), 1492 -> 0856 (213.556409),

Graph 1-0117 : 0858 -> 1937 (279.714501), 1329 -> 1937 (230.172772), 1937 -> 1329 (230.172772),

Graph 1-0118 : 0865 -> 0983 (409.664318), 0983 -> 0865 (409.664318),

Graph 1-0119 : 0866 -> 1947 (168.639152), 1947 -> 0866 (168.639152),

Graph 1-0120 : 0879 -> 1999 (111.706184), 1962 -> 2000 (143.458844), 1999 -> 0879 (111.706184), 2000 -> 1999 (122.251157),

Graph 1-0121 : 0881 -> 0886 (163.556117), 0886 -> 0881 (163.556117),

Graph 1-0122 : 0951 -> 0985 (144.995847), 0985 -> 0951 (144.995847), 1794 -> 0951 (249.981352),

Graph 1-0123 : 0957 -> 1952 (321.752438), 1952 -> 0957 (321.752438),

Graph 1-0124 : 0966 -> 1316 (232.714959), 1064 -> 0966 (410.699120), 1316 -> 1740 (213.527660), 1362 -> 1820 (159.831356), 1376 -> 1362 (323.339253), 1687 -> 0966 (310.232757), 1740 -> 1362 (166.989284), 1820 -> 1362 (159.831356),

Graph 1-0125 : 0987 -> 1729 (90.513063), 1729 -> 0987 (90.513063),

Graph 1-0126 : 1008 -> 1250 (152.746850), 1036 -> 1008 (169.296876), 1250 -> 1008 (152.746850),

Graph 1-0127 : 1009 -> 1291 (205.291366), 1291 -> 1592 (127.818090), 1592 -> 1291 (127.818090), 1734 -> 1009 (224.327205),

Graph 1-0128 : 1012 -> 1197 (131.281682), 1197 -> 1012 (131.281682),

Graph 1-0129 : 1035 -> 1331 (260.254279), 1331 -> 1035 (260.254279),

Graph 1-0130 : 1062 -> 1242 (305.512522), 1242 -> 1062 (305.512522),

Graph 1-0131 : 1068 -> 1074 (243.470333), 1074 -> 1068 (243.470333),

Graph 1-0132 : 1086 -> 1792 (1371.705671), 1792 -> 1086 (1371.705671),

Graph 1-0133 : 1104 -> 1275 (111.502674), 1275 -> 1104 (111.502674),

Graph 1-0134 : 1185 -> 1588 (336.893204), 1588 -> 1185 (336.893204),

Graph 1-0135 : 1192 -> 1229 (130.410158), 1229 -> 1192 (130.410158), 1259 -> 1512 (232.787572), 1512 -> 1192 (218.842638), 2005 -> 1229 (218.249960),

Graph 1-0136 : 1207 -> 2004 (128.954652), 2004 -> 1207 (128.954652),

Graph 1-0137 : 1222 -> 1372 (177.584475), 1372 -> 1222 (177.584475),

Graph 1-0138 : 1223 -> 1527 (225.119596), 1527 -> 1223 (225.119596),

Graph 1-0139 : 1227 -> 1228 (170.014362), 1228 -> 1227 (170.014362),

Graph 1-0140 : 1260 -> 1936 (233.892885), 1780 -> 1936 (454.404747), 1936 -> 1260 (233.892885),

Graph 1-0141 : 1279 -> 1793 (107.485344), 1793 -> 1279 (107.485344),

Graph 1-0142 : 1283 -> 1285 (64.319068), 1284 -> 1283 (76.101939), 1285 -> 1283 (64.319068),

Graph 1-0143 : 1301 -> 1781 (139.028575), 1781 -> 1301 (139.028575), 2010 -> 1781 (226.283968), 2028 -> 1301 (148.479406),

Graph 1-0144 : 1302 -> 1309 (68.512738), 1309 -> 1302 (68.512738),

Graph 1-0145 : 1348 -> 1507 (242.277861), 1505 -> 1507 (59.144530), 1507 -> 1505 (59.144530), 1522 -> 1507 (294.618268),

Graph 1-0146 : 1350 -> 1516 (676.868882), 1516 -> 1350 (676.868882),

Graph 1-0147 : 1354 -> 1557 (795.238637), 1557 -> 1354 (795.238637),

Graph 1-0148 : 1357 -> 1358 (88.972557), 1358 -> 1357 (88.972557),

Graph 1-0149 : 1368 -> 2008 (131.914964), 2008 -> 1368 (131.914964),

Graph 1-0150 : 1431 -> 1506 (73.216202), 1506 -> 1431 (73.216202),

Graph 1-0151 : 1496 -> 1576 (59.915819), 1576 -> 1496 (59.915819),

Graph 1-0152 : 1524 -> 1998 (137.303793), 1998 -> 1524 (137.303793), 2035 -> 1524 (267.893854),

Total Graphs For Level - 1 : 152

Level 2

=======

Graph 2-0001 : 1-0001 -> 1-0087 ([1048] -> [0581], 238.784061), 1-0014 -> 1-0078 ([1601] -> [0422], 74.352385), 1-0038 -> 1-0097 ([0173] -> [1219], 168.264066), 1-0062 -> 1-0078 ([0302] -> [0422], 110.311269), 1-0063 -> 1-0062 ([1953] -> [0302], 142.784874), 1-0078 -> 1-0014 ([0422] -> [1601], 74.352385), 1-0087 -> 1-0078 ([1205] -> [0880], 142.941640), 1-0097 -> 1-0142 ([0661] -> [1285], 154.039118), 1-0125 -> 1-0087 ([0987] -> [0519], 163.583666), 1-0126 -> 1-0125 ([1250] -> [1729], 188.289857), 1-0128 -> 1-0062 ([1012] -> [1949], 141.393556), 1-0133 -> 1-0087 ([1275] -> [1593], 168.814628), 1-0142 -> 1-0078 ([1284] -> [0746], 125.653940),

Graph 2-0002 : 1-0002 -> 1-0057 ([0019] -> [0889], 210.145383), 1-0046 -> 1-0057 ([0667] -> [0729], 267.839588), 1-0048 -> 1-0112 ([0750] -> [0766], 181.394476), 1-0057 -> 1-0048 ([0889] -> [0963], 205.107441), 1-0093 -> 1-0002 ([0602] -> [1182], 508.226551), 1-0112 -> 1-0048 ([0766] -> [0750], 181.394476),

Graph 2-0003 : 1-0003 -> 1-0009 ([0876] -> [2009], 448.691918), 1-0009 -> 1-0149 ([1303] -> [1368], 135.352969), 1-0029 -> 1-0009 ([0154] -> [0066], 157.822618), 1-0045 -> 1-0009 ([1967] -> [1305], 155.817458), 1-0079 -> 1-0009 ([1333] -> [1305], 192.649080), 1-0121 -> 1-0045 ([0881] -> [1967], 224.538358), 1-0143 -> 1-0009 ([1781] -> [2009], 140.540264), 1-0144 -> 1-0149 ([1302] -> [2008], 156.006690), 1-0149 -> 1-0009 ([1368] -> [1303], 135.352969),

Graph 2-0004 : 1-0004 -> 1-0089 ([1258] -> [1997], 275.043752), 1-0035 -> 1-0089 ([0168] -> [0493], 205.681816), 1-0085 -> 1-0089 ([0462] -> [0493], 258.762340), 1-0089 -> 1-0035 ([0493] -> [0168], 205.681816), 1-0138 -> 1-0004 ([1527] -> [1258], 346.380337), 1-0140 -> 1-0089 ([1260] -> [0953], 255.007602),

Graph 2-0005 : 1-0005 -> 1-0137 ([0450] -> [1372], 360.952778), 1-0010 -> 1-0137 ([0067] -> [1222], 221.485104), 1-0122 -> 1-0137 ([0985] -> [1372], 181.810113), 1-0137 -> 1-0122 ([1372] -> [0985], 181.810113),

Graph 2-0006 : 1-0006 -> 1-0091 ([0787] -> [1943], 282.539018), 1-0053 -> 1-0091 ([1352] -> [1784], 484.505282), 1-0091 -> 1-0006 ([1943] -> [0787], 282.539018), 1-0116 -> 1-0006 ([0856] -> [0787], 422.019996),

Graph 2-0007 : 1-0007 -> 1-0061 ([1363] -> [0299], 182.118976), 1-0034 -> 1-0061 ([0167] -> [1818], 142.268250), 1-0037 -> 1-0034 ([0171] -> [0167], 157.590064), 1-0040 -> 1-0061 ([2042] -> [1818], 149.497297), 1-0056 -> 1-0007 ([1483] -> [0059], 260.512594), 1-0061 -> 1-0034 ([1818] -> [0167], 142.268250), 1-0123 -> 1-0007 ([1952] -> [1186], 322.065642), 1-0127 -> 1-0061 ([1009] ->

[0299], 224.794830), 1-0136 -> 1-0061 ([2004] -> [1509], 247.808918), 1-0141 -> 1-0034 ([1279] -> [0655], 149.804365),

Graph 2-0008 : 1-0008 -> 1-0115 ([1941] -> [1495], 382.197746), 1-0115 -> 1-0008 ([1495] -> [1941], 382.197746),

Graph 2-0009 : 1-0011 -> 1-0021 ([0069] -> [1510], 316.975022), 1-0021 -> 1-0011 ([1510] -> [0069], 316.975022), 1-0118 -> 1-0021 ([0865] -> [0959], 470.302970),

Graph 2-0010 : 1-0012 -> 1-0135 ([0278] -> [1229], 178.150384), 1-0027 -> 1-0135 ([0769] -> [1229], 291.447844), 1-0067 -> 1-0135 ([1369] -> [1259], 314.735888), 1-0135 -> 1-0012 ([1229] -> [0278], 178.150384),

Graph 2-0011 : 1-0013 -> 1-0069 ([1224] -> [0499], 363.906646), 1-0069 -> 1-0088 ([0499] -> [1188], 301.600154), 1-0077 -> 1-0088 ([0697] -> [0475], 319.031926), 1-0088 -> 1-0069 ([1188] -> [0499], 301.600154), 1-0117 -> 1-0069 ([1329] -> [1955], 420.391945),

Graph 2-0012 : 1-0015 -> 1-0055 ([1462] -> [0466], 464.914668), 1-0055 -> 1-0015 ([0466] -> [1462], 464.914668),

Graph 2-0013 : 1-0016 -> 1-0026 ([0904] -> [0136], 182.606284), 1-0026 -> 1-0016 ([0136] -> [0904], 182.606284), 1-0086 -> 1-0152 ([0472] -> [1524], 338.927093), 1-0120 -> 1-0026 ([0879] -> [0984], 232.052806), 1-0152 -> 1-0016 ([1998] -> [2029], 270.228581),

Graph 2-0014 : 1-0017 -> 1-0106 ([0084] -> [0700], 260.903816), 1-0106 -> 1-0017 ([0700] -> [0084], 260.903816),

Graph 2-0015 : 1-0018 -> 1-0111 ([0085] -> [1262], 163.385513), 1-0023 -> 1-0018 ([0096] -> [1367], 215.411803), 1-0111 -> 1-0018 ([1262] -> [0085], 163.385513),

Graph 2-0016 : 1-0019 -> 1-0090 ([0086] -> [0498], 317.953858), 1-0090 -> 1-0019 ([0498] -> [0086], 317.953858), 1-0129 -> 1-0019 ([1035] -> [0749], 456.429872),

Graph 2-0017 : 1-0020 -> 1-0083 ([0306] -> [1286], 54.793479), 1-0083 -> 1-0020 ([1286] -> [0306], 54.793479), 1-0084 -> 1-0083 ([0443] -> [0440], 196.836494),

Graph 2-0018 : 1-0022 -> 1-0044 ([0095] -> [1812], 279.358936), 1-0044 -> 1-0081 ([0187] -> [1954], 113.696910), 1-0058 -> 1-0044 ([0277] -> [1454], 282.695059), 1-0081 -> 1-0044 ([1954] -> [0187], 113.696910),

Graph 2-0019 : 1-0024 -> 1-0102 ([1966] -> [1475], 307.068766), 1-0074 -> 1-0024 ([1203] -> [0100], 311.241994), 1-0102 -> 1-0024 ([1475] -> [1966], 307.068766), 1-0151 -> 1-0102 ([1576] -> [1520], 664.002408),

Graph 2-0020 : 1-0025 -> 1-0072 ([0789] -> [0852], 266.588525), 1-0070 -> 1-0072 ([0332] -> [1360], 300.458776), 1-0072 -> 1-0025 ([0852] -> [0789], 266.588525), 1-0073 -> 1-0070 ([0338] -> [0332], 304.899245),

Graph 2-0021 : 1-0028 -> 1-0039 ([0151] -> [0178], 143.768493), 1-0036 -> 1-0039 ([0341] -> [0174], 207.150970), 1-0039 -> 1-0042 ([0178] -> [1956], 136.055295), 1-0042 -> 1-0039 ([1956] -> [0178], 136.055295), 1-0068 -> 1-0042 ([0322] -> [0182], 187.342976), 1-0124 -> 1-0036 ([1820] -> [0341], 231.163825),

Graph 2-0022 : 1-0030 -> 1-0051 ([0161] -> [1351], 859.362527), 1-0051 -> 1-0030 ([1351] -> [0161], 859.362527), 1-0080 -> 1-0051 ([0399] -> [0222], 1432.552125),

Graph 2-0023 : 1-0031 -> 1-0146 ([0158] -> [1516], 699.918647), 1-0032 -> 1-0146 ([0163] -> [1350], 1066.088312), 1-0146 -> 1-0031 ([1516] -> [0158], 699.918647),

Graph 2-0024 : 1-0033 -> 1-0119 ([0783] -> [0866], 830.937650), 1-0103 -> 1-0132 ([0679] -> [1086], 2583.400259), 1-0119 -> 1-0033 ([0866] -> [0783], 830.937650), 1-0132 -> 1-0033 ([1792] -> [0783], 1481.161040),

Graph 2-0025 : 1-0041 -> 1-0082 ([0181] -> [0438], 415.593033), 1-0050 -> 1-0082 ([0214] -> [0438], 159.772616), 1-0082 -> 1-0050 ([0438] -> [0214], 159.772616),

Graph 2-0026 : 1-0043 -> 1-0147 ([1063] -> [1557], 1083.300956), 1-0065 -> 1-0043 ([0311] -> [0183], 1197.073451), 1-0147 -> 1-0043 ([1557] -> [1063], 1083.300956),

Graph 2-0027 : 1-0047 -> 1-0131 ([1767] -> [1074], 313.510983), 1-0131 -> 1-0047 ([1074] -> [1767], 313.510983),

Graph 2-0028 : 1-0049 -> 1-0054 ([0208] -> [0245], 317.327034), 1-0054 -> 1-0049 ([0245] -> [0208], 317.327034), 1-0092 -> 1-0049 ([1465] -> [0378], 718.957105), 1-0104 -> 1-0054 ([1327] -> [0245], 1042.322845),

Graph 2-0029 : 1-0052 -> 1-0105 ([0794] -> [0795], 108.576639), 1-0105 -> 1-0052 ([0795] -> [0794], 108.576639),

Graph 2-0030 : 1-0059 -> 1-0145 ([1094] -> [1348], 354.133474), 1-0094 -> 1-0150 ([0624] -> [1431], 143.725784), 1-0145 -> 1-0150 ([1507] -> [1506], 133.882451), 1-0150 -> 1-0145 ([1506] -> [1507], 133.882451),

Graph 2-0031 : 1-0060 -> 1-0130 ([0281] -> [1062], 373.030929), 1-0130 -> 1-0060 ([1062] -> [0281], 373.030929),

Graph 2-0032 : 1-0064 -> 1-0110 ([1016] -> [0885], 169.726619), 1-0110 -> 1-0064 ([0885] -> [1016], 169.726619),

Graph 2-0033 : 1-0066 -> 1-0075 ([1318] -> [0360], 330.324486), 1-0075 -> 1-0076 ([0461] -> [1319], 232.271230), 1-0076 -> 1-0075 ([1319] -> [0461], 232.271230),

Graph 2-0034 : 1-0071 -> 1-0134 ([0334] -> [1588], 356.925082), 1-0134 -> 1-0071 ([1588] -> [0334], 356.925082),

Graph 2-0035 : 1-0095 -> 1-0096 ([0639] -> [0645], 65.952374), 1-0096 -> 1-0095 ([0645] -> [0639], 65.952374),

Graph 2-0036 : 1-0098 -> 1-0099 ([0668] -> [0670], 343.333323), 1-0099 -> 1-0100 ([0669] -> [1970], 259.522350), 1-0100 -> 1-0099 ([1970] -> [0669], 259.522350),

Graph 2-0037 : 1-0101 -> 1-0109 ([0673] -> [0737], 527.954604), 1-0108 -> 1-0109 ([0738] -> [0735], 250.062074), 1-0109 -> 1-0108 ([0735] -> [0738], 250.062074),

Graph 2-0038 : 1-0107 -> 1-0139 ([0701] -> [1227], 173.501062), 1-0139 -> 1-0107 ([1227] -> [0701], 173.501062),

Graph 2-0039 : 1-0113 -> 1-0148 ([1349] -> [1357], 120.743713), 1-0114 -> 1-0113 ([1562] -> [1561], 147.801750), 1-0148 -> 1-0113 ([1357] -> [1349], 120.743713),

Total Graphs For Level - 2 : 39

Level 3

=======

Graph 3-0001 : 2-0001 -> 2-0032 (1-0087 -> 1-0064 ([0519] -> [0308], 169.876878)), 2-0017 -> 2-0001 (1-0084 -> 1-0125 ([1371] -> [0987], 203.573511)), 2-0032 -> 2-0001 (1-0064 -> 1-0087 ([0308] -> [0519], 169.876878)),

Graph 3-0002 : 2-0002 -> 2-0033 (1-0002 -> 1-0075 ([0394] -> [1342], 267.728560)), 2-0033 -> 2-0002 (1-0075 -> 1-0002 ([1342] -> [0394], 267.728560)), 2-0034 -> 2-0002 (1-0134 -> 1-0046 ([1588] -> [1739], 363.938250)), 2-0035 -> 2-0036 (1-0096 -> 1-0100 ([0643] -> [1682], 468.277781)), 2-0036 -> 2-0002 (1-0100 -> 1-0048 ([1682] -> [0965], 348.635480)), 2-0037 -> 2-0002 (1-0109 -> 1-0093 ([0742] -> [1511], 548.476211)),

Graph 3-0003 : 2-0003 -> 2-0007 (1-0143 -> 1-0034 ([2028] -> [1600], 151.142462)), 2-0004 -> 2-0003 (1-0035 -> 1-0029 ([1274] -> [1622], 206.788487)), 2-0007 -> 2-0003 (1-0034 -> 1-0143 ([1600] -> [2028], 151.142462)), 2-0012 -> 2-0004 (1-0015 -> 1-0004 ([1462] -> [0024], 468.810029)), 2-0016 -> 2-0007 (1-0090 -> 1-0040 ([1307] -> [2003], 365.825987)),

Graph 3-0004 : 2-0005 -> 2-0013 (1-0137 -> 1-0016 ([1222] -> [0894], 324.480835)), 2-0013 -> 2-0005 (1-0016 -> 1-0137 ([0894] -> [1222], 324.480835)), 2-0019 -> 2-0005 (1-0024 -> 1-0005 ([0646] -> [0891], 530.579509)), 2-0026 -> 2-0028 (1-0043 -> 1-0104 ([0981] -> [1332], 1113.208360)), 2-0027 -> 2-0013 (1-0131 -> 1-0152 ([1068] -> [1524], 396.847147)), 2-0028 -> 2-0019 (1-0049 -> 1-0074 ([0378] -> [1203], 749.041720)),

Graph 3-0005 : 2-0006 -> 2-0029 (1-0006 -> 1-0052 ([0244] -> [0796], 296.222788)), 2-0008 -> 2-0006 (1-0115 -> 1-0053 ([0813] -> [1352], 508.948699)), 2-0009 -> 2-0010 (1-0021 -> 1-0012 ([0215] -> [2043], 387.341929)), 2-0010 -> 2-0020 (1-0135 -> 1-0070 ([1512] -> [0332], 351.477827)), 2-0011 -> 2-0010 (1-0013 -> 1-0027 ([0650] -> [0138], 364.799146)), 2-0014 -> 2-0020 (1-0017 -> 1-0072 ([0087] -> [0336], 340.282425)), 2-0020 -> 2-0039 (1-0072 -> 1-0114 ([0846] -> [0799], 276.568164)), 2-0021 -> 2-0011 (1-0124 -> 1-0013 ([1362] -> [0650], 398.333896)), 2-0029 -> 2-0039 (1-0105 -> 1-0113 ([0797] -> [1349], 153.046530)), 2-0030 -> 2-0029 (1-0094 -> 1-0105 ([1429] -> [0797], 207.051459)), 2-0031 -> 2-0010 (1-0060 -> 1-0012 ([1201] -> [0071], 384.666930)), 2-0039 -> 2-0029 (1-0113 -> 1-0105 ([1349] -> [0797], 153.046530)),

Graph 3-0006 : 2-0015 -> 2-0018 (1-0111 -> 1-0044 ([1208] -> [1812], 175.245128)), 2-0018 -> 2-0015 (1-0044 -> 1-0111 ([1812] -> [1208], 175.245128)),

Graph 3-0007 : 2-0022 -> 2-0023 (1-0030 -> 1-0032 ([0156] -> [0159], 1662.121483)), 2-0023 -> 2-0024 (1-0146 -> 1-0033 ([1516] -> [0783], 1079.100882)), 2-0024 -> 2-0023 (1-0033 -> 1-0146 ([0783] -> [1516], 1079.100882)),

Graph 3-0008 : 2-0025 -> 2-0038 (1-0082 -> 1-0107 ([1225] -> [0701], 234.910385)), 2-0038 -> 2-0025 (1-0107 -> 1-0082 ([0701] -> [1225], 234.910385)),

Total Graphs For Level - 3 : 8

Level 4

=======

Graph 4-0001 : 3-0001 -> 3-0005 (2-0017 -> 2-0021 (1-0020 -> 1-0028 ([1826] -> [0150], 652.512188))), 3-0005 -> 3-0006 (2-0020 -> 2-0015 (1-0073 -> 1-0023 ([0778] -> [0096], 384.875279))), 3-0006 -> 3-0005 (2-0015 -> 2-0020 (1-0023 -> 1-0073 ([0096] -> [0778], 384.875279))), 3-0008 -> 3-0001 (2-0025 -> 2-0001 (1-0041 -> 1-0038 ([1487] -> [0511], 735.084795))),

Graph 4-0002 : 3-0002 -> 3-0003 (2-0033 -> 2-0016 (1-0076 -> 1-0019 ([1482] -> [1276], 418.268398))), 3-0003 -> 3-0002 (2-0016 -> 2-0033 (1-0019 -> 1-0076 ([1276] -> [1482], 418.268398))), 3-0004 -> 3-0003 (2-0027 -> 2-0004 (1-0047 -> 1-0138 ([1760] -> [1223], 457.613075))), 3-0007 -> 3-0004 (2-0024 -> 2-0026 (1-0132 -> 1-0065 ([1086] -> [0375], 8380.073845))),

Total Graphs For Level - 4 : 2

Level 5

=======

Graph 5-0001 : 4-0001 -> 4-0002 (3-0005 -> 3-0002 (2-0006 -> 2-0034 (1-0006 -> 1-0071 ([1361] -> [1277], 436.340836)))), 4-0002 -> 4-0001 (3-0002 -> 3-0005 (2-0034 -> 2-0006 (1-0071 -> 1-0006 ([1277] -> [1361], 436.340836)))),

Total Graphs For Level - 5 : 1

Graph description of Baltimore class VI:

Level 1

=======

Graph 1-0001 : 0002 -> 1072 (165.936106), 1072 -> 0002 (165.936106), 1612 -> 0002 (472.302638),

Graph 1-0002 : 0044 -> 1004 (277.328385), 1004 -> 0044 (277.328385), 1556 -> 0044 (287.939698), 1995 -> 1004 (728.428558),

Graph 1-0003 : 0098 -> 0812 (698.238645), 0629 -> 1581 (841.530451), 0812 -> 1581 (441.142595), 1581 -> 0812 (441.142595), 2022 -> 0098 (776.051486),

Graph 1-0004 : 0105 -> 1959 (653.676330), 0110 -> 1959 (500.450070), 1095 -> 0110 (577.984182), 1959 -> 0110 (500.450070),

Graph 1-0005 : 0107 -> 0658 (664.000150), 0658 -> 2013 (524.953153), 2013 -> 2025 (360.321213), 2025 -> 2013 (360.321213),

Graph 1-0006 : 0108 -> 0589 (962.893673), 0589 -> 0878 (48.405483), 0878 -> 0589 (48.405483), 1243 -> 0589 (95.631112),

Graph 1-0007 : 0225 -> 0597 (705.525209), 0597 -> 0225 (705.525209),

Graph 1-0008 : 0229 -> 1078 (495.571136), 0598 -> 0229 (586.253848), 1039 -> 1078 (538.001031), 1078 -> 0229 (495.571136),

Graph 1-0009 : 0231 -> 0853 (345.371845), 0853 -> 0231 (345.371845),

Graph 1-0010 : 0301 -> 1994 (153.600338), 0630 -> 1245 (536.774921), 0811 -> 1994 (360.667300), 1245 -> 1994 (142.262025), 1994 -> 1245 (142.262025),

Graph 1-0011 : 0631 -> 1452 (207.047591), 1452 -> 0631 (207.047591),

Graph 1-0012 : 0653 -> 1450 (57.911478), 1071 -> 0653 (145.484869), 1450 -> 0653 (57.911478), 1457 -> 1071 (341.783681),

Graph 1-0013 : 0678 -> 1098 (192.359218), 1098 -> 0678 (192.359218), 1347 -> 0678 (386.642406), 1564 -> 1347 (574.667368),

Graph 1-0014 : 0854 -> 1567 (196.121710), 0855 -> 1567 (167.365911), 1566 -> 0854 (213.329646), 1567 -> 0855 (167.365911),

Graph 1-0015 : 1477 -> 1624 (1185.741347), 1565 -> 1568 (166.630511), 1568 -> 1565 (166.630511), 1624 -> 1565 (443.737324),

Graph 1-0016 : 1558 -> 1560 (457.817895), 1559 -> 1560 (265.105713), 1560 -> 1559 (265.105713),

Total Graphs For Level - 1 : 16

Level 2

=======

Graph 2-0001 : 1-0001 -> 1-0005 ([1072] -> [2013], 776.121246), 1-0004 -> 1-0005 ([1095] -> [0107], 705.149137), 1-0005 -> 1-0004 ([0107] -> [1095], 705.149137),

Graph 2-0002 : 1-0002 -> 1-0007 ([1995] -> [0597], 964.847291), 1-0007 -> 1-0002 ([0597] -> [1995], 964.847291),

Graph 2-0003 : 1-0003 -> 1-0016 ([0812] -> [1558], 545.082985), 1-0010 -> 1-0016 ([0811] -> [1559], 466.802229), 1-0016 -> 1-0010 ([1559] -> [0811], 466.802229),

Graph 2-0004 : 1-0006 -> 1-0013 ([0878] -> [1564], 898.429187), 1-0008 -> 1-0013 ([1039] -> [1564], 597.217925), 1-0011 -> 1-0012 ([1452] -> [0653], 227.659616), 1-0012 -> 1-0011 ([0653] -> [1452], 227.659616), 1-0013 -> 1-0012 ([0678] -> [1450], 252.217833),

Graph 2-0005 : 1-0009 -> 1-0014 ([0853] -> [1567], 432.939023), 1-0014 -> 1-0015 ([1566] -> [1568], 225.383222), 1-0015 -> 1-0014 ([1568] -> [1566], 225.383222),

Total Graphs For Level - 2 : 5

Level 3

=======

Graph 3-0001 : 2-0001 -> 2-0004 (1-0001 -> 1-0006 ([1612] -> [0108], 1246.732081)), 2-0002 -> 2-0003 (1-0007 -> 1-0003 ([0225] -> [1581], 1542.320669)), 2-0003 -> 2-0004 (1-0010 -> 1-0008 ([0811] -> [1078], 903.194690)), 2-0004 -> 2-0005 (1-0011 -> 1-0015 ([1452] -> [1624], 586.288082)), 2-0005 -> 2-0004 (1-0015 -> 1-0011 ([1624] -> [1452], 586.288082)),

Total Graphs For Level - 3 : 1

## List of virus information

| No. | Virus Name | Accession | Baltimore |
|---|---|---|---|
| 1 | Abalone shriveling syndrome associated virus uid33141 | NC 011646.1 | I |
| 2 | Abelson murine leukemia virus uid14654 | NC 001499.1 | VI |
| 3 | Acanthocystis turfacea Chlorella virus 1 uid18527 | NC 008724.1 | I |
| 4 | Acheta domestica densovirus uid15222 | NC 004290.1 | II |
| 5 | Acholeplasma phage L2 uid14066 | NC 001447.1 | I |
| 6 | Acholeplasma phage MV L1 uid14573 | NC 001341.1 | II |
| 7 | Acidianus bottle shaped virus uid19605 | NC 009452.1 | I |
| 8 | Acidianus filamentous virus 1 uid14363 | NC 005830.1 | I |
| 9 | Acidianus filamentous virus 2 uid20965 | NC 009884.1 | I |
| 10 | Acidianus filamentous virus 3 uid28073 | NC 010155.1 | I |
| 11 | Acidianus filamentous virus 6 uid28075 | NC 010152.1 | I |
| 12 | Acidianus filamentous virus 7 uid28077 | NC 010153.1 | I |
| 13 | Acidianus filamentous virus 8 uid28079 | NC 010154.1 | I |
| 14 | Acidianus filamentous virus 9 uid29195 | NC 010537.1 | I |
| 15 | Acidianus rod shaped virus 1 uid27799 | NC 009965.1 | I |
| 16 | Acidianus spindle shaped virus 1 uid42351 | NC 013585.1 | I |
| 17 | Acidianus two tailed virus uid15686 | NC 007409.1 | I |
| 18 | Acinetobacter phage AP205 uid14710 | NC 002700.2 | IV |
| 19 | Aconitum latent virus uid15382 | NC 002795.1 | IV |
| 20 | Actinomyces phage Av 1 uid20057 | NC 009643.1 | I |
| 21 | Actinoplanes phage phiAsp2 uid14378 | NC 005885.1 | I |
| 22 | Acute bee paralysis virus uid14983 | NC 002548.1 | IV |
| 23 | Acyrthosiphon pisum secondary endosymbiont phage 1 uid14047 | NC 000935.1 | I |
| 24 | Acyrthosiphon pisum virus uid40357 | NC 003780.1 | IV |
| 25 | Adeno associated virus 1 uid15323 | NC 002077.1 | II |
| 26 | Adeno associated virus 2 uid14060 | NC 001401.2 | II |
| 27 | Adeno associated virus 3 uid14319 | NC 001729.1 | II |
| 28 | Adeno associated virus 4 uid14030 | NC 001829.1 | II |
| 29 | Adeno associated virus 5 uid14426 | NC 006152.1 | II |
| 30 | Adeno associated virus 7 uid14454 | NC 006260.1 | II |
| 31 | Adeno associated virus 8 uid14455 | NC 006261.1 | II |
| 32 | Adoxophyes honmai NPV uid14408 | NC 004690.1 | I |
| 33 | Adoxophyes orana granulovirus uid14298 | NC 005038.1 | I |
| 34 | Adoxophyes orana nucleopolyhedrovirus uid32387 | NC 011423.1 | I |
| 35 | Aedes aegypti densovirus uid37821 | NC 012636.1 | II |
| 36 | Aedes albopictus densovirus uid14581 | NC 004285.1 | II |
| 37 | Aedes flavivirus uid39601 | NC 012932.1 | IV |
| 38 | Aeromonas phage 25 uid17105 | NC 008208.1 | I |
| 39 | Aeromonas phage 31 uid15416 | NC 007022.1 | I |
| 40 | Aeromonas phage 44RR2 8t uid14321 | NC 005135.1 | I |
| 41 | Aeromonas phage Aeh1 uid14312 | NC 005260.1 | I |
| 42 | Aeromonas phage phiO18P uid19769 | NC 009542.2 | I |
| 43 | African green monkey polyomavirus uid15320 | NC 004763.1 | I |
| 44 | African green monkey simian foamy virus uid30095 | NC 010820.1 | VI |
| 45 | African oil palm ringspot virus uid36557 | NC 012519.1 | IV |
| 46 | African swine fever virus uid15242 | NC 001659.1 | I |
| 47 | Ageratum enation virus uid15192 | NC 003434.1 | II |

| | | | |
|---|---|---|---|
| 48 | Ageratum leaf curl Cameroon betasatellite uid36669 | NC 012557.1 | NA |
| 49 | Ageratum leaf curl disease associated satellite DNA beta uid14439 | NC 005046.1 | NA |
| 50 | Ageratum leaf curl virus G52 uid14492 | NC 006384.1 | II |
| 51 | Ageratum yellow leaf curl beta Pakistan Lahore 2005 uid33363 | NC 011762.1 | NA |
| 52 | Ageratum yellow vein China virus associated DNA beta uid15515 | NC 007067.1 | NA |
| 53 | Ageratum yellow vein China virus uid14490 | NC 004090.1 | II |
| 54 | Ageratum yellow vein Hualian virus uid30057 | NC 010812.1 | II |
| 55 | Ageratum yellow vein Sri Lanka virus uid14120 | NC 002981.1 | II |
| 56 | Ageratum yellow vein Taiwan virus uid14249 | NC 004627.1 | II |
| 57 | Ageratum yellow vein virus satellite DNA beta uid14444 | NC 003403.1 | NA |
| 58 | Ageratum yellow vein virus uid15203 | NC 004626.1 | II |
| 59 | Agropyron mosaic virus uid15063 | NC 005903.1 | IV |
| 60 | Agrotis ipsilon multiple nucleopolyhedrovirus uid32171 | NC 011345.1 | I |
| 61 | Agrotis segetum granulovirus uid14481 | NC 005839.2 | I |
| 62 | Agrotis segetum nucleopolyhedrovirus uid16661 | NC 007921.1 | I |
| 63 | Aichi virus uid15441 | NC 001918.1 | IV |
| 64 | Alcelaphine herpesvirus 1 uid14099 | NC 002531.1 | I |
| 65 | Aleutian mink disease virus uid14077 | NC 001662.1 | II |
| 66 | Algerian watermelon mosaic virus uid29883 | NC 010736.1 | IV |
| 67 | Alkhurma hemorrhagic fever virus uid15387 | NC 004355.1 | IV |
| 68 | Allamanda leaf curl virus uid30179 | NC 010947.1 | II |
| 69 | Allium virus X uid34843 | NC 012211.2 | IV |
| 70 | Alstroemeria virus x uid15687 | NC 007408.1 | IV |
| 71 | Alternanthera mosaic virus uid16333 | NC 007731.1 | IV |
| 72 | Alternanthera yellow vein virus satellite DNA beta uid19833 | NC 009562.1 | NA |
| 73 | Alternanthera yellow vein virus uid15560 | NC 007211.1 | II |
| 74 | Ambystoma tigrinum virus uid14364 | NC 005832.1 | I |
| 75 | Amsacta moorei entomopoxvirus L uid14097 | NC 002520.1 | I |
| 76 | Anagyris vein yellowing virus uid32713 | NC 011559.1 | IV |
| 77 | Angelonia flower break virus uid16334 | NC 007733.1 | IV |
| 78 | Anguillid herpesvirus 1 uid42931 | NC 013668.1 | I |
| 79 | Anopheles gambiae densonucleosis virus uid32101 | NC 011317.1 | II |
| 80 | Antheraea pernyi nucleopolyhedrovirus uid16793 | NC 008035.3 | I |
| 81 | Anticarsia gemmatalis nucleopolyhedrovirus uid17995 | NC 008520.1 | I |
| 82 | Aphid lethal paralysis virus uid14867 | NC 004365.1 | IV |
| 83 | Apoi virus uid15369 | NC 003676.1 | IV |
| 84 | Apple chlorotic leaf spot virus uid14658 | NC 001409.1 | IV |
| 85 | Apple stem grooving virus uid15119 | NC 001749.2 | IV |
| 86 | Apple stem pitting virus uid14744 | NC 003462.1 | IV |
| 87 | Apricot pseudo chlorotic leaf spot virus uid15172 | NC 006946.1 | IV |
| 88 | Arabis mosaic virus large satellite RNA uid14752 | NC 003523.1 | NA |
| 89 | Arabis mosaic virus small satellite RNA uid14021 | NC 001546.1 | NA |
| 90 | Archaeal BJ1 virus uid18503 | NC 008695.1 | I |
| 91 | Aroa virus uid18847 | NC 009026.2 | IV |
| 92 | Artichoke mottled crinkle virus uid15517 | NC 001339.1 | IV |
| 93 | Asparagus virus 3 uid28979 | NC 010416.1 | IV |
| 94 | Astrovirus MLB1 HK05 uid50359 | NC 014320.1 | IV |
| 95 | Astrovirus MLB1 uid32327 | NC 011400.1 | IV |
| 96 | Astrovirus VA1 uid39811 | NC 013060.1 | IV |

| | | | |
|---|---|---|---|
| 97 | Ateline herpesvirus 3 uid14040 | NC 001987.1 | I |
| 98 | Atlantic salmon swim bladder sarcoma virus uid16247 | NC 007654.1 | VI |
| 99 | Augustine decline satellite virus uid14898 | NC 005099.1 | NA |
| 100 | Aura virus uid14830 | NC 003900.1 | IV |
| 101 | Australian bat lyssavirus uid14730 | NC 003243.1 | V |
| 102 | Autographa californica nucleopolyhedrovirus uid14023 | NC 001623.1 | I |
| 103 | Avian adeno associated virus ATCC VR 865 uid14456 | NC 004828.1 | II |
| 104 | Avian adeno associated virus DA 1 uid14463 | NC 006263.1 | II |
| 105 | Avian carcinoma virus uid14632 | NC 001402.1 | VI |
| 106 | Avian encephalomyelitis virus uid15360 | NC 003990.1 | IV |
| 107 | Avian endogenous retrovirus EAV HP uid15213 | NC 005947.1 | VI |
| 108 | Avian leukosis virus uid14633 | NC 001408.1 | VI |
| 109 | Avian metapneumovirus uid16240 | NC 007652.1 | V |
| 110 | Avian myelocytomatosis virus uid14909 | NC 001866.1 | VI |
| 111 | Avian paramyxovirus 6 uid14719 | NC 003043.1 | V |
| 112 | Azospirillum phage Cd uid28841 | NC 010355.1 | I |
| 113 | Bacillus phage 0305phi8 36 uid20653 | NC 009760.1 | I |
| 114 | Bacillus phage AP50 uid32599 | NC 011523.1 | I |
| 115 | Bacillus phage B103 uid14216 | NC 004165.1 | I |
| 116 | Bacillus phage Bam35c uid14311 | NC 005258.1 | I |
| 117 | Bacillus phage BCJA1c uid14548 | NC 006557.1 | I |
| 118 | Bacillus phage Cherry uid15784 | NC 007457.1 | I |
| 119 | Bacillus phage Fah uid16382 | NC 007814.1 | I |
| 120 | Bacillus phage GA 1 uid15202 | NC 002649.1 | I |
| 121 | Bacillus phage Gamma uid15783 | NC 007458.1 | I |
| 122 | Bacillus phage GIL16c uid15164 | NC 006945.1 | I |
| 123 | Bacillus phage IEBH uid31057 | NC 011167.1 | I |
| 124 | Bacillus phage phBC6A51 uid15021 | NC 004820.1 | |
| 125 | Bacillus phage phBC6A52 uid15022 | NC 004821.1 | |
| 126 | Bacillus phage phi105 uid14217 | NC 004167.1 | I |
| 127 | Bacillus phage phi29 uid30615 | NC 011048.1 | I |
| 128 | Bacillus phage SPBc2 uid14034 | NC 001884.1 | I |
| 129 | Bacillus phage SPO1 uid32379 | NC 011421.1 | I |
| 130 | Bacillus phage SPP1 uid14586 | NC 004166.2 | I |
| 131 | Bacillus phage TP21 L uid33139 | NC 011645.1 | I |
| 132 | Bacillus phage WBeta uid16329 | NC 007734.1 | I |
| 133 | Bacillus virus 1 uid20397 | NC 009737.2 | I |
| 134 | Bacteriophage APSE 2 uid32705 | NC 011551.1 | I |
| 135 | Bacteroides phage B40 8 uid31249 | NC 011222.1 | I |
| 136 | Bagaza virus uid36619 | NC 012534.1 | IV |
| 137 | Bamboo mosaic virus satellite RNA uid14748 | NC 003497.1 | NA |
| 138 | Bamboo mosaic virus uid14728 | NC 001642.1 | IV |
| 139 | Banana bract mosaic virus uid20617 | NC 009745.1 | IV |
| 140 | Banana mild mosaic virus uid14711 | NC 002729.1 | IV |
| 141 | Banana streak GF virus uid15411 | NC 007002.1 | VII |
| 142 | Banana streak Mys virus uid15234 | NC 006955.1 | VII |
| 143 | Banana streak OL virus uid15239 | NC 003381.1 | VII |
| 144 | Banana streak virus Acuminata Vietnam uid15240 | NC 007003.1 | VII |
| 145 | Banana streak virus uid16747 | NC 008018.1 | VII |
| 146 | Bandicoot papillomatosis carcinomatosis virus type 1 uid27985 | NC 010107.1 | I |

| | | | |
|---|---|---|---|
| 147 | Bandicoot papillomatosis carcinomatosis virus type 2 uid30081 | NC 010817.1 | I |
| 148 | Barley dwarf virus uid30035 | NC 010798.1 | II |
| 149 | Barley yellow dwarf virus GAV uid15035 | NC 004666.1 | IV |
| 150 | Barley yellow dwarf virus MAV uid14781 | NC 003680.1 | IV |
| 151 | Barley yellow dwarf virus PAS uid14698 | NC 002160.2 | IV |
| 152 | Barley yellow dwarf virus PAV uid15196 | NC 004750.1 | IV |
| 153 | Barmah Forest virus uid14679 | NC 001786.1 | IV |
| 154 | Basella rugose mosaic virus uid20619 | NC 009741.1 | IV |
| 155 | Bat adeno associated virus YNM uid51735 | NC 014468.1 | II |
| 156 | Bat coronavirus  BtCoV 133 2005  uid17585 | NC 008315.1 | IV |
| 157 | Bat coronavirus 1A uid29247 | NC 010437.1 | IV |
| 158 | Bat coronavirus 1B uid29249 | NC 010436.1 | IV |
| 159 | Bat coronavirus BM48 31 BGR 2008 uid51751 | NC 014470.1 | IV |
| 160 | Bat coronavirus HKU2 uid27911 | NC 009988.1 | IV |
| 161 | Bat coronavirus HKU4 uid18863 | NC 009019.1 | IV |
| 162 | Bat coronavirus HKU5 uid18865 | NC 009020.1 | IV |
| 163 | Bat coronavirus HKU8 uid29245 | NC 010438.1 | IV |
| 164 | Bat coronavirus HKU9 uid18867 | NC 009021.1 | IV |
| 165 | Bdellovibrio phage phiMH2K uid14107 | NC 002643.1 | II |
| 166 | Beak and feather disease virus uid14453 | NC 001944.1 | II |
| 167 | Bean common mosaic necrosis virus uid15333 | NC 004047.1 | IV |
| 168 | Bean common mosaic virus uid15183 | NC 003397.1 | IV |
| 169 | Bean leafroll virus uid14734 | NC 003369.1 | IV |
| 170 | Bean yellow dwarf virus uid14605 | NC 003493.2 | II |
| 171 | Bean yellow mosaic virus uid15339 | NC 003492.1 | IV |
| 172 | Beet black scorch virus satellite RNA uid14623 | NC 006460.1 | NA |
| 173 | Beet black scorch virus uid14949 | NC 004452.3 | IV |
| 174 | Beet chlorosis virus uid14712 | NC 002766.1 | IV |
| 175 | Beet curly top Iran virus uid28973 | NC 010417.1 | II |
| 176 | Beet curly top virus uid14366 | NC 001412.1 | II |
| 177 | Beet mild curly top virus uid14282 | NC 004753.1 | II |
| 178 | Beet mild yellowing virus uid15079 | NC 003491.1 | IV |
| 179 | Beet mosaic virus uid14942 | NC 005304.1 | IV |
| 180 | Beet severe curly top virus uid14367 | NC 004754.1 | II |
| 181 | Beet western yellows ST9 associated virus uid14910 | NC 004045.1 | IV |
| 182 | Beet western yellows virus uid14885 | NC 004756.1 | IV |
| 183 | Beet yellows virus uid15328 | NC 001598.1 | IV |
| 184 | Begomovirus associated DNA II uid15161 | NC 006956.1 | NA |
| 185 | Begomovirus associated DNA III uid15162 | NC 006957.1 | NA |
| 186 | Beilong virus uid16630 | NC 007803.1 | V |
| 187 | Bell pepper mottle tobamovirus uid20059 | NC 009642.1 | IV |
| 188 | Bettongia penicillata papillomavirus 1 uid48601 | NC 014143.1 | I |
| 189 | Bhendi yellow vein Bhubhaneswar virus uid33885 | NC 012041.1 | II |
| 190 | Bhendi yellow vein Delhi virus  2004 New Delhi  uid33677 | NC 011919.1 | II |
| 191 | Bhendi yellow vein mosaic virus satellite DNA beta uid14445 | NC 003405.1 | NA |
| 192 | Bhendi yellow vein mosaic virus uid14159 | NC 003418.1 | II |
| 193 | Bidens mottle virus uid50559 | NC 014325.1 | IV |
| 194 | Bitter gourd leaf curl disease associated DNA beta uid16245 | NC 007655.1 | NA |
| 195 | BK polyomavirus uid14074 | NC 001538.1 | I |
| 196 | Black queen cell virus uid14803 | NC 003784.1 | IV |

| | | | |
|---|---|---|---|
| 197 | Black raspberry virus F uid20975 | NC 009890.1 | III |
| 198 | Blackberry virus Y uid18125 | NC 008558.1 | IV |
| 199 | Blackcurrant reversion virus satellite RNA uid14821 | NC 003872.1 | NA |
| 200 | Blattella germanica densovirus uid14320 | NC 005041.2 | II |
| 201 | Blueberry red ringspot virus uid14129 | NC 003138.2 | VII |
| 202 | Blueberry scorch virus uid15329 | NC 003499.1 | IV |
| 203 | Bocavirus gorilla GBoV1 2009 uid51179 | NC 014358.1 | II |
| 204 | Bombyx mandarina nucleopolyhedrovirus uid37971 | NC 012672.1 | I |
| 205 | Bombyx mori cypovirus 1 satellite RNA uid14557 | NC 006630.1 | NA |
| 206 | Bombyx mori densovirus 5 uid15444 | NC 004287.1 | II |
| 207 | Bombyx mori NPV uid14089 | NC 001962.1 | I |
| 208 | Border disease virus uid15463 | NC 003679.1 | IV |
| 209 | Bordetella phage BIP 1 uid14359 | NC 005809.1 | I |
| 210 | Bordetella phage BMP 1 uid14358 | NC 005808.1 | I |
| 211 | Bordetella phage BPP 1 uid14353 | NC 005357.1 | I |
| 212 | Borna disease virus uid14675 | NC 001607.1 | V |
| 213 | Botryotinia fuckeliana totivirus 1 uid19133 | NC 009224.1 | III |
| 214 | Botrytis cinerea debilitation related virus uid32247 | NC 011372.1 | IV |
| 215 | Botrytis virus F uid14707 | NC 002604.1 | IV |
| 216 | Botrytis virus X uid14947 | NC 005132.1 | IV |
| 217 | Bougainvillea spectabilis chlorotic vein banding virus uid32823 | NC 011592.1 | VII |
| 218 | Bovine adeno associated virus uid14381 | NC 005889.1 | II |
| 219 | Bovine adenovirus A uid14612 | NC 006324.1 | I |
| 220 | Bovine adenovirus B uid14515 | NC 001876.1 | I |
| 221 | Bovine adenovirus D uid14486 | NC 002685.2 | I |
| 222 | Bovine coronavirus uid15385 | NC 003045.1 | IV |
| 223 | Bovine enterovirus uid15351 | NC 001859.1 | IV |
| 224 | Bovine ephemeral fever virus uid14434 | NC 002526.1 | V |
| 225 | Bovine foamy virus uid14646 | NC 001831.1 | VI |
| 226 | Bovine herpesvirus 1 uid14585 | NC 001847.1 | I |
| 227 | Bovine herpesvirus 4 uid14110 | NC 002665.1 | I |
| 228 | Bovine herpesvirus 5 uid14313 | NC 005261.2 | I |
| 229 | Bovine immunodeficiency virus uid14634 | NC 001413.1 | VI |
| 230 | Bovine kobuvirus uid14948 | NC 004421.1 | IV |
| 231 | Bovine leukemia virus uid14916 | NC 001414.1 | VI |
| 232 | Bovine papillomavirus 1 uid15513 | NC 001522.1 | I |
| 233 | Bovine papillomavirus 5 uid14220 | NC 004195.1 | I |
| 234 | Bovine papillomavirus 8 uid20637 | NC 009752.1 | I |
| 235 | Bovine papillomavirus 3 uid15452 | NC 004197.1 | I |
| 236 | Bovine papular stomatitis virus uid14469 | NC 005337.1 | I |
| 237 | Bovine parainfluenza virus 3 uid15001 | NC 002161.1 | V |
| 238 | Bovine parvovirus 2 uid14553 | NC 006259.1 | II |
| 239 | Bovine parvovirus uid14020 | NC 001540.1 | II |
| 240 | Bovine polyomavirus uid14017 | NC 001442.1 | I |
| 241 | Bovine respiratory coronavirus AH187 uid39331 | NC 012948.1 | IV |
| 242 | Bovine respiratory coronavirus bovine US OH 440 TC 1996 uid39333 | NC 012949.1 | IV |
| 243 | Bovine respiratory syncytial virus uid14697 | NC 001989.1 | V |
| 244 | Bovine rhinovirus 2 uid28835 | NC 010354.1 | IV |
| 245 | Bovine viral diarrhea virus 1 uid15305 | NC 001461.1 | IV |

| | | | |
|---|---|---|---|
| 246 | Bovine viral diarrhea virus 2 uid15089 | NC 002032.1 | IV |
| 247 | Bovine viral diarrhea virus 3 Th 04 KhonKaen uid38557 | NC 012812.1 | IV |
| 248 | Breda virus uid15881 | NC 007447.1 | IV |
| 249 | Brevicoryne brassicae picorna like virus uid19753 | NC 009530.1 | IV |
| 250 | Brome streak mosaic virus uid15336 | NC 003501.1 | IV |
| 251 | Brugmansia tobamovirus uid30157 | NC 010944.1 | IV |
| 252 | Bundibugyo ebolavirus uid51245 | NC 014373.1 | V |
| 253 | Burkholderia ambifaria phage BcepF1 uid18857 | NC 009015.1 | I |
| 254 | Burkholderia phage Bcep1 uid14409 | NC 005263.2 | I |
| 255 | Burkholderia phage Bcep176 uid16102 | NC 007497.1 | I |
| 256 | Burkholderia phage Bcep22 uid14335 | NC 005262.2 | I |
| 257 | Burkholderia phage Bcep43 uid14411 | NC 005342.2 | I |
| 258 | Burkholderia phage Bcep781 uid14405 | NC 004333.2 | I |
| 259 | Burkholderia phage BcepB1A uid14476 | NC 005886.2 | I |
| 260 | Burkholderia phage BcepC6B uid14379 | NC 005887.1 | I |
| 261 | Burkholderia phage BcepGomr uid19579 | NC 009447.1 | I |
| 262 | Burkholderia phage BcepIL02 uid38297 | NC 012743.2 | I |
| 263 | Burkholderia phage BcepMu uid14376 | NC 005882.1 | I |
| 264 | Burkholderia phage BcepNazgul uid14305 | NC 005091.2 | I |
| 265 | Burkholderia phage BcepNY3 uid19963 | NC 009604.1 | I |
| 266 | Burkholderia phage KS10 uid31221 | NC 011216.1 | I |
| 267 | Burkholderia phage KS9 uid39771 | NC 013055.1 | I |
| 268 | Burkholderia phage phi1026b uid14410 | NC 005284.1 | I |
| 269 | Burkholderia phage phi52237 uid15422 | NC 007145.2 | I |
| 270 | Burkholderia phage phi644 2 uid62941 | NC 009235.2 | I |
| 271 | Burkholderia phage phiE12 2 uid19161 | NC 009236.1 | I |
| 272 | Burkholderia phage phiE125 uid14330 | NC 003309.1 | I |
| 273 | Burkholderia phage phiE202 uid19163 | NC 009234.1 | I |
| 274 | Burkholderia phage phiE255 uid19165 | NC 009237.1 | I |
| 275 | Butterbur mosaic virus uid42145 | NC 013527.1 | IV |
| 276 | Cacao swollen shoot virus uid14534 | NC 001574.1 | VII |
| 277 | Cactus mild mottle virus uid33485 | NC 011803.1 | IV |
| 278 | Cactus virus X uid14996 | NC 002815.2 | IV |
| 279 | Calicivirus isolate TCG uid15123 | NC 006875.1 | IV |
| 280 | Calicivirus NB uid14845 | NC 004064.1 | IV |
| 281 | Calicivirus pig AB90 CAN uid38093 | NC 012699.1 | IV |
| 282 | California sea lion anellovirus uid34735 | NC 012126.1 | II |
| 283 | California sea lion polyomavirus 1 uid45909 | NC 013796.1 | I |
| 284 | Callitrichine herpesvirus 3 uid14324 | NC 004367.1 | I |
| 285 | Camelpox virus uid14156 | NC 003391.1 | I |
| 286 | Canary circovirus uid14513 | NC 003410.1 | II |
| 287 | Canarypox virus uid14340 | NC 005309.1 | I |
| 288 | Canine adenovirus uid14516 | NC 001734.1 | I |
| 289 | Canine calicivirus uid14875 | NC 004542.1 | IV |
| 290 | Canine distemper virus uid15002 | NC 001921.1 | V |
| 291 | Canine minute virus uid15465 | NC 004442.1 | II |
| 292 | Canine oral papillomavirus uid14326 | NC 001619.1 | I |
| 293 | Canine papillomavirus 4 uid28243 | NC 010226.1 | I |
| 294 | Canine papillomavirus 2 uid14551 | NC 006564.1 | I |
| 295 | Canine papillomavirus 3 uid17543 | NC 008297.1 | I |

| | | | |
|---|---|---|---|
| 296 | Canine papillomavirus 5 uid40367 | NC 013236.1 | I |
| 297 | Canine papillomavirus 6 uid40369 | NC 013237.1 | I |
| 298 | Canine parvovirus uid14614 | NC 001539.1 | II |
| 299 | Canna Yellow Streak Virus uid40629 | NC 013261.1 | IV |
| 300 | Capra hircus papillomavirus type 1 uid16815 | NC 008032.1 | I |
| 301 | Caprine arthritis encephalitis virus uid15243 | NC 001463.1 | VI |
| 302 | Cardamine chlorotic fleck virus uid14674 | NC 001600.1 | IV |
| 303 | Cardiospermum yellow leaf curl virus satellite DNA beta uid28647 | NC 010297.1 | NA |
| 304 | Caretta papillomavirus 1 uid32633 | NC 011530.1 | I |
| 305 | Carnation etched ring virus uid14494 | NC 003498.1 | VII |
| 306 | Carnation Italian ringspot virus uid15077 | NC 003500.2 | IV |
| 307 | Carnation mottle virus uid14993 | NC 001265.1 | IV |
| 308 | Carrot mottle mimic virus uid15085 | NC 001726.1 | IV |
| 309 | Carrot red leaf luteovirus associated RNA uid14820 | NC 003871.1 | IV |
| 310 | Carrot red leaf virus uid15057 | NC 006265.1 | IV |
| 311 | Carrot yellow leaf virus uid39585 | NC 013007.1 | IV |
| 312 | Casphalia extranea densovirus uid14222 | NC 004288.1 | II |
| 313 | Cassava brown streak virus uid38085 | NC 012698.1 | IV |
| 314 | Cassava common mosaic virus uid14705 | NC 001658.1 | IV |
| 315 | Cassava vein mosaic virus uid14056 | NC 001648.1 | VII |
| 316 | Cauliflower mosaic virus uid14574 | NC 001497.1 | VII |
| 317 | Caviid herpesvirus 2 uid32779 | NC 011587.1 | I |
| 318 | Cell fusing agent virus uid15326 | NC 001564.1 | IV |
| 319 | Cercopithecine herpesvirus 2 uid14558 | NC 006560.1 | I |
| 320 | Cercopithecine herpesvirus 5 uid38429 | NC 012783.2 | I |
| 321 | Cercopithecine herpesvirus 9 uid14596 | NC 002686.2 | I |
| 322 | Cereal yellow dwarf virus RPS uid14691 | NC 002198.2 | IV |
| 323 | Cereal yellow dwarf virus RPV uid14883 | NC 004751.1 | IV |
| 324 | Cereal yellow dwarf virus RPV satellite RNA uid14169 | NC 003533.1 | NA |
| 325 | Cestrum yellow leaf curling virus uid14470 | NC 004324.3 | VII |
| 326 | Chaetoceros salsugineum DNA virus uid15497 | NC 007193.2 | |
| 327 | Chaetoceros socialis f radians RNA virus uid34845 | NC 012212.1 | IV |
| 328 | Chalara elegans RNA Virus 1 uid15126 | NC 005883.1 | III |
| 329 | Chayote mosaic virus uid15420 | NC 002588.1 | IV |
| 330 | Chayote yellow mosaic virus uid15193 | NC 004618.1 | II |
| 331 | Chelonia mydas papillomavirus 1 uid32635 | NC 011531.1 | I |
| 332 | Chenopodium mosaic virus X uid17349 | NC 008251.1 | IV |
| 333 | Cherry green ring mottle virus uid14650 | NC 001946.1 | IV |
| 334 | Cherry mottle leaf virus uid14695 | NC 002500.1 | IV |
| 335 | Cherry necrotic rusty mottle virus uid14729 | NC 002468.1 | IV |
| 336 | Cherry virus A uid15080 | NC 003689.1 | IV |
| 337 | Chicken anemia virus uid15484 | NC 001427.1 | II |
| 338 | Chicken astrovirus uid14804 | NC 003790.1 | IV |
| 339 | Chickpea chlorotic dwarf Sudan virus uid28581 | NC 010289.1 | II |
| 340 | Chickpea chlorotic dwarf virus uid30715 | NC 011058.1 | II |
| 341 | Chickpea chlorotic stunt virus uid17363 | NC 008249.1 | IV |
| 342 | Chicory yellow mottle virus large satellite RNA uid14798 | NC 003778.1 | NA |
| 343 | Chicory yellow mottle virus satellite RNA L1 uid15019 | NC 006452.1 | NA |
| 344 | Chicory yellow mottle virus satellite RNA uid14988 | NC 003971.1 | NA |
| 345 | Chikungunya virus uid14998 | NC 004162.2 | IV |

| | | | |
|---|---|---|---|
| 346 | Chilli leaf curl Multan alphasatellite uid39933 | NC 013103.1 | NA |
| 347 | Chilli leaf curl virus satellite DNA beta uid14441 | NC 005048.1 | NA |
| 348 | Chilli leaf curl virus uid14250 | NC 004628.1 | II |
| 349 | Chilli veinal mottle virus uid15225 | NC 005778.1 | IV |
| 350 | Chiltepin yellow mosaic virus uid48419 | NC 014127.1 | IV |
| 351 | Chlamydia phage 3 uid14471 | NC 008355.1 | II |
| 352 | Chlamydia phage 4 uid15781 | NC 007461.1 | II |
| 353 | Chlamydia phage Chp1 uid14064 | NC 001741.1 | II |
| 354 | Chlamydia phage Chp2 uid14593 | NC 002194.1 | II |
| 355 | Chlamydia phage phiCPG1 uid14012 | NC 001998.1 | II |
| 356 | Chloris striate mosaic virus uid14068 | NC 001466.1 | II |
| 357 | Choristoneura fumiferana DEF MNPV uid15137 | NC 005137.2 | I |
| 358 | Choristoneura fumiferana MNPV uid15133 | NC 004778.3 | I |
| 359 | Choristoneura occidentalis granulovirus uid17097 | NC 008168.1 | I |
| 360 | Chrysanthemum virus B uid18985 | NC 009087.2 | IV |
| 361 | Chrysodeixis chalcites nucleopolyhedrovirus uid15469 | NC 007151.1 | I |
| 362 | Circovirus like genome BBC A uid39611 | NC 013020.1 | II |
| 363 | Circovirus like genome CB A uid39627 | NC 013028.1 | II |
| 364 | Circovirus like genome CB B uid39629 | NC 013029.1 | II |
| 365 | Circovirus like genome RW A uid39617 | NC 013023.1 | II |
| 366 | Circovirus like genome RW B uid39619 | NC 013024.1 | II |
| 367 | Circovirus like genome RW C uid39621 | NC 013025.1 | II |
| 368 | Circovirus like genome RW D uid39623 | NC 013026.1 | II |
| 369 | Circovirus like genome RW E uid39625 | NC 013027.1 | II |
| 370 | Circovirus like genome SAR A uid39631 | NC 013030.2 | II |
| 371 | Circovirus like genome SAR B uid39607 | NC 013018.1 | II |
| 372 | Circulifer tenellus virus 1 uid51183 | NC 014360.1 | III |
| 373 | Citrus leaf blotch virus uid14825 | NC 003877.1 | IV |
| 374 | Citrus sudden death associated virus uid15170 | NC 006950.1 | IV |
| 375 | Citrus tristeza virus uid15334 | NC 001661.1 | IV |
| 376 | Citrus yellow mosaic virus uid14153 | NC 003382.1 | VII |
| 377 | Clanis bilineata nucleopolyhedrosis virus uid17485 | NC 008293.1 | I |
| 378 | Classical swine fever virus uid15457 | NC 002657.1 | IV |
| 379 | Clavibacter phage CMP1 uid42947 | NC 013698.1 | I |
| 380 | Clerodendron yellow mosaic virus uid19599 | NC 009451.1 | II |
| 381 | Clostridium phage 39 O uid32103 | NC 011318.1 | I |
| 382 | Clostridium phage c st uid16151 | NC 007581.1 | I |
| 383 | Clostridium phage phi CD119 uid16662 | NC 007917.1 | I |
| 384 | Clostridium phage phi3626 uid14166 | NC 003524.1 | I |
| 385 | Clostridium phage phiC2 uid19153 | NC 009231.1 | I |
| 386 | Clostridium phage phiCD27 uid32323 | NC 011398.1 | I |
| 387 | Clostridium phage phiCTP1 uid51665 | NC 014457.1 | I |
| 388 | Clover yellow mosaic virus uid14645 | NC 001753.1 | IV |
| 389 | Clover yellow vein virus uid15353 | NC 003536.1 | IV |
| 390 | Cocksfoot mild mosaic virus uid30849 | NC 011108.1 | IV |
| 391 | Cocksfoot mottle virus uid15078 | NC 002618.2 | IV |
| 392 | Cocksfoot streak virus uid15399 | NC 003742.1 | IV |
| 393 | Coconut foliar decay virus uid14067 | NC 001465.1 | II |
| 394 | Coleus vein necrosis virus uid20665 | NC 009764.1 | IV |
| 395 | Columbid circovirus uid14437 | NC 002361.1 | II |

| | | | |
|---|---|---|---|
| 396 | Commelina yellow mottle virus uid14575 | NC 001343.1 | VII |
| 397 | Common chimpanzee papillomavirus 1 uid14389 | NC 001838.1 | I |
| 398 | Coniothyrium minitans mycovirus uid16142 | NC 007523.1 | III |
| 399 | Coronavirus SW1 uid29509 | NC 010646.1 | IV |
| 400 | Corynebacterium phage BFK20 uid20757 | NC 009799.2 | I |
| 401 | Corynebacterium phage P1201 uid20781 | NC 009816.1 | I |
| 402 | Cote d Ivoire ebolavirus uid51257 | NC 014372.1 | V |
| 403 | Cotton leaf curl Alabad virus uid14240 | NC 004582.1 | II |
| 404 | Cotton leaf curl Bangalore virus associated DNA beta uid15557 | NC 007219.1 | NA |
| 405 | Cotton leaf curl Bangalore virus uid15575 | NC 007290.1 | II |
| 406 | Cotton leaf curl Burewala alphasatellite uid45935 | NC 013803.1 | NA |
| 407 | Cotton leaf curl Burewala betasatellite uid45933 | NC 013802.1 | NA |
| 408 | Cotton leaf curl Burewala virus uid34757 | NC 012137.1 | II |
| 409 | Cotton leaf curl Gezira alphasatellite uid42507 | NC 013593.1 | NA |
| 410 | Cotton leaf curl Gezira beta uid20565 | NC 009740.1 | NA |
| 411 | Cotton leaf curl Gezira betasatellite uid42713 | NC 013637.1 | NA |
| 412 | Cotton leaf curl Gezira virus satellite DNA beta uid15166 | NC 006935.1 | NA |
| 413 | Cotton leaf curl Gezira virus uid14095 | NC 002510.1 | II |
| 414 | Cotton leaf curl Kokhran virus uid14241 | NC 004583.1 | II |
| 415 | Cotton leaf curl Multan betasatellite uid15780 | NC 009535.1 | NA |
| 416 | Cotton leaf curl Multan virus satellite U36 1 uid16312 | NC 007721.1 | NA |
| 417 | Cotton leaf curl Multan virus uid14242 | NC 004607.1 | II |
| 418 | Cotton leaf curl Rajasthan virus uid14130 | NC 003199.1 | II |
| 419 | Cotton leaf curl virus associated DNA beta uid14438 | NC 003200.1 | NA |
| 420 | Cottontail rabbit papillomavirus uid14075 | NC 001541.1 | I |
| 421 | Cowpea aphid borne mosaic virus uid15394 | NC 004013.1 | IV |
| 422 | Cowpea mottle virus uid14755 | NC 003535.1 | IV |
| 423 | Cowpea severe leaf curl associated DNA beta uid15157 | NC 006952.1 | NA |
| 424 | Cowpox virus uid14174 | NC 003663.2 | I |
| 425 | Crassocephalum yellow vein virus Jinghong uid18659 | NC 008794.1 | II |
| 426 | Cricket paralysis virus uid14832 | NC 003924.1 | IV |
| 427 | Crocodilepox virus uid16798 | NC 008030.1 | I |
| 428 | Croton yellow vein mosaic alphasatellite uid45931 | NC 013801.1 | NA |
| 429 | Croton yellow vein mosaic betasatellite uid18249 | NC 008579.1 | NA |
| 430 | Croton yellow vein mosaic virus uid15195 | NC 004300.1 | II |
| 431 | Croton yellow vein virus uid51789 | NC 014473.1 | |
| 432 | Crow polyomavirus uid16654 | NC 007922.1 | I |
| 433 | Crucifer tobamovirus uid14733 | NC 003355.1 | IV |
| 434 | Cryphonectria hypovirus 1 uid14664 | NC 001492.1 | III |
| 435 | Cryphonectria hypovirus 2 uid14754 | NC 003534.1 | III |
| 436 | Cryphonectria hypovirus 3 uid14690 | NC 000960.1 | III |
| 437 | Cryphonectria hypovirus 4 uid15007 | NC 006431.1 | III |
| 438 | Cryphonectria parasitica mitovirus 1 NB631 uid14838 | NC 004046.1 | IV |
| 439 | Cryptophlebia leucotreta granulovirus uid14302 | NC 005068.1 | I |
| 440 | Cucumber Bulgarian latent virus uid14881 | NC 004725.1 | IV |
| 441 | Cucumber fruit mottle mosaic virus uid14709 | NC 002633.1 | IV |
| 442 | Cucumber green mottle mosaic virus uid14681 | NC 001801.1 | IV |
| 443 | Cucumber leaf spot virus uid16590 | NC 007816.1 | IV |
| 444 | Cucumber mosaic virus satellite RNA uid14568 | NC 002602.2 | NA |
| 445 | Cucumber mottle virus uid18331 | NC 008614.1 | IV |

| | | | |
|---|---|---|---|
| 446 | Cucumber necrosis virus uid14638 | NC 001469.1 | IV |
| 447 | Cucumber vein yellowing virus uid15153 | NC 006941.1 | IV |
| 448 | Cucurbit aphid borne yellows virus uid15074 | NC 003688.1 | IV |
| 449 | Cucurbita yellow vein virus associated DNA beta uid14525 | NC 005875.1 | NA |
| 450 | Culex flavivirus uid18303 | NC 008604.2 | IV |
| 451 | Culex nigripalpus NPV uid14128 | NC 003084.1 | I |
| 452 | Culex pipiens densovirus uid37995 | NC 012685.1 | II |
| 453 | Cyanophage PSS2 uid39613 | NC 013021.1 | I |
| 454 | Cycad leaf necrosis virus uid30835 | NC 011097.1 | VII |
| 455 | Cydia pomonella granulovirus uid14118 | NC 002816.1 | I |
| 456 | Cymbidium mosaic virus uid15490 | NC 001812.1 | IV |
| 457 | Cymbidium ringspot virus satellite RNA uid14989 | NC 004009.2 | NA |
| 458 | Cymbidium ringspot virus uid15066 | NC 003532.1 | IV |
| 459 | Cyprinid herpesvirus 3 uid19059 | NC 009127.1 | I |
| 460 | Daphne mosaic virus uid16794 | NC 008028.1 | IV |
| 461 | Daphne virus S uid16749 | NC 008020.1 | IV |
| 462 | Dasheen mosaic virus uid15388 | NC 003537.1 | IV |
| 463 | Deer papillomavirus uid14073 | NC 001523.1 | I |
| 464 | Deerpox virus W 1170 84 uid32597 | NC 006967.1 | I |
| 465 | Deerpox virus W 848 83 uid15462 | NC 006966.1 | I |
| 466 | Deformed wing virus uid14891 | NC 004830.2 | IV |
| 467 | Deftia phage phiW 14 uid42945 | NC 013697.1 | I |
| 468 | Dendrolimus punctatus densovirus uid14546 | NC 006555.1 | II |
| 469 | Dengue virus 1 uid15306 | NC 001477.1 | IV |
| 470 | Dengue virus 2 uid20183 | NC 001474.2 | IV |
| 471 | Dengue virus 3 uid15598 | NC 001475.2 | IV |
| 472 | Dengue virus 4 uid15599 | NC 002640.1 | IV |
| 473 | Diadromus pulchellus ascovirus 4a uid32133 | NC 011335.1 | I |
| 474 | Diaporthe ambigua RNA virus 1 uid14962 | NC 001278.1 | IV |
| 475 | Diascia yellow mottle virus uid30795 | NC 011086.1 | IV |
| 476 | Diatraea saccharalis densovirus uid14036 | NC 001899.1 | II |
| 477 | Digitaria streak virus uid14069 | NC 001478.1 | II |
| 478 | Dioscorea bacilliform virus uid18829 | NC 009010.1 | VII |
| 479 | Diplodia scrobiculata RNA virus 1 uid43007 | NC 013699.1 | III |
| 480 | Dolichos yellow mosaic virus uid14344 | NC 005338.1 | II |
| 481 | Dolphin morbillivirus uid15215 | NC 005283.1 | V |
| 482 | Dracaena mottle virus uid16799 | NC 008034.1 | VII |
| 483 | Drosophila A virus uid39351 | NC 012958.1 | III |
| 484 | Drosophila C virus uid14682 | NC 001834.1 | IV |
| 485 | Drosophila melanogaster sigma virus AP30 uid40127 | NC 013135.1 | V |
| 486 | Drosophila melanogaster totivirus SW 2009a uid41725 | NC 013499.1 | III |
| 487 | Duck adenovirus A uid14520 | NC 001813.1 | I |
| 488 | Duck astrovirus C NGB uid36399 | NC 012437.1 | IV |
| 489 | Duck circovirus uid15558 | NC 007220.1 | II |
| 490 | Duck hepatitis B virus uid14576 | NC 001344.1 | VII |
| 491 | Duck picornavirus TW90A uid15039 | NC 006553.1 | IV |
| 492 | Dulcamara mottle virus uid16188 | NC 007609.1 | IV |
| 493 | East Asian Passiflora virus uid16326 | NC 007728.1 | IV |
| 494 | Eastern equine encephalitis virus uid15429 | NC 003899.1 | IV |
| 495 | Ecotropis obliqua NPV uid18273 | NC 008586.1 | I |

| | | | |
|---|---|---|---|
| 496 | Ectocarpus siliculosus virus 1 uid14114 | NC 002687.1 | I |
| 497 | Ectromelia virus uid14211 | NC 004105.1 | I |
| 498 | Ectropis obliqua picorna like virus uid14953 | NC 005092.1 | IV |
| 499 | Eggplant mosaic virus uid14639 | NC 001480.1 | IV |
| 500 | Eimeria brunetti RNA virus 1 uid14725 | NC 002701.1 | III |
| 501 | Emilia yellow vein virus associated DNA beta uid37893 | NC 012666.1 | NA |
| 502 | Emilia yellow vein virus Fz1 uid28689 | NC 010307.1 | II |
| 503 | Emiliania huxleyi virus 86 uid15618 | NC 007346.1 | I |
| 504 | Encephalomyocarditis virus uid15307 | NC 001479.1 | IV |
| 505 | Entebbe bat virus uid18515 | NC 008718.1 | IV |
| 506 | Enterobacteria phage 13a uid30603 | NC 011045.1 | I |
| 507 | Enterobacteria phage 933W uid14043 | NC 000924.1 | I |
| 508 | Enterobacteria phage alpha3 uid14570 | NC 001330.1 | II |
| 509 | Enterobacteria phage BA14 uid30599 | NC 011040.1 | I |
| 510 | Enterobacteria phage BP 4795 uid14287 | NC 004813.1 | I |
| 511 | Enterobacteria phage BZ13 uid14635 | NC 001426.1 | IV |
| 512 | Enterobacteria phage cdtI uid19737 | NC 009514.1 | I |
| 513 | Enterobacteria phage EcoDS1 uid30601 | NC 011042.1 | I |
| 514 | Enterobacteria phage EPS7 uid29287 | NC 010583.1 | I |
| 515 | Enterobacteria phage epsilon15 uid14285 | NC 004775.1 | I |
| 516 | Enterobacteria phage ES18 uid15174 | NC 006949.1 | I |
| 517 | Enterobacteria phage Felix 01 uid14323 | NC 005282.1 | I |
| 518 | Enterobacteria phage Fels 2 uid32273 | NC 010463.1 | I |
| 519 | Enterobacteria phage FI sensu lato uid15459 | NC 004301.1 | IV |
| 520 | Enterobacteria phage G4 sensu lato uid14318 | NC 001420.2 | II |
| 521 | Enterobacteria phage HK022 uid14048 | NC 002166.1 | I |
| 522 | Enterobacteria phage HK620 uid14115 | NC 002730.1 | I |
| 523 | Enterobacteria phage HK97 uid14592 | NC 002167.1 | I |
| 524 | Enterobacteria phage I2 2 uid14572 | NC 001332.1 | II |
| 525 | Enterobacteria phage ID18 sensu lato uid16628 | NC 007856.1 | II |
| 526 | Enterobacteria phage ID2 Moscow ID 2001 uid16591 | NC 007817.1 | II |
| 527 | Enterobacteria phage If1 uid14039 | NC 001954.1 | II |
| 528 | Enterobacteria phage Ike uid14627 | NC 002014.1 | II |
| 529 | Enterobacteria phage IME08 uid50177 | NC 014260.1 | I |
| 530 | Enterobacteria phage JK06 uid15569 | NC 007291.1 | I |
| 531 | Enterobacteria phage JS10 uid38265 | NC 012741.1 | I |
| 532 | Enterobacteria phage JS98 uid27983 | NC 010105.1 | I |
| 533 | Enterobacteria phage JSE uid38263 | NC 012740.1 | I |
| 534 | Enterobacteria phage K1 5 uid17059 | NC 008152.1 | I |
| 535 | Enterobacteria phage K1E uid16228 | NC 007637.1 | I |
| 536 | Enterobacteria phage K1F uid15880 | NC 007456.1 | I |
| 537 | Enterobacteria phage lambda uid14204 | NC 001416.1 | I |
| 538 | Enterobacteria phage M13 uid14549 | NC 003287.2 | II |
| 539 | Enterobacteria phage Min27 uid29143 | NC 010237.1 | I |
| 540 | Enterobacteria phage MS2 uid14659 | NC 001417.2 | IV |
| 541 | Enterobacteria phage Mu uid14105 | NC 000929.1 | I |
| 542 | Enterobacteria phage N15 uid14086 | NC 001901.1 | I |
| 543 | Enterobacteria phage N4 uid18511 | NC 008720.1 | I |
| 544 | Enterobacteria phage P1 uid14493 | NC 005856.1 | I |
| 545 | Enterobacteria phage P2 uid14035 | NC 001895.1 | I |

| | | | |
|---|---|---|---|
| 546 | Enterobacteria phage P22 uid14478 | NC 002371.2 | I |
| 547 | Enterobacteria phage P4 uid14414 | NC 001609.1 | I |
| 548 | Enterobacteria phage Phi1 uid20789 | NC 009821.1 | I |
| 549 | Enterobacteria phage phiEco32 uid28729 | NC 010324.1 | I |
| 550 | Enterobacteria phage phiEcoM GJ1 uid27979 | NC 010106.1 | I |
| 551 | Enterobacteria phage phiP27 uid14599 | NC 003356.1 | I |
| 552 | Enterobacteria phage phiV10 uid16381 | NC 007804.2 | I |
| 553 | Enterobacteria phage phiX174 sensu lato uid14015 | NC 001422.1 | II |
| 554 | Enterobacteria phage PRD1 uid14062 | NC 001421.2 | I |
| 555 | Enterobacteria phage PsP3 uid14345 | NC 005340.1 | I |
| 556 | Enterobacteria phage RB14 uid37825 | NC 012638.1 | I |
| 557 | Enterobacteria phage RB16 uid51699 | NC 014467.1 | I |
| 558 | Enterobacteria phage RB32 uid17997 | NC 008515.1 | I |
| 559 | Enterobacteria phage RB43 uid15417 | NC 007023.1 | I |
| 560 | Enterobacteria phage RB49 uid14301 | NC 005066.1 | I |
| 561 | Enterobacteria phage RB51 uid37819 | NC 012635.1 | I |
| 562 | Enterobacteria phage RB69 uid15141 | NC 004928.1 | I |
| 563 | Enterobacteria phage RTP uid16178 | NC 007603.1 | I |
| 564 | Enterobacteria phage Sf6 uid14498 | NC 005344.1 | I |
| 565 | Enterobacteria phage SfV uid14162 | NC 003444.1 | I |
| 566 | Enterobacteria phage SP6 uid14291 | NC 004831.2 | I |
| 567 | Enterobacteria phage SSL 2009a uid34919 | NC 012223.1 | I |
| 568 | Enterobacteria phage St 1 uid38669 | NC 012868.1 | II |
| 569 | Enterobacteria phage ST104 uid14499 | NC 005841.1 | I |
| 570 | Enterobacteria phage ST64T uid14230 | NC 004348.1 | I |
| 571 | Enterobacteria phage T1 uid14496 | NC 005833.1 | I |
| 572 | Enterobacteria phage T3 uid14336 | NC 003298.1 | I |
| 573 | Enterobacteria phage T4 uid14044 | NC 000866.4 | I |
| 574 | Enterobacteria phage T5 uid15143 | NC 005859.1 | I |
| 575 | Enterobacteria phage T7 uid14460 | NC 001604.1 | I |
| 576 | Enterobacteria phage TLS uid19775 | NC 009540.1 | I |
| 577 | Enterobacteria phage VT2 Sakai uid14480 | NC 000902.1 | I |
| 578 | Enterobacteria phage WA13 sensu lato uid16595 | NC 007821.1 | II |
| 579 | Enterobacteria phage WV8 uid38281 | NC 012749.1 | I |
| 580 | Enterobacteria phage YYZ 2008 uid32231 | NC 011356.1 | |
| 581 | Enterobacteriophage Qbeta uid15479 | NC 001890.1 | IV |
| 582 | Enterococcus phage EFAP 1 uid36375 | NC 012419.1 | I |
| 583 | Enterococcus phage phiEf11 uid42943 | NC 013696.1 | I |
| 584 | Enterococcus phage phiEF24C uid21009 | NC 009904.1 | I |
| 585 | Enterococcus phage phiFL1A uid42789 | NC 013646.1 | I |
| 586 | Enterococcus phage phiFL2A uid42795 | NC 013643.1 | I |
| 587 | Enterococcus phage phiFL3A uid42787 | NC 013648.1 | I |
| 588 | Enterococcus phage phiFL4A uid42793 | NC 013644.1 | I |
| 589 | Enzootic nasal tumour virus of goats uid14893 | NC 004994.2 | VI |
| 590 | Epiphyas postvittana NPV uid14127 | NC 003083.1 | I |
| 591 | Equid herpesvirus 1 uid14465 | NC 001491.2 | I |
| 592 | Equid herpesvirus 2 uid14457 | NC 001650.1 | I |
| 593 | Equid herpesvirus 4 uid14418 | NC 001844.1 | I |
| 594 | Equid herpesvirus 9 uid33137 | NC 011644.1 | I |
| 595 | Equine arteritis virus uid15383 | NC 002532.2 | IV |

| | | | |
|---|---|---|---|
| 596 | Equine coronavirus uid28735 | NC 010327.1 | IV |
| 597 | Equine foamy virus uid14738 | NC 002201.1 | VI |
| 598 | Equine infectious anemia virus uid14684 | NC 001450.1 | VI |
| 599 | Equine papillomavirus 1 uid14219 | NC 004194.1 | I |
| 600 | Equine papillomavirus 2 uid34709 | NC 012123.1 | I |
| 601 | Equine rhinitis A virus uid15205 | NC 003982.1 | IV |
| 602 | Equine rhinitis B virus 1 uid15206 | NC 003983.1 | IV |
| 603 | Equine rhinitis B virus 2 uid15386 | NC 003077.1 | IV |
| 604 | Equus caballus papillomavirus 1 uid15487 | NC 003748.1 | I |
| 605 | Eragrostis curvula streak virus uid37889 | NC 012664.1 | II |
| 606 | Eragrostis streak virus uid28825 | NC 010352.1 | II |
| 607 | Erectites yellow mosaic virus satellite DNA beta uid19827 | NC 009559.1 | NA |
| 608 | Erectites yellow mosaic virus uid19787 | NC 009549.1 | II |
| 609 | Erethizon dorsatum papillomavirus type 1 uid15171 | NC 006951.1 | I |
| 610 | Erinaceus europaeus papillomavirus uid33407 | NC 011765.1 | I |
| 611 | Erwinia phage Era103 uid18839 | NC 009014.1 | I |
| 612 | Erwinia phage phiEa21 4 uid33537 | NC 011811.1 | I |
| 613 | Erysimum latent virus uid14651 | NC 001977.1 | IV |
| 614 | Escherichia phage D108 uid42515 | NC 013594.1 | I |
| 615 | Escherichia phage rv5 uid30613 | NC 011041.1 | I |
| 616 | Eupatorium vein clearing virus uid29879 | NC 010738.1 | VII |
| 617 | Eupatorium yellow vein virus satellite DNA beta uid14447 | NC 004515.1 | NA |
| 618 | Eupatorium yellow vein virus uid14171 | NC 003556.1 | II |
| 619 | Euphorbia leaf curl virus uid14341 | NC 005319.1 | II |
| 620 | Euproctis pseudoconspersa nucleopolyhedrovirus uid37827 | NC 012639.1 | I |
| 621 | Euprosterna elaeasa virus uid14737 | NC 003412.1 | IV |
| 622 | European bat lyssavirus 1 uid19757 | NC 009527.1 | V |
| 623 | European bat lyssavirus 2 uid19759 | NC 009528.1 | V |
| 624 | European brown hare syndrome virus uid15087 | NC 002615.1 | IV |
| 625 | European elk papillomavirus uid15453 | NC 001524.1 | I |
| 626 | Feldmannia species virus uid31093 | NC 011183.1 | I |
| 627 | Felid herpesvirus 1 uid42429 | NC 013590.2 | I |
| 628 | Feline calicivirus uid14877 | NC 001481.2 | IV |
| 629 | Feline foamy virus uid15219 | NC 001871.1 | VI |
| 630 | Feline immunodeficiency virus uid15029 | NC 001482.1 | VI |
| 631 | Feline leukemia virus uid14686 | NC 001940.1 | VI |
| 632 | Felis domesticus papillomavirus type 1 uid14421 | NC 004765.1 | I |
| 633 | Fenneropenaeus chinensis hepatopancreatic densovirus uid51177 | NC 014357.1 | II |
| 634 | Fer de lance virus uid14985 | NC 005084.2 | V |
| 635 | Figwort mosaic virus uid14512 | NC 003554.1 | VII |
| 636 | Finch circovirus uid18021 | NC 008522.1 | II |
| 637 | Finch polyomavirus uid16655 | NC 007923.1 | I |
| 638 | Flavobacterium phage 11b uid14565 | NC 006356.2 | I |
| 639 | Foot and mouth disease virus type A uid15235 | NC 011450.1 | IV |
| 640 | Foot and mouth disease virus type Asia 1 uid14992 | NC 004915.1 | IV |
| 641 | Foot and mouth disease virus type C uid15374 | NC 002554.1 | IV |
| 642 | Foot and mouth disease virus type O uid15378 | NC 004004.1 | IV |
| 643 | Foot and mouth disease virus type SAT 1 uid15236 | NC 011451.1 | IV |
| 644 | Foot and mouth disease virus type SAT 2 uid15238 | NC 003992.2 | IV |

| | | | |
|---|---|---|---|
| 645 | Foot and mouth disease virus type SAT 3 uid15237 | NC 011452.1 | IV |
| 646 | Fort Morgan virus uid42147 | NC 013528.1 | IV |
| 647 | Fowl adenovirus A uid14522 | NC 001720.1 | I |
| 648 | Fowl adenovirus D uid14523 | NC 000899.1 | I |
| 649 | Fowlpox virus uid14052 | NC 002188.1 | I |
| 650 | Foxtail mosaic virus uid14640 | NC 001483.1 | IV |
| 651 | Francolinus leucoscepus papillomavirus 1 uid39987 | NC 013117.1 | I |
| 652 | Freesia mosaic virus uid48387 | NC 014064.1 | IV |
| 653 | Friend murine leukemia virus uid15200 | NC 001362.1 | VI |
| 654 | Fringilla coelebs papillomavirus uid14205 | NC 004068.1 | I |
| 655 | Fritillary virus Y uid30175 | NC 010954.1 | IV |
| 656 | Frog adenovirus 1 uid14488 | NC 002501.1 | I |
| 657 | Frog virus 3 uid14560 | NC 005946.1 | I |
| 658 | Fujinami sarcoma virus uid14708 | NC 001403.1 | VI |
| 659 | Fusarium graminearum dsRNA mycovirus 1 uid15154 | NC 006937.2 | III |
| 660 | Fusarium graminearum dsRNA mycovirus 3 uid41629 | NC 013469.1 | III |
| 661 | Galinsoga mosaic virus uid15209 | NC 001818.1 | IV |
| 662 | Galleria mellonella densovirus uid14221 | NC 004286.1 | II |
| 663 | Gallid herpesvirus 1 uid14566 | NC 006623.1 | I |
| 664 | Gallid herpesvirus 2 uid14402 | NC 002229.3 | I |
| 665 | Gallid herpesvirus 3 uid14103 | NC 002577.1 | I |
| 666 | Gammapapillomavirus HPV127 uid51741 | NC 014469.1 | I |
| 667 | Garlic latent virus uid15426 | NC 003557.1 | IV |
| 668 | Garlic virus A uid14735 | NC 003375.1 | IV |
| 669 | Garlic virus C uid14736 | NC 003376.1 | IV |
| 670 | Garlic virus E uid14834 | NC 004012.1 | IV |
| 671 | Garlic virus X uid14987 | NC 001800.1 | IV |
| 672 | GB virus A uid14647 | NC 001837.1 | IV |
| 673 | GB virus C uid15467 | NC 001710.1 | IV |
| 674 | Geobacillus phage GBSV1 uid17775 | NC 008376.2 | I |
| 675 | Geobacillus virus E2 uid19797 | NC 009552.2 | |
| 676 | Getah virus uid15049 | NC 006558.1 | IV |
| 677 | Giardia lamblia virus uid15018 | NC 003555.1 | III |
| 678 | Gibbon ape leukemia virus uid14657 | NC 001885.2 | VI |
| 679 | Gill associated virus uid28679 | NC 010306.1 | IV |
| 680 | Glossina pallidipes salivary gland hypertrophy virus uid28839 | NC 010356.1 | I |
| 681 | Goatpox virus Pellor uid14197 | NC 004003.1 | I |
| 682 | Goose circovirus uid14125 | NC 003054.1 | II |
| 683 | Goose hemorrhagic polyomavirus uid14286 | NC 004800.1 | I |
| 684 | Goose paramyxovirus SF02 uid14895 | NC 005036.1 | V |
| 685 | Goose parvovirus uid14098 | NC 001701.1 | II |
| 686 | Gossypium darwinii symptomless alphasatellite uid39593 | NC 013013.1 | NA |
| 687 | Gossypium darwinii symptomless virus uid33487 | NC 011804.1 | II |
| 688 | Gossypium davidsonii symptomless alphasatellite uid39589 | NC 013011.1 | NA |
| 689 | Gossypium mustilinum symptomless alphasatellite uid39591 | NC 013012.1 | NA |
| 690 | Grapevine Algerian latent virus uid32675 | NC 011535.1 | IV |
| 691 | Grapevine fanleaf virus satellite RNA uid14986 | NC 003203.1 | NA |
| 692 | Grapevine fleck virus uid15188 | NC 003347.1 | IV |
| 693 | Grapevine leafroll associated virus 10 uid33263 | NC 011702.1 | IV |
| 694 | Grapevine leafroll associated virus 2 uid15884 | NC 007448.1 | IV |

| | | | |
|---|---|---|---|
| 695 | Grapevine leafroll associated virus 3 uid14906 | NC 004667.1 | IV |
| 696 | Grapevine rootstock stem lesion associated virus uid14880 | NC 004724.1 | IV |
| 697 | Grapevine Syrah Virus 1 uid36515 | NC 012484.1 | IV |
| 698 | Grapevine virus A uid15086 | NC 003604.2 | IV |
| 699 | Grapevine virus B uid15083 | NC 003602.1 | IV |
| 700 | Grapevine virus E uid30853 | NC 011106.1 | IV |
| 701 | Gremmeniella abietina mitochondrial RNA virus S2 uid15229 | NC 006264.1 | IV |
| 702 | Gremmeniella abietina RNA virus L1 uid14824 | NC 003876.1 | III |
| 703 | Gremmeniella abietina RNA virus L2 uid15230 | NC 005965.1 | III |
| 704 | Gremmeniella abietina type B RNA virus XL1 uid16657 | NC 007920.1 | III |
| 705 | Ground squirrel hepatitis virus uid14070 | NC 001484.1 | VII |
| 706 | Groundnut rosette virus satellite RNA uid14429 | NC 002738.1 | NA |
| 707 | Groundnut rosette virus uid14762 | NC 003603.1 | IV |
| 708 | Gryllus bimaculatus nudivirus uid19181 | NC 009240.1 | I |
| 709 | Gull circovirus uid18019 | NC 008521.1 | II |
| 710 | H 1 parvovirus uid14578 | NC 001358.1 | II |
| 711 | Haemophilus phage Aaphi23 uid15228 | NC 004827.1 | I |
| 712 | Haemophilus phage HP1 uid14078 | NC 001697.1 | I |
| 713 | Haemophilus phage HP2 uid14231 | NC 003315.1 | I |
| 714 | Haloarcula hispanica pleomorphic virus 1 uid43589 | NC 013758.1 | I |
| 715 | Haloarcula phage SH1 uid15535 | NC 007217.1 | I |
| 716 | Halomonas phage phiHAP 1 uid28763 | NC 010342.1 | I |
| 717 | Halorubrum phage HF2 uid14147 | NC 003345.1 | I |
| 718 | Halorubrum pleomorphic virus 1 uid36677 | NC 012558.1 | II |
| 719 | Halovirus HF1 uid14294 | NC 004927.1 | I |
| 720 | Hamster polyomavirus uid14461 | NC 001663.1 | I |
| 721 | Helicobasidium mompa endornavirus 1 uid41437 | NC 013447.1 | III |
| 722 | Helicoverpa armigera granulovirus uid28275 | NC 010240.1 | I |
| 723 | Helicoverpa armigera multiple nucleopolyhedrovirus uid33003 | NC 011615.1 | I |
| 724 | Helicoverpa armigera NPV NNg1 uid32205 | NC 011354.1 | I |
| 725 | Helicoverpa armigera NPV uid14615 | NC 003094.2 | I |
| 726 | Helicoverpa armigera nucleopolyhedrovirus G4 uid14108 | NC 002654.2 | I |
| 727 | Helicoverpa zea SNPV uid14148 | NC 003349.1 | I |
| 728 | Heliothis virescens ascovirus 3e uid19151 | NC 009233.1 | I |
| 729 | Helleborus net necrosis virus uid33877 | NC 012038.1 | IV |
| 730 | Helminthosporium victoriae virus 190S uid14763 | NC 003607.2 | III |
| 731 | Hendra virus uid14911 | NC 001906.2 | V |
| 732 | Hepatitis A virus uid15308 | NC 001489.1 | IV |
| 733 | Hepatitis B virus uid15428 | NC 003977.1 | VII |
| 734 | Hepatitis C virus genotype 2 uid20937 | NC 009823.1 | IV |
| 735 | Hepatitis C virus genotype 3 uid20941 | NC 009824.1 | IV |
| 736 | Hepatitis C virus genotype 4 uid20933 | NC 009825.1 | IV |
| 737 | Hepatitis C virus genotype 5 uid20935 | NC 009826.1 | IV |
| 738 | Hepatitis C virus genotype 6 uid20939 | NC 009827.1 | IV |
| 739 | Hepatitis C virus uid15432 | NC 004102.1 | IV |
| 740 | Hepatitis delta virus uid15032 | NC 001653.2 | V |
| 741 | Hepatitis E virus uid15435 | NC 001434.1 | IV |
| 742 | Hepatitis GB virus B uid15364 | NC 001655.1 | IV |
| 743 | Heron hepatitis B virus uid15458 | NC 001486.1 | VII |
| 744 | Heterocapsa circularisquama RNA virus uid16157 | NC 007518.1 | IV |

| | | | |
|---|---|---|---|
| 745 | Heterosigma akashiwo RNA virus uid15425 | NC 005281.1 | IV |
| 746 | Hibiscus chlorotic ringspot virus uid15208 | NC 003608.1 | IV |
| 747 | Hibiscus latent Singapore virus uid17573 | NC 008310.1 | IV |
| 748 | Highlands J virus uid37281 | NC 012561.1 | IV |
| 749 | Himetobi P virus uid14801 | NC 003782.1 | IV |
| 750 | Hippeastrum latent virus uid32685 | NC 011540.1 | IV |
| 751 | Hirame rhabdovirus uid15132 | NC 005093.1 | V |
| 752 | His1 virus uid16650 | NC 007914.1 | I |
| 753 | His2 virus uid16651 | NC 007918.1 | I |
| 754 | HMO Astrovirus A uid41413 | NC 013443.1 | IV |
| 755 | Hollyhock leaf crumple virus satellite DNA uid14208 | NC 004092.1 | NA |
| 756 | Hollyhock leaf crumple virus uid14206 | NC 004071.1 | II |
| 757 | Homalodisca coagulata virus 1 uid16797 | NC 008029.1 | IV |
| 758 | Honeysuckle yellow vein beta Japan Fukui 2001  uid19601 | NC 009449.1 | NA |
| 759 | Honeysuckle yellow vein mosaic beta Japan Miyizaki 2001 uid19603 | NC 009450.1 | NA |
| 760 | Honeysuckle yellow vein mosaic disease associated satellite DNA beta uid19863 | NC 009571.1 | |
| 761 | Honeysuckle yellow vein mosaic virus Kagoshima  uid18657 | NC 008793.1 | II |
| 762 | Honeysuckle yellow vein mosaic virus satellite DNA beta uid14620 | NC 005052.1 | NA |
| 763 | Honeysuckle yellow vein mosaic virus uid14172 | NC 003609.1 | II |
| 764 | Honeysuckle yellow vein virus uid15224 | NC 005807.1 | II |
| 765 | Hop latent virus uid15373 | NC 002552.1 | IV |
| 766 | Hop mosaic virus uid29191 | NC 010538.1 | IV |
| 767 | Hordeum mosaic virus uid15064 | NC 005904.1 | IV |
| 768 | Horseradish curly top virus uid14100 | NC 002543.1 | II |
| 769 | Hosta virus X uid32693 | NC 011544.1 | IV |
| 770 | Human adenovirus 54 uid39353 | NC 012959.1 | I |
| 771 | Human adenovirus A uid14517 | NC 001460.1 | I |
| 772 | Human adenovirus B uid15150 | NC 011202.1 | I |
| 773 | Human adenovirus B uid31177 | NC 011203.1 | I |
| 774 | Human adenovirus C uid14518 | NC 001405.1 | I |
| 775 | Human adenovirus D uid14535 | NC 010956.1 | I |
| 776 | Human adenovirus E uid15152 | NC 003266.2 | I |
| 777 | Human adenovirus F uid14487 | NC 001454.1 | I |
| 778 | Human astrovirus uid15436 | NC 001943.1 | IV |
| 779 | Human bocavirus 2 uid33891 | NC 012042.1 | II |
| 780 | Human bocavirus 3 uid37291 | NC 012564.1 | II |
| 781 | Human bocavirus 4 uid38243 | NC 012729.2 | II |
| 782 | Human bocavirus uid15895 | NC 007455.1 | II |
| 783 | Human coronavirus 229E uid14913 | NC 002645.1 | IV |
| 784 | Human coronavirus HKU1 uid15139 | NC 006577.2 | IV |
| 785 | Human coronavirus NL63 uid14960 | NC 005831.2 | IV |
| 786 | Human coronavirus OC43 uid15438 | NC 005147.1 | IV |
| 787 | Human cosavirus A uid38497 | NC 012800.1 | IV |
| 788 | Human cosavirus B uid38499 | NC 012801.1 | IV |
| 789 | Human cosavirus D uid38501 | NC 012802.1 | IV |
| 790 | Human cosavirus E uid38493 | NC 012798.1 | IV |
| 791 | Human enteric coronavirus 4408 uid39335 | NC 012950.1 | IV |
| 792 | Human enterovirus 100 uid20973 | NC 009887.1 | IV |

| | | | |
|---|---|---|---|
| 793 | Human enterovirus 107 uid40905 | NC 013115.1 | IV |
| 794 | Human enterovirus 109 uid50755 | NC 014336.1 | IV |
| 795 | Human enterovirus 98 uid40161 | NC 013114.1 | IV |
| 796 | Human enterovirus A uid15445 | NC 001612.1 | IV |
| 797 | Human enterovirus B uid15321 | NC 001472.1 | IV |
| 798 | Human enterovirus C uid15296 | NC 001428.1 | IV |
| 799 | Human enterovirus D uid15297 | NC 001430.1 | IV |
| 800 | Human erythrovirus V9 uid14224 | NC 004295.1 | II |
| 801 | Human herpesvirus 1 uid15217 | NC 001806.1 | I |
| 802 | Human herpesvirus 2 uid15218 | NC 001798.1 | I |
| 803 | Human herpesvirus 3 uid15198 | NC 001348.1 | I |
| 804 | Human herpesvirus 4 type 2 uid20959 | NC 009334.1 | I |
| 805 | Human herpesvirus 4 uid14413 | NC 007605.1 | I |
| 806 | Human herpesvirus 5 uid14559 | NC 006273.2 | I |
| 807 | Human herpesvirus 6 uid14422 | NC 000898.1 | I |
| 808 | Human herpesvirus 6 uid14462 | NC 001664.2 | I |
| 809 | Human herpesvirus 7 uid14625 | NC 001716.2 | I |
| 810 | Human herpesvirus 8 uid14158 | NC 009333.1 | I |
| 811 | Human immunodeficiency virus 1 uid15476 | NC 001802.1 | VI |
| 812 | Human immunodeficiency virus 2 uid14991 | NC 001722.1 | VI |
| 813 | Human klassevirus 1 uid39553 | NC 012986.1 | IV |
| 814 | Human metapneumovirus uid15498 | NC 004148.2 | V |
| 815 | Human papillomavirus 1 uid15491 | NC 001356.1 | I |
| 816 | Human papillomavirus 18 uid15506 | NC 001357.1 | I |
| 817 | Human papillomavirus 2 uid15512 | NC 001352.1 | I |
| 818 | Human papillomavirus 54 uid15466 | NC 001676.1 | I |
| 819 | Human papillomavirus type 10 uid15504 | NC 001576.1 | I |
| 820 | Human papillomavirus type 101 uid17121 | NC 008189.1 | I |
| 821 | Human papillomavirus type 103 uid17119 | NC 008188.1 | I |
| 822 | Human papillomavirus type 108 uid34847 | NC 012213.1 | I |
| 823 | Human papillomavirus type 16 uid15505 | NC 001526.2 | I |
| 824 | Human papillomavirus type 26 uid15507 | NC 001583.1 | I |
| 825 | Human papillomavirus type 32 uid15508 | NC 001586.1 | I |
| 826 | Human papillomavirus type 34 uid15509 | NC 001587.1 | I |
| 827 | Human papillomavirus type 4 uid15492 | NC 001457.1 | I |
| 828 | Human papillomavirus type 41 uid15485 | NC 001354.1 | I |
| 829 | Human papillomavirus type 48 uid14027 | NC 001690.1 | I |
| 830 | Human papillomavirus type 49 uid15455 | NC 001591.1 | I |
| 831 | Human papillomavirus type 5 uid15511 | NC 001531.1 | I |
| 832 | Human papillomavirus type 50 uid14327 | NC 001691.1 | I |
| 833 | Human papillomavirus type 53 uid15510 | NC 001593.1 | I |
| 834 | Human papillomavirus type 60 uid14028 | NC 001693.1 | I |
| 835 | Human papillomavirus type 63 uid15486 | NC 001458.1 | I |
| 836 | Human papillomavirus type 6b uid15454 | NC 001355.1 | I |
| 837 | Human papillomavirus type 7 uid15450 | NC 001595.1 | I |
| 838 | Human papillomavirus type 88 uid28737 | NC 010329.1 | I |
| 839 | Human papillomavirus type 9 uid15456 | NC 001596.1 | I |
| 840 | Human papillomavirus type 90 uid15424 | NC 004104.1 | I |
| 841 | Human papillomavirus type 92 uid14406 | NC 004500.1 | I |
| 842 | Human papillomavirus type 96 uid15488 | NC 005134.2 | I |

| | | | |
|---|---|---|---|
| 843 | Human parainfluenza virus 1 uid14743 | NC 003461.1 | V |
| 844 | Human parainfluenza virus 2 uid15421 | NC 003443.1 | V |
| 845 | Human parainfluenza virus 3 uid14706 | NC 001796.2 | V |
| 846 | Human parechovirus uid15357 | NC 001897.1 | IV |
| 847 | Human parvovirus 4 uid15414 | NC 007018.1 | II |
| 848 | Human parvovirus B19 uid14090 | NC 000883.2 | II |
| 849 | Human respiratory syncytial virus uid15003 | NC 001781.1 | V |
| 850 | Human rhinovirus 14 uid15309 | NC 001490.1 | IV |
| 851 | Human rhinovirus A uid15330 | NC 001617.1 | IV |
| 852 | Human rhinovirus C uid27901 | NC 009996.1 | IV |
| 853 | Human T lymphotropic virus 1 uid15434 | NC 001436.1 | VI |
| 854 | Human T lymphotropic virus 2 uid14663 | NC 001488.1 | VI |
| 855 | Human T lymphotropic virus 4 uid33481 | NC 011800.1 | VI |
| 856 | Human TMEV like cardiovirus uid30053 | NC 010810.1 | IV |
| 857 | Hydrangea chlorotic mottle virus uid38689 | NC 012869.1 | IV |
| 858 | Hydrangea ringspot virus uid15151 | NC 006943.1 | IV |
| 859 | Hyperthermophilic Archaeal Virus 1 uid50363 | NC 014322.1 | I |
| 860 | Hyperthermophilic Archaeal Virus 2 uid50361 | NC 014321.1 | |
| 861 | Hyphantria cunea nucleopolyhedrovirus uid16343 | NC 007767.1 | I |
| 862 | Ictalurid herpesvirus 1 uid14018 | NC 001493.1 | I |
| 863 | Ilheus virus uid18845 | NC 009028.2 | IV |
| 864 | Imperata yellow mottle virus uid32677 | NC 011536.1 | IV |
| 865 | Indian citrus ringspot virus uid14716 | NC 003093.1 | IV |
| 866 | Infectious bronchitis virus uid15303 | NC 001451.1 | IV |
| 867 | Infectious flacherie virus uid14800 | NC 003781.1 | IV |
| 868 | Infectious hematopoietic necrosis virus uid14677 | NC 001652.1 | V |
| 869 | Infectious hypodermal and hematopoietic necrosis virus uid14436 | NC 002190.2 | II |
| 870 | Infectious spleen and kidney necrosis virus uid14600 | NC 003494.1 | I |
| 871 | Invertebrate iridescent virus 3 uid17099 | NC 008187.1 | I |
| 872 | Invertebrate iridescent virus 6 uid14124 | NC 003038.1 | I |
| 873 | Iodobacteriophage phiPLPE uid30965 | NC 011142.1 | I |
| 874 | Ipomoea yellow vein virus uid39615 | NC 013022.2 | II |
| 875 | Iranian maize mosaic nucleorhabdovirus uid32689 | NC 011542.1 | V |
| 876 | Israel acute paralysis virus of bees uid18855 | NC 009025.1 | IV |
| 877 | J virus uid15892 | NC 007454.1 | V |
| 878 | Jaagsiekte sheep retrovirus uid14665 | NC 001494.1 | VI |
| 879 | Japanese encephalitis virus uid15310 | NC 001437.1 | IV |
| 880 | Japanese iris necrotic ring virus uid15094 | NC 002187.1 | IV |
| 881 | Japanese yam mosaic virus uid15365 | NC 000947.1 | IV |
| 882 | Jatropha leaf curl virus uid31277 | NC 011268.1 | II |
| 883 | Jatropha yellow mosaic India virus uid32075 | NC 011309.1 | II |
| 884 | JC polyomavirus uid15477 | NC 001699.1 | I |
| 885 | Johnsongrass chlorotic stripe mosaic virus uid14904 | NC 005287.1 | IV |
| 886 | Johnsongrass mosaic virus uid15349 | NC 003606.1 | IV |
| 887 | Junonia coenia densovirus uid15423 | NC 004284.1 | II |
| 888 | Kakugo virus uid14957 | NC 005876.1 | IV |
| 889 | Kalanchoe latent virus uid39583 | NC 013006.1 | IV |
| 890 | Kalanchoe top spotting virus uid14236 | NC 004540.1 | VII |
| 891 | Kamiti River virus uid14896 | NC 005064.1 | IV |
| 892 | Karshi virus uid15149 | NC 006947.1 | IV |

| | | | |
|---|---|---|---|
| 893 | Kashmir bee virus uid14889 | NC 004807.1 | IV |
| 894 | Kedougou virus uid36617 | NC 012533.1 | IV |
| 895 | Kelp fly virus uid16201 | NC 007619.1 | IV |
| 896 | Kenaf leaf curl virus India Bahraich 2007  uid28991 | NC 010435.1 | II |
| 897 | Kennedya yellow mosaic virus uid14644 | NC 001746.1 | IV |
| 898 | KI polyomavirus uid19155 | NC 009238.1 | I |
| 899 | Klebsiella phage KP15 uid47333 | NC 014036.1 | I |
| 900 | Klebsiella phage KP32 uid42779 | NC 013647.1 | I |
| 901 | Klebsiella phage KP34 uid42781 | NC 013649.2 | I |
| 902 | Klebsiella phage phiKO2 uid14495 | NC 005857.1 | I |
| 903 | Kluyvera phage Kvp1 uid32673 | NC 011534.1 | I |
| 904 | Kokobera virus uid18843 | NC 009029.2 | IV |
| 905 | Konjac mosaic virus uid16643 | NC 007913.1 | IV |
| 906 | Kyuri green mottle mosaic virus uid15140 | NC 003610.1 | IV |
| 907 | Lactate dehydrogenase elevating virus uid14702 | NC 001639.1 | IV |
| 908 | Lactobacillus johnsonii prophage Lj771 uid28145 | NC 010179.2 | |
| 909 | Lactobacillus phage A2 uid14602 | NC 004112.1 | I |
| 910 | Lactobacillus phage KC5a uid16663 | NC 007924.1 | I |
| 911 | Lactobacillus phage Lb338 1 uid36611 | NC 012530.1 | I |
| 912 | Lactobacillus phage Lc Nu uid16114 | NC 007501.1 | I |
| 913 | Lactobacillus phage LL H uid19803 | NC 009554.1 | I |
| 914 | Lactobacillus phage LP65 uid14547 | NC 006565.1 | I |
| 915 | Lactobacillus phage Lrm1 uid30879 | NC 011104.1 | I |
| 916 | Lactobacillus phage Lv 1 uid33535 | NC 011801.1 | I |
| 917 | Lactobacillus phage phiadh uid14588 | NC 000896.1 | I |
| 918 | Lactobacillus phage phiAT3 uid14475 | NC 005893.1 | I |
| 919 | Lactobacillus phage phig1e uid14315 | NC 004305.1 | I |
| 920 | Lactobacillus phage phiJL 1 uid15156 | NC 006936.1 | I |
| 921 | Lactobacillus prophage Lj928 uid14350 | NC 005354.1 | I |
| 922 | Lactobacillus prophage Lj965 uid14351 | NC 005355.1 | I |
| 923 | Lactococcus phage 1706 uid29283 | NC 010576.1 | I |
| 924 | Lactococcus phage 712 uid17757 | NC 008370.1 | I |
| 925 | Lactococcus phage asccphi28 uid28985 | NC 010363.1 | I |
| 926 | Lactococcus phage bIBB29 uid30597 | NC 011046.1 | I |
| 927 | Lactococcus phage bIL170 uid14087 | NC 001909.1 | I |
| 928 | Lactococcus phage bIL285 uid14111 | NC 002666.1 | I |
| 929 | Lactococcus phage bIL286 uid14397 | NC 002667.1 | I |
| 930 | Lactococcus phage bIL309 uid14338 | NC 002668.1 | I |
| 931 | Lactococcus phage bIL310 uid14112 | NC 002669.1 | I |
| 932 | Lactococcus phage bIL311 uid14139 | NC 002670.1 | I |
| 933 | Lactococcus phage bIL312 uid14113 | NC 002671.1 | I |
| 934 | Lactococcus phage bIL67 uid32321 | NC 001629.1 | I |
| 935 | Lactococcus phage BK5 T uid15244 | NC 002796.1 | I |
| 936 | Lactococcus phage c2 uid14029 | NC 001706.1 | I |
| 937 | Lactococcus phage jj50 uid17759 | NC 008371.1 | I |
| 938 | Lactococcus phage KSY1 uid20783 | NC 009817.1 | I |
| 939 | Lactococcus phage P008 uid17737 | NC 008363.1 | I |
| 940 | Lactococcus phage P087 uid37887 | NC 012663.1 | I |
| 941 | Lactococcus phage P335 sensu lato uid14281 | NC 004746.1 | I |
| 942 | Lactococcus phage phiLC3 uid14362 | NC 005822.1 | I |

| | | | |
|---|---|---|---|
| 943 | Lactococcus phage Q54 uid17739 | NC 008364.1 | I |
| 944 | Lactococcus phage r1t uid14225 | NC 004302.1 | I |
| 945 | Lactococcus phage sk1 uid14096 | NC 001835.1 | I |
| 946 | Lactococcus phage TP901 1 uid14116 | NC 002747.1 | I |
| 947 | Lactococcus phage Tuc2009 uid14131 | NC 002703.1 | I |
| 948 | Lactococcus phage ul36 uid14331 | NC 004066.1 | I |
| 949 | Lake Victoria marburgvirus uid15199 | NC 001608.3 | V |
| 950 | Lamium leaf distortion associated virus uid29877 | NC 010737.1 | VII |
| 951 | Langat virus uid15370 | NC 003690.1 | IV |
| 952 | Leek white stripe virus uid15082 | NC 001822.1 | IV |
| 953 | Leek yellow stripe virus uid15184 | NC 004011.1 | IV |
| 954 | Leishmania RNA virus 1 4 uid14761 | NC 003601.1 | III |
| 955 | Leishmania RNA virus 1 uid14666 | NC 002063.1 | III |
| 956 | Leishmania RNA virus 2 1 uid14696 | NC 002064.1 | III |
| 957 | Lettuce mosaic virus uid15342 | NC 003605.1 | IV |
| 958 | Lettuce necrotic yellows virus uid16236 | NC 007642.1 | V |
| 959 | Lettuce virus X uid30177 | NC 010832.1 | IV |
| 960 | Lettuce yellow mottle virus uid32669 | NC 011532.1 | V |
| 961 | Leucania separata nuclear polyhedrosis virus uid17669 | NC 008348.1 | I |
| 962 | Leucas zeylanica yellow vein virus satellite DNA beta uid41305 | NC 013424.1 | NA |
| 963 | Ligustrum necrotic ringspot virus uid28681 | NC 010305.1 | IV |
| 964 | Lily mottle virus uid15495 | NC 005288.1 | IV |
| 965 | Lily symptomless virus uid15015 | NC 005138.1 | IV |
| 966 | Lily virus X uid15494 | NC 007192.1 | IV |
| 967 | Lindernia anagallis yellow vein virus satellite DNA beta uid19831 | NC 009561.1 | NA |
| 968 | Lindernia anagallis yellow vein virus uid19777 | NC 009550.1 | II |
| 969 | Lisianthus necrosis virus uid16737 | NC 007983.1 | IV |
| 970 | Listeria phage 2389 uid14142 | NC 003291.2 | I |
| 971 | Listeria phage A006 uid20801 | NC 009815.1 | I |
| 972 | Listeria phage A118 uid14589 | NC 003216.1 | I |
| 973 | Listeria phage A500 uid20791 | NC 009810.1 | I |
| 974 | Listeria phage A511 uid20793 | NC 009811.2 | I |
| 975 | Listeria phage B025 uid20795 | NC 009812.1 | I |
| 976 | Listeria phage B054 uid20797 | NC 009813.1 | I |
| 977 | Listeria phage P35 uid20799 | NC 009814.1 | I |
| 978 | Listeria phage P40 uid32073 | NC 011308.1 | I |
| 979 | Listonella phage phiHSIC uid15173 | NC 006953.1 | I |
| 980 | Little cherry virus 1 uid15346 | NC 001836.1 | IV |
| 981 | Little cherry virus 2 uid15062 | NC 005065.1 | IV |
| 982 | Ljungan virus uid15401 | NC 003976.2 | IV |
| 983 | Lolium latent virus uid28971 | NC 010434.1 | IV |
| 984 | Louis encephalitis virus uid16150 | NC 007580.2 | IV |
| 985 | Louping ill virus uid15343 | NC 001809.1 | IV |
| 986 | Lucerne transient streak virus satellite RNA uid14501 | NC 003798.1 | NA |
| 987 | Lucerne transient streak virus uid15337 | NC 001696.1 | IV |
| 988 | Lucky bamboo bacilliform virus uid19855 | NC 009568.1 | VII |
| 989 | Ludwigia leaf distortion betasatellite India Amadalavalasa Hibiscus 2007 uid29233 | NC 010569.1 | |
| 990 | Ludwigia yellow vein virus associated DNA beta uid15561 | NC 007212.1 | NA |
| 991 | Ludwigia yellow vein virus uid15559 | NC 007210.2 | II |

| | | | |
|---|---|---|---|
| 992 | Luffa begomovirus associated DNA beta uid16795 | NC 008031.1 | NA |
| 993 | Luffa puckering and leaf distortion associated DNA beta uid15779 | NC 007459.1 | NA |
| 994 | LuIII virus uid14278 | NC 004713.1 | II |
| 995 | Lumpy skin disease virus NI 2490 uid14122 | NC 003027.1 | I |
| 996 | Lymantria dispar MNPV uid14390 | NC 001973.1 | I |
| 997 | Lymantria xylina MNPV uid46671 | NC 013953.1 | I |
| 998 | Lymphocystis disease virus isolate China uid14472 | NC 005902.1 | I |
| 999 | Lymphocystis disease virus 1 uid14081 | NC 001824.1 | I |
| 1000 | Macacine herpesvirus 1 uid14489 | NC 004812.1 | I |
| 1001 | Macacine herpesvirus 3 uid14468 | NC 006150.1 | I |
| 1002 | Macacine herpesvirus 4 uid14467 | NC 006146.1 | I |
| 1003 | Macacine herpesvirus 5 uid14423 | NC 003401.1 | I |
| 1004 | Macaque simian foamy virus uid30115 | NC 010819.1 | VI |
| 1005 | Magnaporthe oryzae virus 1 uid15041 | NC 006367.1 | III |
| 1006 | Magnaporthe oryzae virus 2 uid28297 | NC 010246.1 | III |
| 1007 | Maize chlorotic dwarf virus uid15345 | NC 003626.1 | IV |
| 1008 | Maize chlorotic mottle virus uid15117 | NC 003627.1 | IV |
| 1009 | Maize dwarf mosaic virus uid15355 | NC 003377.1 | IV |
| 1010 | Maize fine streak virus uid15216 | NC 005974.1 | V |
| 1011 | Maize mosaic virus uid14920 | NC 005975.1 | V |
| 1012 | Maize necrotic streak virus uid16323 | NC 007729.1 | IV |
| 1013 | Maize rayado fino virus uid15381 | NC 002786.1 | IV |
| 1014 | Maize streak virus uid14577 | NC 001346.1 | II |
| 1015 | Maize white line mosaic satellite virus uid14770 | NC 003631.1 | NA |
| 1016 | Maize white line mosaic virus uid19755 | NC 009533.1 | IV |
| 1017 | Malachra yellow vein mosaic virus associated satellite DNA beta uid28727 | NC 010328.1 | NA |
| 1018 | Malvastrum leaf curl Guangdong virus uid17593 | NC 008316.1 | II |
| 1019 | Malvastrum leaf curl virus associated defective DNA beta uid16320 | NC 007725.1 | NA |
| 1020 | Malvastrum leaf curl virus uid16325 | NC 007724.1 | II |
| 1021 | Malvastrum yellow mosaic virus associated DNA 1 uid18129 | NC 008561.1 | NA |
| 1022 | Malvastrum yellow mosaic virus satellite DNA beta uid18133 | NC 008560.1 | NA |
| 1023 | Malvastrum yellow mosaic virus uid18131 | NC 008559.1 | II |
| 1024 | Malvastrum yellow vein Baoshan virus uid37891 | NC 012665.1 | II |
| 1025 | Malvastrum yellow vein virus satellite DNA beta uid15317 | NC 004733.1 | NA |
| 1026 | Malvastrum yellow vein virus uid14252 | NC 004634.1 | II |
| 1027 | Malvastrum yellow vein Yunnan virus satellite DNA beta uid14567 | NC 006632.1 | NA |
| 1028 | Malvastrum yellow vein Yunnan virus uid15231 | NC 006631.1 | II |
| 1029 | Mamestra configurata NPV A uid14168 | NC 003529.1 | I |
| 1030 | Mamestra configurata NPV B uid15128 | NC 004117.1 | I |
| 1031 | Mannheimia phage phiMHaA1 uid17103 | NC 008201.1 | I |
| 1032 | Mapuera virus uid19651 | NC 009489.1 | V |
| 1033 | Maracuja mosaic virus uid18531 | NC 008716.1 | IV |
| 1034 | Marine RNA virus JP A uid20649 | NC 009757.1 | IV |
| 1035 | Marine RNA virus JP B uid20651 | NC 009758.1 | IV |
| 1036 | Marine RNA virus SOG uid20647 | NC 009756.1 | IV |
| 1037 | Marseillevirus uid43573 | NC 013756.1 | I |
| 1038 | Maruca vitrata MNPV uid18533 | NC 008725.1 | I |
| 1039 | Mason Pfizer monkey virus uid14683 | NC 001550.1 | VI |
| 1040 | Mastomys coucha papillomavirus 2 uid18011 | NC 008519.1 | I |

| | | | |
|---|---|---|---|
| 1041 | Mastomys natalensis papillomavirus uid14022 | NC 001605.1 | I |
| 1042 | Mayaro virus uid15392 | NC 003417.1 | IV |
| 1043 | Measles virus uid15025 | NC 001498.1 | V |
| 1044 | Melanoplus sanguinipes entomopoxvirus uid14042 | NC 001993.1 | I |
| 1045 | Meleagrid herpesvirus 1 uid14106 | NC 002641.1 | I |
| 1046 | Melon aphid borne yellows virus uid30049 | NC 010809.1 | IV |
| 1047 | Melon chlorotic mosaic virus associated alphasatellite uid51413 | NC 014379.1 | NA |
| 1048 | Melon necrotic spot virus uid15502 | NC 001504.1 | IV |
| 1049 | Menangle virus uid16205 | NC 007620.1 | V |
| 1050 | Merkel cell polyomavirus uid28509 | NC 010277.1 | I |
| 1051 | Mesta yellow vein mosaic Bahraich virus uid30083 | NC 010818.1 | II |
| 1052 | Mesta yellow vein mosaic virus associated DNA beta uid21015 | NC 009903.1 | NA |
| 1053 | Mesta yellow vein mosaic virus uid18967 | NC 009088.1 | II |
| 1054 | Methanobacterium phage psiM2 uid14160 | NC 001902.1 | I |
| 1055 | Methanothermobacter phage psiM100 uid14289 | NC 002628.2 | I |
| 1056 | Microbacterium phage Min1 uid19961 | NC 009603.1 | I |
| 1057 | Microcystis phage Ma LMM01 uid18127 | NC 008562.1 | I |
| 1058 | Midway virus uid38097 | NC 012702.1 | V |
| 1059 | Mimosa yellow leaf curl virus associated DNA 1 uid19817 | NC 009564.1 | NA |
| 1060 | Mimosa yellow leaf curl virus satellite DNA beta uid19821 | NC 009556.1 | NA |
| 1061 | Mimosa yellow leaf curl virus uid19781 | NC 009546.1 | II |
| 1062 | Mink astrovirus uid14897 | NC 004579.1 | IV |
| 1063 | Mint virus 1 uid15210 | NC 006944.1 | IV |
| 1064 | Mint virus X uid15160 | NC 006948.1 | IV |
| 1065 | Minute virus of mice uid14019 | NC 001510.1 | II |
| 1066 | Mirabilis mosaic virus uid14393 | NC 004036.1 | VII |
| 1067 | Miscanthus streak virus uid14151 | NC 003379.1 | II |
| 1068 | Modoc virus uid15393 | NC 003635.1 | IV |
| 1069 | Mokola virus uid15013 | NC 006429.1 | V |
| 1070 | Molluscum contagiosum virus subtype 1 uid14328 | NC 001731.1 | I |
| 1071 | Moloney murine leukemia virus uid15030 | NC 001501.1 | VI |
| 1072 | Moloney murine sarcoma virus uid14721 | NC 001502.1 | VI |
| 1073 | Monkeypox virus Zaire 96 I 16 uid15142 | NC 003310.1 | I |
| 1074 | Montana myotis leukoencephalitis virus uid15402 | NC 004119.1 | IV |
| 1075 | Morganella phage MmP1 uid30793 | NC 011085.2 | I |
| 1076 | Moroccan watermelon mosaic virus uid27897 | NC 009995.1 | IV |
| 1077 | Mossman virus uid14915 | NC 005339.1 | V |
| 1078 | Mouse mammary tumor virus uid14435 | NC 001503.1 | VI |
| 1079 | Mouse parvovirus 1 uid14325 | NC 001630.1 | II |
| 1080 | Mouse parvovirus 2 uid17125 | NC 008186.1 | II |
| 1081 | Mouse parvovirus 3 uid17123 | NC 008185.1 | II |
| 1082 | Mouse parvovirus 4a uid33009 | NC 011619.1 | II |
| 1083 | Mouse parvovirus 5a uid33007 | NC 011618.1 | II |
| 1084 | Mulard duck circovirus uid14619 | NC 005053.1 | II |
| 1085 | Mumps virus uid15059 | NC 002200.1 | V |
| 1086 | Munia coronavirus HKU13 3514 uid32703 | NC 011550.1 | IV |
| 1087 | Murid herpesvirus 1 uid15181 | NC 004065.1 | I |
| 1088 | Murid herpesvirus 2 uid14419 | NC 002512.2 | I |
| 1089 | Murid herpesvirus 4 uid14458 | NC 001826.2 | I |
| 1090 | Murine adenovirus 3 uid37713 | NC 012584.1 | I |

| | | | |
|---|---|---|---|
| 1091 | Murine adenovirus A uid14519 | NC 000942.1 | I |
| 1092 | Murine hepatitis virus A59 uid15350 | NC 001846.1 | IV |
| 1093 | Murine hepatitis virus JHM uid15138 | NC 006852.1 | IV |
| 1094 | Murine norovirus 1 uid17577 | NC 008311.1 | IV |
| 1095 | Murine osteosarcoma virus uid14655 | NC 001506.1 | VI |
| 1096 | Murine pneumotropic virus uid14071 | NC 001505.2 | I |
| 1097 | Murine polyomavirus uid15489 | NC 001515.1 | I |
| 1098 | Murine type C retrovirus uid15204 | NC 001702.1 | VI |
| 1099 | Murray Valley encephalitis virus uid15430 | NC 000943.1 | IV |
| 1100 | Mus musculus papillomavirus type 1 uid50561 | NC 014326.1 | I |
| 1101 | Musca domestica salivary gland hypertrophy virus uid29631 | NC 010671.1 | I |
| 1102 | Muscovy duck circovirus uid14543 | NC 006561.1 | II |
| 1103 | Muscovy duck parvovirus uid14425 | NC 006147.2 | II |
| 1104 | Mushroom bacilliform virus uid14676 | NC 001633.1 | IV |
| 1105 | Mycobacterium phage 244 uid17115 | NC 008194.1 | I |
| 1106 | Mycobacterium phage Adjutor uid29919 | NC 010763.1 | I |
| 1107 | Mycobacterium phage Angel uid38461 | NC 012788.1 | I |
| 1108 | Mycobacterium phage Angelica uid51667 | NC 014458.1 | I |
| 1109 | Mycobacterium phage Ardmore uid46607 | NC 013936.1 | I |
| 1110 | Mycobacterium phage Barnyard uid14274 | NC 004689.1 | I |
| 1111 | Mycobacterium phage Bethlehem uid20945 | NC 009878.1 | I |
| 1112 | Mycobacterium phage Boomer uid30693 | NC 011054.1 | I |
| 1113 | Mycobacterium phage BPs uid29917 | NC 010762.1 | I |
| 1114 | Mycobacterium phage Brujita uid32005 | NC 011291.1 | I |
| 1115 | Mycobacterium phage Butterscotch uid32007 | NC 011286.1 | I |
| 1116 | Mycobacterium phage Bxb1 uid14109 | NC 002656.1 | I |
| 1117 | Mycobacterium phage Bxz1 uid14309 | NC 004687.1 | I |
| 1118 | Mycobacterium phage Bxz2 uid14275 | NC 004682.1 | I |
| 1119 | Mycobacterium phage Cali uid31291 | NC 011271.1 | I |
| 1120 | Mycobacterium phage Catera uid17141 | NC 008207.1 | I |
| 1121 | Mycobacterium phage Chah uid32021 | NC 011284.1 | I |
| 1122 | Mycobacterium phage Che12 uid17143 | NC 008203.1 | I |
| 1123 | Mycobacterium phage Che8 uid14394 | NC 004680.1 | I |
| 1124 | Mycobacterium phage Che9c uid14271 | NC 004683.1 | I |
| 1125 | Mycobacterium phage Che9d uid14339 | NC 004686.1 | I |
| 1126 | Mycobacterium phage Cjw1 uid14270 | NC 004681.1 | I |
| 1127 | Mycobacterium phage Cooper uid17145 | NC 008195.1 | I |
| 1128 | Mycobacterium phage Corndog uid14272 | NC 004685.1 | I |
| 1129 | Mycobacterium phage CrimD uid51669 | NC 014459.1 | I |
| 1130 | Mycobacterium phage D29 uid14203 | NC 001900.1 | I |
| 1131 | Mycobacterium phage DD5 uid30513 | NC 011022.1 | I |
| 1132 | Mycobacterium phage ET08 uid42783 | NC 013650.1 | I |
| 1133 | Mycobacterium phage Fruitloop uid32013 | NC 011288.1 | I |
| 1134 | Mycobacterium phage Giles uid27907 | NC 009993.2 | I |
| 1135 | Mycobacterium phage Gumball uid32009 | NC 011290.1 | I |
| 1136 | Mycobacterium phage Halo uid17147 | NC 008202.1 | I |
| 1137 | Mycobacterium phage Jasper uid30515 | NC 011020.1 | I |
| 1138 | Mycobacterium phage KBG uid30517 | NC 011019.1 | I |
| 1139 | Mycobacterium phage Konstantine uid32015 | NC 011292.1 | I |
| 1140 | Mycobacterium phage Kostya uid30695 | NC 011056.1 | I |

| | | | |
|---|---|---|---|
| 1141 | Mycobacterium phage L5 uid14459 | NC 001335.1 | I |
| 1142 | Mycobacterium phage LeBron uid51673 | NC 014461.1 | I |
| 1143 | Mycobacterium phage Llij uid17149 | NC 008196.1 | I |
| 1144 | Mycobacterium phage Lockley uid30519 | NC 011021.1 | I |
| 1145 | Mycobacterium phage Myrna uid31279 | NC 011273.1 | I |
| 1146 | Mycobacterium phage Nigel uid30609 | NC 011044.1 | I |
| 1147 | Mycobacterium phage Omega uid14273 | NC 004688.1 | I |
| 1148 | Mycobacterium phage Orion uid17151 | NC 008197.1 | I |
| 1149 | Mycobacterium phage Pacc40 uid32017 | NC 011287.1 | I |
| 1150 | Mycobacterium phage PBI1 uid17165 | NC 008198.1 | I |
| 1151 | Mycobacterium phage Peaches uid42939 | NC 013694.1 | I |
| 1152 | Mycobacterium phage PG1 uid14357 | NC 005259.1 | I |
| 1153 | Mycobacterium phage Phaedrus uid30697 | NC 011057.1 | I |
| 1154 | Mycobacterium phage Phlyer uid33871 | NC 012027.1 | I |
| 1155 | Mycobacterium phage Pipefish uid17171 | NC 008199.1 | I |
| 1156 | Mycobacterium phage PLot uid17167 | NC 008200.1 | I |
| 1157 | Mycobacterium phage PMC uid17169 | NC 008205.1 | I |
| 1158 | Mycobacterium phage Porky uid30699 | NC 011055.1 | I |
| 1159 | Mycobacterium phage Predator uid30611 | NC 011039.1 | I |
| 1160 | Mycobacterium phage Pukovnik uid30521 | NC 011023.1 | I |
| 1161 | Mycobacterium phage Qyrzula uid17173 | NC 008204.1 | I |
| 1162 | Mycobacterium phage Ramsey uid32019 | NC 011289.1 | I |
| 1163 | Mycobacterium phage Rizal uid31281 | NC 011272.1 | I |
| 1164 | Mycobacterium phage Rosebush uid14304 | NC 004684.1 | I |
| 1165 | Mycobacterium phage ScottMcG uid31283 | NC 011269.1 | I |
| 1166 | Mycobacterium phage Solon uid31287 | NC 011267.1 | I |
| 1167 | Mycobacterium phage Spud uid31285 | NC 011270.1 | I |
| 1168 | Mycobacterium phage TM4 uid14154 | NC 003387.1 | I |
| 1169 | Mycobacterium phage Troll4 uid32011 | NC 011285.1 | I |
| 1170 | Mycobacterium phage Tweety uid20787 | NC 009820.1 | I |
| 1171 | Mycobacterium phage U2 uid20943 | NC 009877.1 | I |
| 1172 | Mycobacterium phage Wildcat uid17175 | NC 008206.1 | I |
| 1173 | Mycoplasma phage MAV1 uid14395 | NC 001942.1 | I |
| 1174 | Mycoplasma phage P1 uid14136 | NC 002515.1 | I |
| 1175 | Mycoplasma phage phiMFV1 uid14387 | NC 005964.1 | |
| 1176 | Myotis polyomavirus VM 2008 uid32077 | NC 011310.1 | I |
| 1177 | Mythimna loreyi densovirus uid14346 | NC 005341.1 | II |
| 1178 | Myxococcus phage Mx8 uid14391 | NC 003085.1 | I |
| 1179 | Myxoma virus uid14396 | NC 001132.2 | I |
| 1180 | Myzus persicae densovirus uid14299 | NC 005040.1 | II |
| 1181 | Nanovirus like particle uid14386 | NC 005954.1 | NA |
| 1182 | Narcissus common latent virus uid17373 | NC 008266.1 | IV |
| 1183 | Narcissus degeneration virus uid18729 | NC 008824.1 | IV |
| 1184 | Narcissus mosaic virus uid14660 | NC 001441.1 | IV |
| 1185 | Narcissus symptomless virus uid18071 | NC 008552.1 | IV |
| 1186 | Narcissus yellow stripe virus uid32687 | NC 011541.1 | IV |
| 1187 | Natrialba phage PhiCh1 uid14207 | NC 004084.1 | I |
| 1188 | Nemesia ring necrosis virus uid32681 | NC 011538.1 | IV |
| 1189 | Neodiprion abietis NPV uid17361 | NC 008252.1 | I |
| 1190 | Neodiprion lecontii NPV uid14617 | NC 005906.1 | I |

| | | | |
|---|---|---|---|
| 1191 | Neodiprion sertifer NPV uid14383 | NC 005905.1 | I |
| 1192 | Nerine virus X uid16257 | NC 007679.1 | IV |
| 1193 | Newbury agent 1 uid16653 | NC 007916.1 | IV |
| 1194 | Newcastle disease virus B1 uid15433 | NC 002617.1 | V |
| 1195 | Ngaingan virus uid46715 | NC 013955.1 | V |
| 1196 | Nipah virus uid15443 | NC 002728.1 | V |
| 1197 | Nootka lupine vein clearing virus uid18853 | NC 009017.1 | IV |
| 1198 | Nora virus uid16656 | NC 007919.3 | IV |
| 1199 | Northern cereal mosaic virus uid14984 | NC 002251.1 | V |
| 1200 | Norwalk virus uid15520 | NC 001959.2 | IV |
| 1201 | Nudaurelia capensis beta virus uid14982 | NC 001990.1 | IV |
| 1202 | Nyamanini virus uid38109 | NC 012703.1 | V |
| 1203 | O nyong nyong virus uid15311 | NC 001512.1 | IV |
| 1204 | Oat blue dwarf virus uid15341 | NC 001793.1 | IV |
| 1205 | Oat chlorotic stunt virus uid15081 | NC 003633.1 | IV |
| 1206 | Oat dwarf virus uid30037 | NC 010799.1 | II |
| 1207 | Oat necrotic mottle virus uid14899 | NC 005136.1 | IV |
| 1208 | Obuda pepper virus uid14817 | NC 003852.1 | IV |
| 1209 | Odontoglossum ringspot virus uid15201 | NC 001728.1 | IV |
| 1210 | Okra leaf curl disease associated DNA 1 uid29397 | NC 010620.1 | NA |
| 1211 | Okra leaf curl Mali virus satellite DNA beta uid20323 | NC 009731.1 | NA |
| 1212 | Okra leaf curl virus satellite DNA beta uid14209 | NC 004093.1 | NA |
| 1213 | Okra leaf curl virus uid39605 | NC 013017.1 | II |
| 1214 | Okra mosaic virus uid19761 | NC 009532.1 | IV |
| 1215 | Okra yellow crinkle virus uid17807 | NC 008377.1 | II |
| 1216 | Okra yellow vein disease associated sequence uid14443 | NC 005051.1 | NA |
| 1217 | Okra yellow vein mosaic virus uid14266 | NC 004673.1 | II |
| 1218 | Old World harvest mouse papillomavirus uid18259 | NC 008582.1 | I |
| 1219 | Olive latent virus 1 uid15084 | NC 001721.1 | IV |
| 1220 | Olive latent virus 3 uid46223 | NC 013920.1 | IV |
| 1221 | Olive mild mosaic virus uid15159 | NC 006939.1 | IV |
| 1222 | Omsk hemorrhagic fever virus uid14995 | NC 005062.1 | IV |
| 1223 | Onion yellow dwarf virus uid15407 | NC 005029.1 | IV |
| 1224 | Ononis yellow mosaic virus uid14669 | NC 001513.1 | IV |
| 1225 | Ophiostoma mitovirus 3a uid14839 | NC 004049.1 | IV |
| 1226 | Ophiostoma mitovirus 4 uid14842 | NC 004052.1 | IV |
| 1227 | Ophiostoma mitovirus 5 uid14843 | NC 004053.1 | IV |
| 1228 | Ophiostoma mitovirus 6 uid14844 | NC 004054.1 | IV |
| 1229 | Opuntia virus X uid14956 | NC 006060.1 | IV |
| 1230 | Orangutan polyomavirus uid41471 | NC 013439.1 | I |
| 1231 | Orf virus uid14464 | NC 005336.1 | I |
| 1232 | Orgyia leucostigma NPV uid28501 | NC 010276.1 | I |
| 1233 | Orgyia pseudotsugata MNPV uid14084 | NC 001875.2 | I |
| 1234 | Oryctes rhinoceros virus uid32781 | NC 011588.1 | I |
| 1235 | Oryza rufipogon endornavirus uid16238 | NC 007649.1 | III |
| 1236 | Oryza sativa endornavirus uid16239 | NC 007647.1 | III |
| 1237 | Ostreid herpesvirus 1 uid14552 | NC 005881.1 | I |
| 1238 | Ostreococcus tauri virus 1 uid40907 | NC 013288.1 | I |
| 1239 | Ostreococcus virus OsV5 uid28159 | NC 010191.1 | I |
| 1240 | Ovine adenovirus A uid14497 | NC 002513.1 | I |

| | | | |
|---|---|---|---|
| 1241 | Ovine adenovirus D uid14198 | NC 004037.2 | I |
| 1242 | Ovine astrovirus uid15095 | NC 002469.1 | IV |
| 1243 | Ovine enzootic nasal tumour virus uid15410 | NC 007015.1 | VI |
| 1244 | Ovine herpesvirus 2 uid16234 | NC 007646.1 | I |
| 1245 | Ovine lentivirus uid14668 | NC 001511.1 | VI |
| 1246 | Ovine papillomavirus 1 uid15460 | NC 001789.1 | I |
| 1247 | Oyster mushroom spherical virus uid14951 | NC 004560.1 | IV |
| 1248 | Panax virus Y uid49715 | NC 014252.1 | IV |
| 1249 | Panicum mosaic satellite virus uid14816 | NC 003847.1 | NA |
| 1250 | Panicum mosaic virus uid14979 | NC 002598.1 | IV |
| 1251 | Panicum streak virus uid14076 | NC 001647.1 | II |
| 1252 | Panine herpesvirus 2 uid14404 | NC 003521.1 | I |
| 1253 | Papaya leaf curl China virus satellite DNA beta uid19819 | NC 009555.1 | NA |
| 1254 | Papaya leaf curl China virus uid14536 | NC 005321.1 | II |
| 1255 | Papaya leaf curl Guangdong virus uid14537 | NC 005844.1 | II |
| 1256 | Papaya leaf curl virus associated DNA beta uid14448 | NC 004706.1 | NA |
| 1257 | Papaya leaf curl virus uid14213 | NC 004147.1 | II |
| 1258 | Papaya leaf distortion mosaic virus uid15405 | NC 005028.1 | IV |
| 1259 | Papaya mosaic virus uid14700 | NC 001748.1 | IV |
| 1260 | Papaya ringspot virus uid15289 | NC 001785.1 | IV |
| 1261 | Papiine herpesvirus 2 uid16246 | NC 007653.1 | I |
| 1262 | Paprika mild mottle virus uid14935 | NC 004106.1 | IV |
| 1263 | Parainfluenza virus 5 uid15014 | NC 006430.1 | V |
| 1264 | Paramecium bursaria Chlorella virus 1 uid14564 | NC 000852.5 | I |
| 1265 | Paramecium bursaria Chlorella virus AR158 uid20991 | NC 009899.1 | I |
| 1266 | Paramecium bursaria Chlorella virus FR483 uid18305 | NC 008603.1 | I |
| 1267 | Paramecium bursaria Chlorella virus NY2A uid20989 | NC 009898.1 | I |
| 1268 | Parsnip yellow fleck virus uid15299 | NC 003628.1 | IV |
| 1269 | Passiflora latent carlavirus uid17487 | NC 008292.1 | IV |
| 1270 | Pasteurella phage F108 uid17113 | NC 008193.1 | I |
| 1271 | Pea enation mosaic virus 1 uid14769 | NC 003629.1 | IV |
| 1272 | Pea enation mosaic virus 2 uid14818 | NC 003853.1 | IV |
| 1273 | Pea enation mosaic virus satellite RNA uid14432 | NC 003854.1 | NA |
| 1274 | Pea seed borne mosaic virus uid15295 | NC 001671.1 | IV |
| 1275 | Pea stem necrosis virus uid14894 | NC 004995.1 | IV |
| 1276 | Peach chlorotic mottle virus uid20977 | NC 009892.1 | IV |
| 1277 | Peach mosaic virus uid32727 | NC 011552.1 | IV |
| 1278 | Peanut chlorotic streak virus uid14388 | NC 001634.1 | VII |
| 1279 | Peanut mottle virus uid15352 | NC 002600.1 | IV |
| 1280 | Peanut stunt virus satellite RNA uid14502 | NC 003855.1 | NA |
| 1281 | Pear latent virus uid14879 | NC 004723.1 | IV |
| 1282 | Pedilanthus leaf curl virus uid34665 | NC 012118.1 | II |
| 1283 | Pelargonium chlorotic ring pattern virus uid14922 | NC 005985.1 | IV |
| 1284 | Pelargonium flower break virus uid14928 | NC 005286.1 | IV |
| 1285 | Pelargonium line pattern virus uid15413 | NC 007017.1 | IV |
| 1286 | Pelargonium necrotic spot virus uid15214 | NC 005285.1 | IV |
| 1287 | Pelargonium vein banding virus uid40631 | NC 013262.1 | VII |
| 1288 | Penaeid shrimp infectious myonecrosis virus uid16652 | NC 007915.1 | III |
| 1289 | Penaeus merguiensis densovirus uid15556 | NC 007218.1 | II |
| 1290 | Penaeus monodon hepatopancreatic parvovirus uid32695 | NC 011545.2 | II |

| | | | |
|---|---|---|---|
| 1291 | Pennisetum mosaic virus uid15447 | NC 007147.1 | IV |
| 1292 | Penstemon ringspot virus uid17489 | NC 008295.1 | IV |
| 1293 | Pepino mosaic virus uid15125 | NC 004067.1 | IV |
| 1294 | Pepper curly top virus uid19745 | NC 009518.1 | II |
| 1295 | Pepper leaf curl Bangladesh virus uid14218 | NC 004192.1 | II |
| 1296 | Pepper leaf curl virus satellite DNA beta uid28283 | NC 010235.1 | NA |
| 1297 | Pepper leaf curl virus uid14046 | NC 000882.1 | II |
| 1298 | Pepper leaf curl Yunnan virus satellite DNA beta uid29415 | NC 010619.1 | NA |
| 1299 | Pepper leaf curl Yunnan virus YN323 uid29413 | NC 010618.1 | II |
| 1300 | Pepper mild mottle virus uid15148 | NC 003630.1 | IV |
| 1301 | Pepper mottle virus uid15312 | NC 001517.1 | IV |
| 1302 | Pepper severe mosaic virus uid17809 | NC 008393.1 | IV |
| 1303 | Pepper veinal mottle virus uid33675 | NC 011918.1 | IV |
| 1304 | Pepper yellow dwarf virus New Mexico uid31127 | NC 011188.1 | II |
| 1305 | Pepper yellow mosaic virus uid50567 | NC 014327.1 | IV |
| 1306 | Pepper yellow vein Mali virus uid14348 | NC 005347.1 | II |
| 1307 | Perina nuda virus uid14717 | NC 003113.1 | IV |
| 1308 | Periplaneta fuliginosa densovirus uid14091 | NC 000936.1 | II |
| 1309 | Peru tomato mosaic virus uid15406 | NC 004573.1 | IV |
| 1310 | Peste des petits ruminants virus uid15499 | NC 006383.2 | V |
| 1311 | Pestivirus Giraffe 1 uid14780 | NC 003678.1 | IV |
| 1312 | Petunia vein clearing virus uid14031 | NC 001839.2 | VII |
| 1313 | Phage Gifsy 1 uid32269 | NC 010392.1 | |
| 1314 | Phage Gifsy 2 uid32271 | NC 010393.1 | |
| 1315 | Phage phiJL001 uid16076 | NC 006938.1 | I |
| 1316 | Phaius virus X uid28617 | NC 010295.1 | IV |
| 1317 | Phlebiopsis gigantea mycovirus dsRNA 1 uid46855 | NC 013999.1 | III |
| 1318 | Phlox Virus B uid27905 | NC 009991.1 | IV |
| 1319 | Phlox virus S uid19427 | NC 009383.1 | IV |
| 1320 | Phocoena spinipinnis papillomavirus uid15186 | NC 003348.1 | I |
| 1321 | Phormidium phage Pf WMP3 uid19801 | NC 009551.1 | I |
| 1322 | Phormidium phage Pf WMP4 uid17743 | NC 008367.1 | I |
| 1323 | Phthorimaea operculella granulovirus uid14202 | NC 004062.1 | I |
| 1324 | Physalis mottle virus uid15090 | NC 003634.1 | IV |
| 1325 | Phytophthora endornavirus 1 uid15418 | NC 007069.1 | III |
| 1326 | Pieris rapae granulovirus uid45911 | NC 013797.1 | I |
| 1327 | Pineapple mealybug wilt associated virus 1 uid28147 | NC 010178.1 | IV |
| 1328 | Planococcus citri densovirus uid14223 | NC 004289.1 | II |
| 1329 | Plantago asiatica mosaic virus uid15073 | NC 003849.1 | IV |
| 1330 | Plantago mottle virus uid32683 | NC 011539.1 | IV |
| 1331 | Plautia stali intestine virus uid14799 | NC 003779.1 | IV |
| 1332 | Plum bark necrosis and stem pitting associated virus uid27909 | NC 009992.1 | IV |
| 1333 | Plum pox virus uid15298 | NC 001445.1 | IV |
| 1334 | Plutella xylostella granulovirus uid14104 | NC 002593.1 | I |
| 1335 | Plutella xylostella multiple nucleopolyhedrovirus uid17671 | NC 008349.1 | I |
| 1336 | Pneumonia virus of mice J3666 uid15251 | NC 006579.1 | V |
| 1337 | Poinsettia cryptic virus uid32691 | NC 011543.1 | IV |
| 1338 | Poinsettia mosaic virus uid15366 | NC 002164.1 | IV |
| 1339 | Poliovirus uid15288 | NC 002058.3 | IV |
| 1340 | Polyomavirus HPyV6 uid51559 | NC 014406.1 | I |

| | | | |
|---|---|---|---|
| 1341 | Polyomavirus HPyV7 uid51557 | NC 014407.1 | I |
| 1342 | Poplar mosaic virus uid15056 | NC 005343.1 | IV |
| 1343 | Porcine adenovirus A uid14610 | NC 005869.1 | I |
| 1344 | Porcine adenovirus C uid14521 | NC 002702.1 | I |
| 1345 | Porcine circovirus 1 uid14053 | NC 001792.2 | II |
| 1346 | Porcine circovirus 2 uid15442 | NC 005148.1 | II |
| 1347 | Porcine endogenous retrovirus E uid14126 | NC 003059.1 | VI |
| 1348 | Porcine enteric sapovirus uid14653 | NC 000940.1 | IV |
| 1349 | Porcine enterovirus B uid15396 | NC 004441.1 | IV |
| 1350 | Porcine epidemic diarrhea virus uid14739 | NC 003436.1 | IV |
| 1351 | Porcine hemagglutinating encephalomyelitis virus uid16332 | NC 007732.1 | IV |
| 1352 | Porcine kobuvirus swine S 1 HUN 2007 Hungary uid33533 | NC 011829.1 | IV |
| 1353 | Porcine parvovirus uid14055 | NC 001718.1 | II |
| 1354 | Porcine respiratory and reproductive syndrome virus uid15437 | NC 001961.1 | IV |
| 1355 | Porcine rubulavirus uid20055 | NC 009640.1 | V |
| 1356 | Porcine teschovirus uid15092 | NC 003985.1 | IV |
| 1357 | Possum enterovirus W1 uid18517 | NC 008714.1 | IV |
| 1358 | Possum enterovirus W6 uid18519 | NC 008715.1 | IV |
| 1359 | Potato apical leaf curl disease associated satellite DNA beta uid18323 | NC 008605.1 | NA |
| 1360 | Potato aucuba mosaic virus uid14771 | NC 003632.1 | IV |
| 1361 | Potato latent virus uid32629 | NC 011525.1 | IV |
| 1362 | Potato leafroll virus uid15068 | NC 001747.1 | IV |
| 1363 | Potato virus A uid15376 | NC 004039.1 | IV |
| 1364 | Potato virus M uid15324 | NC 001361.2 | IV |
| 1365 | Potato Virus P uid20657 | NC 009759.1 | IV |
| 1366 | Potato virus S uid15574 | NC 007289.1 | IV |
| 1367 | Potato virus T uid30735 | NC 011062.1 | IV |
| 1368 | Potato virus V uid15379 | NC 004010.1 | IV |
| 1369 | Potato virus X uid15503 | NC 011620.1 | IV |
| 1370 | Potato virus Y uid15290 | NC 001616.1 | IV |
| 1371 | Pothos latent virus uid15185 | NC 000939.1 | IV |
| 1372 | Powassan virus uid15304 | NC 003687.1 | IV |
| 1373 | Prochlorococcus phage P SSM4 uid15136 | NC 006884.2 | I |
| 1374 | Propionibacterium phage B5 uid14163 | NC 003460.1 | II |
| 1375 | Propionibacterium phage PA6 uid19767 | NC 009541.1 | I |
| 1376 | Providence virus uid48417 | NC 014126.1 | IV |
| 1377 | Pseudaletia unipuncta granulovirus uid43731 | NC 013772.1 | I |
| 1378 | Pseudoalteromonas phage PM2 uid14237 | NC 000867.1 | I |
| 1379 | Pseudocowpox virus uid45973 | NC 013804.1 | I |
| 1380 | Pseudomonas phage 119X uid16385 | NC 007807.1 | I |
| 1381 | Pseudomonas phage 14 1 uid33265 | NC 011703.1 | I |
| 1382 | Pseudomonas phage 201phi2 1 uid30097 | NC 010821.1 | I |
| 1383 | Pseudomonas phage 73 uid16384 | NC 007806.1 | I |
| 1384 | Pseudomonas phage B3 uid14542 | NC 006548.1 | I |
| 1385 | Pseudomonas phage D3 uid14500 | NC 002484.1 | I |
| 1386 | Pseudomonas phage D3112 uid14334 | NC 005178.1 | I |
| 1387 | Pseudomonas phage DMS3 uid18521 | NC 008717.1 | I |
| 1388 | Pseudomonas phage EL uid16199 | NC 007623.1 | I |
| 1389 | Pseudomonas phage F10 uid16383 | NC 007805.1 | I |

| | | | |
|---|---|---|---|
| 1390 | Pseudomonas phage F116 uid15127 | NC 006552.1 | I |
| 1391 | Pseudomonas phage F8 uid16388 | NC 007810.1 | I |
| 1392 | Pseudomonas phage gh 1 uid14265 | NC 004665.1 | I |
| 1393 | Pseudomonas phage LBL3 uid31053 | NC 011165.1 | I |
| 1394 | Pseudomonas phage LIT1 uid42949 | NC 013692.1 | I |
| 1395 | Pseudomonas phage LKA1 uid21045 | NC 009936.1 | I |
| 1396 | Pseudomonas phage LKD16 uid21043 | NC 009935.1 | I |
| 1397 | Pseudomonas phage LMA2 uid31055 | NC 011166.1 | I |
| 1398 | Pseudomonas phage LUZ19 uid28741 | NC 010326.1 | I |
| 1399 | Pseudomonas phage LUZ24 uid28739 | NC 010325.1 | I |
| 1400 | Pseudomonas phage LUZ7 uid42951 | NC 013691.1 | I |
| 1401 | Pseudomonas phage M6 uid16387 | NC 007809.1 | I |
| 1402 | Pseudomonas phage MP22 uid20961 | NC 009818.1 | I |
| 1403 | Pseudomonas phage MP29 uid32999 | NC 011613.1 | I |
| 1404 | Pseudomonas phage MP38 uid32995 | NC 011611.1 | I |
| 1405 | Pseudomonas phage PA11 uid16386 | NC 007808.1 | I |
| 1406 | Pseudomonas phage PAJU2 uid32249 | NC 011373.1 | I |
| 1407 | Pseudomonas phage PaP2 uid14377 | NC 005884.1 | I |
| 1408 | Pseudomonas phage PaP3 uid14322 | NC 004466.2 | I |
| 1409 | Pseudomonas phage PB1 uid33499 | NC 011810.1 | I |
| 1410 | Pseudomonas phage Pf1 uid14571 | NC 001331.1 | II |
| 1411 | Pseudomonas phage Pf3 uid14061 | NC 001418.1 | II |
| 1412 | Pseudomonas phage phi 2 uid42717 | NC 013638.1 | I |
| 1413 | Pseudomonas phage phiCTX uid14415 | NC 003278.1 | I |
| 1414 | Pseudomonas phage phikF77 uid36373 | NC 012418.1 | I |
| 1415 | Pseudomonas phage phiKMV uid15226 | NC 005045.1 | I |
| 1416 | Pseudomonas phage phiKZ uid14251 | NC 004629.1 | I |
| 1417 | Pseudomonas phage PP7 uid15076 | NC 001628.1 | IV |
| 1418 | Pseudomonas phage PRR1 uid17481 | NC 008294.1 | IV |
| 1419 | Pseudomonas phage PT2 uid30851 | NC 011107.1 | I |
| 1420 | Pseudomonas phage PT5 uid30847 | NC 011105.1 | I |
| 1421 | Pseudomonas phage SN uid33327 | NC 011756.1 | I |
| 1422 | Pseudomonas phage YuA uid28053 | NC 010116.1 | I |
| 1423 | Psittacid herpesvirus 1 uid14314 | NC 005264.1 | I |
| 1424 | Psittacus erithacus timneh papillomavirus uid14195 | NC 003973.1 | I |
| 1425 | Pumpkin yellow mosaic Malaysia virus uid30159 | NC 010946.1 | II |
| 1426 | Pyrobaculum spherical virus uid14374 | NC 005872.1 | I |
| 1427 | Pyrococcus abyssi virus 1 uid19929 | NC 009597.1 | I |
| 1428 | Quang Binh virus uid37969 | NC 012671.1 | IV |
| 1429 | Rabbit calicivirus Australia 1 MIC 07 uid33267 | NC 011704.1 | IV |
| 1430 | Rabbit fibroma virus uid14590 | NC 001266.1 | I |
| 1431 | Rabbit hemorrhagic disease virus uid15313 | NC 001543.1 | IV |
| 1432 | Rabbit oral papillomavirus uid14057 | NC 002232.1 | I |
| 1433 | Rabbit vesivirus uid18289 | NC 008580.1 | IV |
| 1434 | Rabies virus uid15144 | NC 001542.1 | V |
| 1435 | Rachiplusia ou MNPV uid14229 | NC 004323.1 | I |
| 1436 | Radish leaf curl virus betasatellite uid28281 | NC 010239.1 | NA |
| 1437 | Radish leaf curl virus uid28279 | NC 010238.1 | II |
| 1438 | Ralstonia phage p12J uid14307 | NC 005131.2 | II |
| 1439 | Ralstonia phage phiRSA1 uid19481 | NC 009382.1 | I |

| | | | |
|---|---|---|---|
| 1440 | Ralstonia phage RSB1 uid31163 | NC 011201.1 | I |
| 1441 | Ralstonia phage RSL1 uid30059 | NC 010811.2 | I |
| 1442 | Ralstonia phage RSM1 uid18239 | NC 008574.1 | II |
| 1443 | Ralstonia phage RSM3 uid32325 | NC 011399.1 | II |
| 1444 | Ralstonia phage RSS1 uid18291 | NC 008575.1 | II |
| 1445 | Ranid herpesvirus 1 uid17181 | NC 008211.1 | I |
| 1446 | Ranid herpesvirus 2 uid17183 | NC 008210.1 | I |
| 1447 | Raspberry leaf mottle virus uid18275 | NC 008585.1 | IV |
| 1448 | Rat coronavirus Parker uid39313 | NC 012936.1 | IV |
| 1449 | Rattus norvegicus papillomavirus 1 EES 2009 uid40233 | NC 013196.1 | I |
| 1450 | Rauscher murine leukemia virus uid14907 | NC 001819.1 | VI |
| 1451 | Raven circovirus uid17773 | NC 008375.1 | II |
| 1452 | RD114 retrovirus uid20979 | NC 009889.1 | VI |
| 1453 | Red clover vein mosaic virus uid34841 | NC 012210.1 | IV |
| 1454 | Rehmannia mosaic virus uid18885 | NC 009041.1 | IV |
| 1455 | Respiratory syncytial virus uid15004 | NC 001803.1 | V |
| 1456 | Reston ebolavirus uid15006 | NC 004161.1 | V |
| 1457 | Reticuloendotheliosis virus uid15145 | NC 006934.1 | VI |
| 1458 | Rhesus monkey papillomavirus 1 uid14025 | NC 001678.1 | I |
| 1459 | Rhizobium phage 16 3 uid30845 | NC 011103.1 | I |
| 1460 | Rhododendron virus A uid51905 | NC 014481.1 | III |
| 1461 | Rhodothermus phage RM378 uid14420 | NC 004735.1 | I |
| 1462 | Rhopalosiphum padi virus uid14648 | NC 001874.1 | IV |
| 1463 | Ribgrass mosaic virus uid14980 | NC 002792.1 | IV |
| 1464 | Rice tungro bacilliform virus uid14579 | NC 001914.1 | VII |
| 1465 | Rice tungro spherical virus uid15332 | NC 001632.1 | IV |
| 1466 | Rice yellow mottle virus satellite uid14152 | NC 003380.1 | NA |
| 1467 | Rice yellow mottle virus uid15327 | NC 001575.1 | IV |
| 1468 | Rice yellow stunt virus uid14793 | NC 003746.1 | V |
| 1469 | Rinderpest virus strain Kabete O uid15050 | NC 006296.2 | V |
| 1470 | Rio Bravo virus uid15368 | NC 003675.1 | IV |
| 1471 | Rose spring dwarf associated virus uid30051 | NC 010806.1 | IV |
| 1472 | Roseobacter phage SIO1 uid14308 | NC 002519.1 | I |
| 1473 | Roseophage DSS3P2 uid38081 | NC 012697.1 | I |
| 1474 | Roseophage EE36P1 uid38079 | NC 012696.1 | I |
| 1475 | Ross River virus uid15314 | NC 001544.1 | IV |
| 1476 | Ross s goose hepatitis B virus uid14380 | NC 005888.1 | VII |
| 1477 | Rous sarcoma virus uid14978 | NC 001407.1 | VI |
| 1478 | Rousettus aegyptiacus papillomavirus type 1 uid17549 | NC 008298.1 | I |
| 1479 | Rubella virus uid15315 | NC 001545.2 | IV |
| 1480 | Rubus chlorotic mottle virus uid31125 | NC 011187.1 | IV |
| 1481 | Rudbeckia flower distortion virus uid33679 | NC 011920.1 | VII |
| 1482 | Rupestris stem pitting associated virus uid15249 | NC 001948.1 | IV |
| 1483 | Ryegrass mosaic virus uid15344 | NC 001814.1 | IV |
| 1484 | Ryegrass mottle virus uid15375 | NC 003747.1 | IV |
| 1485 | Sacbrood virus uid14688 | NC 002066.1 | IV |
| 1486 | Saccharomyces 20S RNA narnavirus uid14841 | NC 004051.1 | IV |
| 1487 | Saccharomyces 23S RNA narnavirus uid14840 | NC 004050.1 | IV |
| 1488 | Saccharomyces cerevisiae killer virus M1 uid14678 | NC 001782.1 | III |
| 1489 | Saccharomyces cerevisiae virus L A L1 uid14792 | NC 003745.1 | III |

| | | | |
|---|---|---|---|
| 1490 | Saccharomyces cerevisiae virus L BC  La  uid14643 | NC 001641.1 | III |
| 1491 | Saccharum streak virus uid41611 | NC 013464.1 | II |
| 1492 | Saffold virus uid19577 | NC 009448.2 | IV |
| 1493 | Saguaro cactus virus uid14981 | NC 001780.1 | IV |
| 1494 | Saimiriine herpesvirus 2 uid14417 | NC 001350.1 | I |
| 1495 | Salivirus NG J1 uid39349 | NC 012957.1 | IV |
| 1496 | Salmon pancreas disease virus uid15187 | NC 003930.1 | IV |
| 1497 | Salmonella phage c341 uid39795 | NC 013059.1 | I |
| 1498 | Salmonella phage E1 uid29079 | NC 010495.1 | I |
| 1499 | Salmonella phage epsilon34 uid33779 | NC 011976.1 | I |
| 1500 | Salmonella phage Fels 1 uid29267 | NC 010391.1 | |
| 1501 | Salmonella phage phiSG JL2 uid30063 | NC 010807.1 | I |
| 1502 | Salmonella phage SE1 uid33483 | NC 011802.1 | I |
| 1503 | Salmonella phage SETP3 uid19157 | NC 009232.1 | I |
| 1504 | Salmonella phage ST64B uid14228 | NC 004313.1 | I |
| 1505 | Sapovirus C12 uid15040 | NC 006554.1 | IV |
| 1506 | Sapovirus Hu Dresden pJG Sap01 DE uid15048 | NC 006269.1 | IV |
| 1507 | Sapovirus Mc10 uid14952 | NC 010624.1 | IV |
| 1508 | Satellites of Trichomonas vaginalis T1 virus uid14201 | NC 004048.1 | NA |
| 1509 | Scallion mosaic virus uid15190 | NC 003399.1 | IV |
| 1510 | Scallion virus X uid15099 | NC 003400.1 | IV |
| 1511 | Schizochytrium single stranded RNA virus uid16134 | NC 007522.1 | IV |
| 1512 | Schlumbergera virus X uid33189 | NC 011659.1 | IV |
| 1513 | Sclerophthora macrospora virus B uid14912 | NC 004714.1 | IV |
| 1514 | Sclerotinia sclerotiorum debilitation associated RNA virus uid15717 | NC 007415.1 | IV |
| 1515 | Sclerotinia sclerotiorum hypovirulence associated DNA virus 1 uid39985 | NC 013116.1 | II |
| 1516 | Scotophilus bat coronavirus 512 05 uid20135 | NC 009657.1 | IV |
| 1517 | Scrophularia mottle virus uid32679 | NC 011537.1 | IV |
| 1518 | Sea turtle tornovirus 1 uid34541 | NC 012094.1 | II |
| 1519 | Seal picornavirus type 1 uid20985 | NC 009891.1 | IV |
| 1520 | Semliki forest virus uid15282 | NC 003215.1 | IV |
| 1521 | Sendai virus uid15023 | NC 001552.1 | V |
| 1522 | Seneca valley virus uid32193 | NC 011349.1 | IV |
| 1523 | Senecio yellow mosaic virus uid15233 | NC 006995.1 | II |
| 1524 | Sepik virus uid18513 | NC 008719.1 | IV |
| 1525 | Sesbania mosaic virus uid15372 | NC 002568.2 | IV |
| 1526 | Shallot virus X uid14805 | NC 003795.1 | IV |
| 1527 | Shallot yellow stripe virus uid15745 | NC 007433.1 | IV |
| 1528 | Sheeppox virus uid14196 | NC 004002.1 | I |
| 1529 | Sheldgoose hepatitis B virus uid14618 | NC 005890.1 | VII |
| 1530 | Shigella phage phiSboM AG3 uid42937 | NC 013693.1 | I |
| 1531 | Shrimp white spot syndrome virus uid14616 | NC 003225.1 | I |
| 1532 | Sida golden mosaic Florida virus uid14254 | NC 004636.1 | II |
| 1533 | Sida leaf curl virus associated DNA 1 uid16227 | NC 007640.1 | NA |
| 1534 | Sida leaf curl virus associated DNA beta uid16226 | NC 007639.1 | NA |
| 1535 | Sida leaf curl virus satellite DNA beta uid19823 | NC 009557.1 | NA |
| 1536 | Sida leaf curl virus uid16225 | NC 007638.1 | II |
| 1537 | Sida mottle virus uid14255 | NC 004637.1 | II |
| 1538 | Sida yellow mosaic virus China associated DNA beta uid15514 | NC 006267.1 | NA |

| | | | |
|---|---|---|---|
| 1539 | Sida yellow mosaic virus uid15496 | NC 004639.1 | II |
| 1540 | Sida yellow vein disease associated DNA 1 uid48075 | NC 014065.1 | NA |
| 1541 | Sida yellow vein Madurai virus uid19405 | NC 009354.1 | II |
| 1542 | Sida yellow vein Vietnam virus associated DNA 1 uid19815 | NC 009563.1 | NA |
| 1543 | Sida yellow vein Vietnam virus satellite DNA beta uid19825 | NC 009558.1 | NA |
| 1544 | Sida yellow vein Vietnam virus uid19783 | NC 009547.1 | II |
| 1545 | Sida yellow vein virus satellite DNA beta uid15562 | NC 007213.1 | NA |
| 1546 | Siegesbeckia yellow vein Guangxi virus uid17595 | NC 008317.1 | II |
| 1547 | Siegesbeckia yellow vein virus associated DNA beta uid17269 | NC 008237.1 | NA |
| 1548 | Siegesbeckia yellow vein virus uid17267 | NC 008236.1 | II |
| 1549 | Simian adenovirus 1 uid14626 | NC 006879.1 | I |
| 1550 | Simian adenovirus 3 uid14491 | NC 006144.1 | I |
| 1551 | Simian agent 12 uid16189 | NC 007611.1 | I |
| 1552 | Simian enterovirus A uid15371 | NC 003988.1 | IV |
| 1553 | Simian enterovirus SV19 uid29251 | NC 010412.1 | IV |
| 1554 | Simian enterovirus SV43 uid29253 | NC 010413.1 | IV |
| 1555 | Simian enterovirus SV6 uid29255 | NC 010415.1 | IV |
| 1556 | Simian foamy virus uid14699 | NC 001364.1 | VI |
| 1557 | Simian hemorrhagic fever virus uid14727 | NC 003092.1 | IV |
| 1558 | Simian Human immunodeficiency virus uid15034 | NC 001870.1 | VI |
| 1559 | Simian immunodeficiency virus SIV mnd 2 uid14872 | NC 004455.1 | VI |
| 1560 | Simian immunodeficiency virus uid15501 | NC 001549.1 | VI |
| 1561 | Simian picornavirus 17 uid29241 | NC 010411.1 | IV |
| 1562 | Simian picornavirus N125 uid29243 | NC 010384.1 | IV |
| 1563 | Simian picornavirus N203 uid42941 | NC 013695.1 | IV |
| 1564 | Simian retrovirus 4 uid51791 | NC 014474.1 | VI |
| 1565 | Simian T cell lymphotropic virus 6 uid32697 | NC 011546.1 | VI |
| 1566 | Simian T lymphotropic virus 1 uid15439 | NC 000858.1 | VI |
| 1567 | Simian T lymphotropic virus 2 uid15221 | NC 001815.1 | VI |
| 1568 | Simian T lymphotropic virus 3 uid14732 | NC 003323.1 | VI |
| 1569 | Simian virus 12 uid34707 | NC 012122.1 | I |
| 1570 | Simian virus 40 uid14024 | NC 001669.1 | I |
| 1571 | Simian virus 41 uid15220 | NC 006428.1 | V |
| 1572 | Sindbis virus uid15316 | NC 001547.1 | IV |
| 1573 | Singapore grouper iridovirus uid14544 | NC 006549.1 | I |
| 1574 | Siniperca chuatsi rhabdovirus uid18009 | NC 008514.1 | V |
| 1575 | Sinorhizobium phage PBC5 uid14146 | NC 003324.1 | I |
| 1576 | Sleeping disease virus uid15395 | NC 003433.1 | IV |
| 1577 | Slow bee paralysis virus uid48587 | NC 014137.1 | IV |
| 1578 | Small anellovirus 1 uid15252 | NC 007013.1 | II |
| 1579 | Small anellovirus 2 uid15253 | NC 007014.1 | II |
| 1580 | Snake parvovirus 1 uid14477 | NC 006148.1 | II |
| 1581 | Snakehead retrovirus uid14701 | NC 001724.1 | VI |
| 1582 | Snakehead rhabdovirus uid14689 | NC 000903.1 | V |
| 1583 | Snow goose hepatitis B virus uid14403 | NC 005950.1 | VII |
| 1584 | Sodalis phage phiSG1 uid16583 | NC 007902.1 | |
| 1585 | Sodalis phage SO 1 uid42597 | NC 013600.1 | I |
| 1586 | Soft shelled turtle iridovirus uid37823 | NC 012637.1 | I |
| 1587 | Solanum nodiflorum mottle virus satellite RNA uid14184 | NC 003850.1 | NA |
| 1588 | Solenopsis invicta virus 1 uid15042 | NC 006559.1 | IV |

| | | | |
|---|---|---|---|
| 1589 | Solenopsis invicta virus 2 uid19773 | NC 009544.1 | IV |
| 1590 | Solenopsis invicta virus 3 uid36613 | NC 012531.1 | IV |
| 1591 | Sonchus yellow net virus uid14642 | NC 001615.2 | V |
| 1592 | Sorghum mosaic virus uid15098 | NC 004035.1 | IV |
| 1593 | Southern bean mosaic virus uid15356 | NC 004060.1 | IV |
| 1594 | Southern cowpea mosaic virus uid15331 | NC 001625.1 | IV |
| 1595 | Southern tomato virus uid32821 | NC 011591.1 | III |
| 1596 | Soybean chlorotic mottle virus uid14594 | NC 001739.2 | VII |
| 1597 | Soybean crinkle leaf virus uid14149 | NC 003357.1 | II |
| 1598 | Soybean dwarf virus uid14715 | NC 003056.1 | IV |
| 1599 | Soybean mild mottle virus uid48593 | NC 014140.1 | II |
| 1600 | Soybean mosaic virus uid15377 | NC 002634.1 | IV |
| 1601 | Soybean yellow mottle mosaic virus uid33135 | NC 011643.1 | IV |
| 1602 | Sphaeropsis sapinea RNA virus 1 uid14722 | NC 001963.1 | III |
| 1603 | Sphaeropsis sapinea RNA virus 2 uid14687 | NC 001964.1 | III |
| 1604 | Spilanthes yellow vein virus uid19779 | NC 009545.1 | II |
| 1605 | Spinach curly top virus uid14373 | NC 005860.1 | II |
| 1606 | Spiroplasma kunkelii virus SkV1 CR2 3x uid27891 | NC 009987.1 | II |
| 1607 | Spiroplasma phage 1 C74 uid14178 | NC 003793.1 | II |
| 1608 | Spiroplasma phage 1 R8A2B uid14580 | NC 001365.1 | II |
| 1609 | Spiroplasma phage 4 uid14161 | NC 003438.1 | II |
| 1610 | Spiroplasma phage SVTS2 uid14032 | NC 001270.2 | II |
| 1611 | Spissistilus festinus virus 1 uid51181 | NC 014359.1 | III |
| 1612 | Spleen focus forming virus uid14641 | NC 001500.1 | VI |
| 1613 | Spodoptera exigua MNPV uid14134 | NC 002169.1 | I |
| 1614 | Spodoptera frugiperda ascovirus 1a uid17721 | NC 008361.1 | I |
| 1615 | Spodoptera frugiperda MNPV uid18827 | NC 009011.2 | I |
| 1616 | Spodoptera litura granulovirus uid19695 | NC 009503.1 | I |
| 1617 | Spodoptera litura NPV uid14138 | NC 003102.1 | I |
| 1618 | Spodoptera litura nucleopolyhedrovirus II uid33005 | NC 011616.1 | I |
| 1619 | Spring viraemia of carp virus uid14726 | NC 002803.1 | V |
| 1620 | Sputnik virophage uid30929 | NC 011132.1 | |
| 1621 | Squash leaf curl Yunnan virus uid15194 | NC 004651.1 | II |
| 1622 | Squash vein yellowing virus uid29107 | NC 010521.1 | IV |
| 1623 | Squirrel monkey polyomavirus uid27775 | NC 009951.1 | I |
| 1624 | Squirrel monkey retrovirus uid14914 | NC 001514.1 | VI |
| 1625 | Stachytarpheta leaf curl virus uid14412 | NC 004091.1 | II |
| 1626 | Staphylococcus aureus phage P68 uid14269 | NC 004679.1 | I |
| 1627 | Staphylococcus phage 11 uid14246 | NC 004615.1 | I |
| 1628 | Staphylococcus phage 187 uid15264 | NC 007047.1 | I |
| 1629 | Staphylococcus phage 2638A uid15267 | NC 007051.1 | I |
| 1630 | Staphylococcus phage 29 uid15277 | NC 007061.1 | I |
| 1631 | Staphylococcus phage 37 uid15271 | NC 007055.1 | I |
| 1632 | Staphylococcus phage 3A uid15269 | NC 007053.1 | I |
| 1633 | Staphylococcus phage 42E uid15268 | NC 007052.1 | I |
| 1634 | Staphylococcus phage 44AHJD uid14268 | NC 004678.1 | I |
| 1635 | Staphylococcus phage 47 uid15270 | NC 007054.1 | I |
| 1636 | Staphylococcus phage 52A uid15278 | NC 007062.1 | I |
| 1637 | Staphylococcus phage 53 uid15266 | NC 007049.1 | I |
| 1638 | Staphylococcus phage 55 uid15276 | NC 007060.1 | I |

| | | | |
|---|---|---|---|
| 1639 | Staphylococcus phage 66 uid15263 | NC 007046.1 | I |
| 1640 | Staphylococcus phage 69 uid15265 | NC 007048.1 | I |
| 1641 | Staphylococcus phage 71 uid15275 | NC 007059.1 | I |
| 1642 | Staphylococcus phage 77 uid14352 | NC 005356.1 | I |
| 1643 | Staphylococcus phage 80alpha uid19749 | NC 009526.1 | I |
| 1644 | Staphylococcus phage 85 uid15260 | NC 007050.1 | I |
| 1645 | Staphylococcus phage 88 uid15279 | NC 007063.1 | I |
| 1646 | Staphylococcus phage 92 uid15280 | NC 007064.1 | I |
| 1647 | Staphylococcus phage 96 uid15273 | NC 007057.1 | I |
| 1648 | Staphylococcus phage CNPH82 uid18523 | NC 008722.1 | I |
| 1649 | Staphylococcus phage EW uid15272 | NC 007056.1 | I |
| 1650 | Staphylococcus phage G1 uid15261 | NC 007066.1 | I |
| 1651 | Staphylococcus phage K uid14479 | NC 005880.1 | I |
| 1652 | Staphylococcus phage P954 uid40231 | NC 013195.1 | I |
| 1653 | Staphylococcus phage PH15 uid18525 | NC 008723.1 | I |
| 1654 | Staphylococcus phage phi 12 uid14247 | NC 004616.1 | I |
| 1655 | Staphylococcus phage phi13 uid14248 | NC 004617.1 | I |
| 1656 | Staphylococcus phage phi2958PVL uid32173 | NC 011344.1 | |
| 1657 | Staphylococcus phage phiETA uid14141 | NC 003288.1 | I |
| 1658 | Staphylococcus phage phiETA2 uid18669 | NC 008798.1 | I |
| 1659 | Staphylococcus phage phiETA3 uid18671 | NC 008799.1 | I |
| 1660 | Staphylococcus phage phiMR11 uid28065 | NC 010147.1 | I |
| 1661 | Staphylococcus phage phiMR25 uid30061 | NC 010808.1 | I |
| 1662 | Staphylococcus phage phiN315 uid14527 | NC 004740.1 | I |
| 1663 | Staphylococcus phage phiNM uid18293 | NC 008583.1 | I |
| 1664 | Staphylococcus phage phiNM3 uid18329 | NC 008617.1 | I |
| 1665 | Staphylococcus phage phiPVL CN125 uid38431 | NC 012784.1 | I |
| 1666 | Staphylococcus phage phiPVL108 uid18463 | NC 008689.1 | I |
| 1667 | Staphylococcus phage phiSauS IPLA35 uid32997 | NC 011612.1 | I |
| 1668 | Staphylococcus phage phiSauS IPLA88 uid33001 | NC 011614.1 | I |
| 1669 | Staphylococcus phage phiSLT uid14137 | NC 002661.2 | I |
| 1670 | Staphylococcus phage PT1028 uid15262 | NC 007045.1 | I |
| 1671 | Staphylococcus phage PVL uid14392 | NC 002321.1 | I |
| 1672 | Staphylococcus phage ROSA uid15274 | NC 007058.1 | I |
| 1673 | Staphylococcus phage SAP 2 uid20925 | NC 009875.1 | I |
| 1674 | Staphylococcus phage SAP 26 uid51671 | NC 014460.1 | I |
| 1675 | Staphylococcus phage tp310 1 uid20659 | NC 009761.2 | |
| 1676 | Staphylococcus phage tp310 2 uid20661 | NC 009762.2 | |
| 1677 | Staphylococcus phage tp310 3 uid20663 | NC 009763.2 | |
| 1678 | Staphylococcus phage Twort uid15246 | NC 007021.1 | I |
| 1679 | Staphylococcus phage X2 uid15281 | NC 007065.1 | I |
| 1680 | Staphylococcus prophage phiPV83 uid14135 | NC 002486.1 | I |
| 1681 | Starling circovirus uid16796 | NC 008033.1 | II |
| 1682 | Steller sea lion vesivirus uid30663 | NC 011050.1 | IV |
| 1683 | Stenotrophomonas phage phiSMA9 uid15493 | NC 007189.1 | |
| 1684 | Stenotrophomonas phage S1 uid32787 | NC 011589.1 | I |
| 1685 | Strawberry chlorotic fleck associated virus uid17741 | NC 008366.1 | IV |
| 1686 | Strawberry latent ringspot virus satellite RNA uid15155 | NC 003848.1 | NA |
| 1687 | Strawberry mild yellow edge virus uid14999 | NC 003794.1 | IV |
| 1688 | Strawberry vein banding virus uid15207 | NC 001725.1 | VII |

| | | | |
|---|---|---|---|
| 1689 | Streptocarpus flower break virus uid17803 | NC 008365.1 | IV |
| 1690 | Streptococcus phage 2972 uid15254 | NC 007019.1 | I |
| 1691 | Streptococcus phage 5093 uid38299 | NC 012753.1 | I |
| 1692 | Streptococcus phage 7201 uid14051 | NC 002185.1 | I |
| 1693 | Streptococcus phage 858 uid28829 | NC 010353.1 | I |
| 1694 | Streptococcus phage Abc2 uid42791 | NC 013645.1 | I |
| 1695 | Streptococcus phage ALQ13 2 uid42593 | NC 013598.1 | I |
| 1696 | Streptococcus phage C1 uid14288 | NC 004814.1 | I |
| 1697 | Streptococcus phage Cp 1 uid14584 | NC 001825.1 | I |
| 1698 | Streptococcus phage DT1 uid15124 | NC 002072.2 | I |
| 1699 | Streptococcus phage EJ 1 uid14604 | NC 005294.1 | I |
| 1700 | Streptococcus phage M102 uid38845 | NC 012884.1 | I |
| 1701 | Streptococcus phage MM1 uid14601 | NC 003050.2 | I |
| 1702 | Streptococcus phage O1205 uid14226 | NC 004303.1 | I |
| 1703 | Streptococcus phage P9 uid20785 | NC 009819.1 | I |
| 1704 | Streptococcus phage PH10 uid38365 | NC 012756.1 | I |
| 1705 | Streptococcus phage PH15 uid30161 | NC 010945.1 | I |
| 1706 | Streptococcus phage phi3396 uid18859 | NC 009018.1 | I |
| 1707 | Streptococcus phage Sfi11 uid14054 | NC 002214.1 | I |
| 1708 | Streptococcus phage Sfi19 uid14045 | NC 000871.1 | I |
| 1709 | Streptococcus phage Sfi21 uid14133 | NC 000872.1 | I |
| 1710 | Streptococcus phage SM1 uid14295 | NC 004996.1 | I |
| 1711 | Streptococcus phage SMP uid18529 | NC 008721.1 | I |
| 1712 | Streptococcus pyogenes phage 315 1 uid14533 | NC 004584.1 | |
| 1713 | Streptococcus pyogenes phage 315 2 uid14528 | NC 004585.1 | |
| 1714 | Streptococcus pyogenes phage 315 3 uid14529 | NC 004586.1 | |
| 1715 | Streptococcus pyogenes phage 315 4 uid14530 | NC 004587.1 | |
| 1716 | Streptococcus pyogenes phage 315 5 uid14531 | NC 004588.1 | |
| 1717 | Streptococcus pyogenes phage 315 6 uid14532 | NC 004589.1 | |
| 1718 | Streptomyces phage mu1 6 uid16706 | NC 007967.1 | I |
| 1719 | Streptomyces phage phiBT1 uid14276 | NC 004664.2 | I |
| 1720 | Streptomyces phage phiC31 uid14606 | NC 001978.2 | I |
| 1721 | Streptomyces phage phiSASD1 uid49613 | NC 014229.1 | I |
| 1722 | Streptomyces phage VWB uid14485 | NC 005345.2 | I |
| 1723 | Stx1 converting phage uid14293 | NC 004913.2 | I |
| 1724 | Stx2 converting phage 1717 uid32213 | NC 011357.1 | I |
| 1725 | Stx2 converting phage 86 uid17979 | NC 008464.1 | I |
| 1726 | Stx2 converting phage I uid14167 | NC 003525.1 | I |
| 1727 | Stx2 converting phage II uid14310 | NC 004914.2 | I |
| 1728 | Subterranean clover mottle virus satellite RNA uid14503 | NC 003851.1 | NA |
| 1729 | Subterranean clover mottle virus uid15403 | NC 004346.1 | IV |
| 1730 | Sudan ebolavirus uid15012 | NC 006432.1 | V |
| 1731 | Sugarcane bacilliform IM virus uid14123 | NC 003031.1 | VII |
| 1732 | Sugarcane bacilliform Mor virus uid16750 | NC 008017.1 | VII |
| 1733 | Sugarcane bacilliform virus uid41599 | NC 013455.1 | VII |
| 1734 | Sugarcane mosaic virus uid14994 | NC 003398.1 | IV |
| 1735 | Sugarcane streak Egypt virus uid14365 | NC 001868.1 | II |
| 1736 | Sugarcane streak mosaic virus uid47861 | NC 014037.1 | IV |
| 1737 | Sugarcane streak Reunion virus uid14303 | NC 004755.1 | II |
| 1738 | Sugarcane streak virus uid14177 | NC 003744.1 | II |

| | | | |
|---|---|---|---|
| 1739 | Sugarcane striate mosaic associated virus uid14819 | NC 003870.1 | IV |
| 1740 | Sugarcane yellow leaf virus uid15363 | NC 000874.1 | IV |
| 1741 | Suid herpesvirus 1 uid14424 | NC 006151.1 | I |
| 1742 | Sulfolobus islandicus filamentous virus uid14132 | NC 003214.2 | I |
| 1743 | Sulfolobus islandicus rod shaped virus 1 uid14514 | NC 004087.1 | I |
| 1744 | Sulfolobus islandicus rod shaped virus 2 uid15191 | NC 004086.1 | I |
| 1745 | Sulfolobus spindle shaped virus 4 uid27893 | NC 009986.1 | I |
| 1746 | Sulfolobus spindle shaped virus 5 uid31219 | NC 011217.1 | I |
| 1747 | Sulfolobus spindle shaped virus 6 uid42355 | NC 013587.1 | I |
| 1748 | Sulfolobus spindle shaped virus 7 uid42357 | NC 013588.1 | I |
| 1749 | Sulfolobus turreted icosahedral virus 2 uid48299 | NC 014099.1 | I |
| 1750 | Sulfolobus turreted icosahedral virus uid14401 | NC 005892.1 | I |
| 1751 | Sulfolobus virus 1 uid14014 | NC 001338.1 | I |
| 1752 | Sulfolobus virus 2 uid14317 | NC 005265.1 | I |
| 1753 | Sulfolobus virus Kamchatka 1 uid14355 | NC 005361.1 | I |
| 1754 | Sulfolobus virus Ragged Hills uid14354 | NC 005360.1 | I |
| 1755 | Sulfolobus virus STSV1 uid14561 | NC 006268.1 | I |
| 1756 | Sunflower chlorotic mottle virus uid47931 | NC 014038.1 | IV |
| 1757 | Sunn hemp leaf distortion virus uid39609 | NC 013019.1 | II |
| 1758 | Sus scrofa papillomavirus type 1 uid32003 | NC 011280.1 | I |
| 1759 | Sweet potato chlorotic fleck virus uid15038 | NC 006550.1 | IV |
| 1760 | Sweet potato feathery mottle virus uid15347 | NC 001841.1 | IV |
| 1761 | Sweet potato leaf curl Bengal virus uid42745 | NC 013640.2 | II |
| 1762 | Sweet potato leaf curl Canary virus uid41623 | NC 013465.1 | II |
| 1763 | Sweet potato leaf curl Georgia virus uid14257 | NC 004640.1 | II |
| 1764 | Sweet potato leaf curl Lanzarote virus uid41625 | NC 013467.1 | II |
| 1765 | Sweet potato leaf curl Spain virus uid30673 | NC 011052.2 | II |
| 1766 | Sweet potato leaf curl virus uid15461 | NC 004650.1 | II |
| 1767 | Sweet potato mild mottle virus uid15340 | NC 003797.1 | IV |
| 1768 | Sweetpotato badnavirus B uid38241 | NC 012728.1 | VII |
| 1769 | Swinepox virus uid14155 | NC 003389.1 | I |
| 1770 | Synechococcus phage P60 uid14628 | NC 003390.1 | I |
| 1771 | Synechococcus phage S PM2 uid15223 | NC 006820.1 | I |
| 1772 | Synechococcus phage S RSM4 uid39923 | NC 013085.1 | I |
| 1773 | Synechococcus phage Syn5 uid19763 | NC 009531.1 | I |
| 1774 | Synechococcus phage syn9 uid17541 | NC 008296.2 | I |
| 1775 | Tamana bat virus uid15398 | NC 003996.1 | IV |
| 1776 | Tanapox virus uid20981 | NC 009888.1 | I |
| 1777 | Taro bacilliform virus uid14233 | NC 004450.1 | VII |
| 1778 | Taro vein chlorosis virus uid15163 | NC 006942.1 | V |
| 1779 | Taterapox virus uid17483 | NC 008291.1 | I |
| 1780 | Taura syndrome virus uid14713 | NC 003005.1 | IV |
| 1781 | Telosma mosaic virus uid20621 | NC 009742.1 | IV |
| 1782 | Temperate phage phiNIH1 1 uid14145 | NC 003157.4 | I |
| 1783 | Thalassomonas phage BA3 uid27903 | NC 009990.1 | I |
| 1784 | Theilovirus uid15292 | NC 001366.1 | IV |
| 1785 | Thermoproteus tenax spherical virus 1 uid14540 | NC 006556.1 | I |
| 1786 | Thermus phage IN93 uid14235 | NC 004462.2 | I |
| 1787 | Thermus phage P23 45 uid20765 | NC 009803.1 | I |
| 1788 | Thermus phage P23 77 uid40235 | NC 013197.1 | I |

| | | | |
|---|---|---|---|
| 1789 | Thermus phage P74 26 uid20767 | NC 009804.1 | I |
| 1790 | Thermus phage phiYS40 uid18277 | NC 008584.1 | I |
| 1791 | Thielaviopsis basicola mitovirus uid37715 | NC 012585.1 | IV |
| 1792 | Thrush coronavirus HKU12 600 uid32701 | NC 011549.1 | IV |
| 1793 | Thunberg fritillary virus uid15483 | NC 007180.1 | IV |
| 1794 | Tick borne encephalitis virus uid15335 | NC 001672.1 | IV |
| 1795 | Tioman virus uid14846 | NC 004074.1 | V |
| 1796 | Tobacco bushy top virus satellite like RNA uid14511 | NC 006458.1 | NA |
| 1797 | Tobacco bushy top virus uid14868 | NC 004366.1 | IV |
| 1798 | Tobacco curly shoot virus associated DNA 1 uid15480 | NC 005057.1 | NA |
| 1799 | Tobacco curly shoot virus satellite DNA beta uid14446 | NC 004546.1 | NA |
| 1800 | Tobacco curly shoot virus uid15257 | NC 003722.1 | II |
| 1801 | Tobacco etch virus uid15325 | NC 001555.1 | IV |
| 1802 | Tobacco leaf curl disease associated sequence uid14442 | NC 005049.1 | NA |
| 1803 | Tobacco leaf curl Japan virus uid14261 | NC 004654.1 | II |
| 1804 | Tobacco leaf curl Kochi virus uid14400 | NC 004641.1 | II |
| 1805 | Tobacco leaf curl Thailand virus uid19799 | NC 009553.1 | II |
| 1806 | Tobacco leaf curl virus associated DNA beta uid45925 | NC 013800.1 | NA |
| 1807 | Tobacco leaf curl virus uid51907 | NC 014482.1 | II |
| 1808 | Tobacco leaf curl Yunnan virus associated DNA 1 uid15482 | NC 005060.1 | NA |
| 1809 | Tobacco leaf curl Yunnan virus satellite DNA beta uid14539 | NC 005030.1 | NA |
| 1810 | Tobacco leaf curl Yunnan virus uid15258 | NC 004356.1 | II |
| 1811 | Tobacco leaf curl Zimbabwe virus uid14119 | NC 002817.1 | II |
| 1812 | Tobacco mild green mosaic virus uid14671 | NC 001556.1 | IV |
| 1813 | Tobacco mosaic virus uid15071 | NC 001367.1 | IV |
| 1814 | Tobacco necrosis satellite virus uid14672 | NC 001557.1 | NA |
| 1815 | Tobacco necrosis virus A uid15146 | NC 001777.1 | IV |
| 1816 | Tobacco necrosis virus D uid14747 | NC 003487.1 | IV |
| 1817 | Tobacco ringspot virus satellite RNA uid14189 | NC 003889.1 | NA |
| 1818 | Tobacco vein banding mosaic virus uid27895 | NC 009994.1 | IV |
| 1819 | Tobacco vein clearing virus uid14150 | NC 003378.1 | VII |
| 1820 | Tobacco vein distorting virus uid29875 | NC 010732.1 | IV |
| 1821 | Tobacco vein mottling virus uid15348 | NC 001768.1 | IV |
| 1822 | Tobacco yellow dwarf virus uid14181 | NC 003822.1 | II |
| 1823 | Tomato begomovirus satellite DNA beta uid14449 | NC 004904.1 | NA |
| 1824 | Tomato black ring virus satellite RNA uid15016 | NC 003890.1 | NA |
| 1825 | Tomato bushy stunt virus satellite RNA uid14430 | NC 003826.1 | NA |
| 1826 | Tomato bushy stunt virus uid15147 | NC 001554.1 | IV |
| 1827 | Tomato chino La Paz virus uid14368 | NC 005843.1 | II |
| 1828 | Tomato curly stunt virus uid14267 | NC 004675.1 | II |
| 1829 | Tomato leaf curl Arusha virus uid18861 | NC 009030.1 | II |
| 1830 | Tomato leaf curl Bangalore virus Ban5 satellite DNA beta uid28067 | NC 010148.1 | NA |
| 1831 | Tomato leaf curl Bangalore virus uid14190 | NC 003891.1 | II |
| 1832 | Tomato leaf curl Bangladesh virus uid14245 | NC 004614.1 | II |
| 1833 | Tomato leaf curl Cameroon virus uid42743 | NC 013639.1 | II |
| 1834 | Tomato leaf curl Cebu virus uid28987 | NC 010439.1 | II |
| 1835 | Tomato leaf curl China virus satellite DNA beta uid14375 | NC 006289.1 | NA |
| 1836 | Tomato leaf curl China virus uid14342 | NC 005320.1 | II |
| 1837 | Tomato leaf curl Cotabato virus uid28989 | NC 010441.2 | II |

| | | | |
|---|---|---|---|
| 1838 | Tomato leaf curl Ghana virus uid28699 | NC 010313.1 | II |
| 1839 | Tomato leaf curl Guangdong virus uid17805 | NC 008373.1 | II |
| 1840 | Tomato leaf curl Guangxi virus uid17607 | NC 008329.1 | II |
| 1841 | Tomato leaf curl Hainan virus uid39931 | NC 013102.1 | II |
| 1842 | Tomato leaf curl Hsinchu virus uid18627 | NC 008727.1 | II |
| 1843 | Tomato leaf curl Iran virus uid14474 | NC 005842.1 | II |
| 1844 | Tomato leaf curl Java virus Ageratum  satellite DNA uid14452 | NC 005497.1 | NA |
| 1845 | Tomato leaf curl Java virus uid14296 | NC 005031.1 | II |
| 1846 | Tomato leaf curl Joydebpur virus satellite DNA beta uid28273 | NC 010236.1 | NA |
| 1847 | Tomato leaf curl Joydebpur virus uid16324 | NC 007723.1 | II |
| 1848 | Tomato leaf curl Karnataka virus associated DNA beta uid17999 | NC 008523.1 | NA |
| 1849 | Tomato leaf curl Karnataka virus uid14192 | NC 003897.1 | II |
| 1850 | Tomato leaf curl Kerala virus uid30935 | NC 011135.1 | II |
| 1851 | Tomato leaf curl Kumasi virus uid30837 | NC 011096.1 | II |
| 1852 | Tomato leaf curl Laos virus uid14244 | NC 004613.1 | II |
| 1853 | Tomato leaf curl Malaysia virus uid14260 | NC 004648.1 | II |
| 1854 | Tomato leaf curl Mali virus uid14349 | NC 005348.1 | II |
| 1855 | Tomato leaf curl Mayotte virus uid15212 | NC 006876.1 | II |
| 1856 | Tomato leaf curl Mindanao virus uid29011 | NC 010440.1 | II |
| 1857 | Tomato leaf curl New Delhi virus associated DNA beta uid14451 | NC 005359.1 | NA |
| 1858 | Tomato leaf curl Nigeria virus Nigeria 2006  uid34815 | NC 012206.1 | II |
| 1859 | Tomato leaf curl Pakistan virus associated DNA 1 uid38463 | NC 012789.1 | NA |
| 1860 | Tomato leaf curl Pakistan virus uid17539 | NC 008299.1 | II |
| 1861 | Tomato leaf curl Patna virus uid36527 | NC 012492.1 | II |
| 1862 | Tomato leaf curl Philippine virus satellite DNA beta uid19865 | NC 009570.1 | NA |
| 1863 | Tomato leaf curl Philippines virus uid14297 | NC 005032.1 | II |
| 1864 | Tomato leaf curl Pune virus uid18015 | NC 008517.1 | II |
| 1865 | Tomato leaf curl Seychelles virus uid18869 | NC 009031.1 | II |
| 1866 | Tomato leaf curl Sri Lanka virus uid14259 | NC 004647.1 | II |
| 1867 | Tomato leaf curl Sudan virus uid14372 | NC 005855.1 | II |
| 1868 | Tomato leaf curl Sulawesi virus uid41173 | NC 013413.1 | II |
| 1869 | Tomato leaf curl Taiwan virus uid14193 | NC 003898.1 | II |
| 1870 | Tomato leaf curl Togo virus Togo 2006  uid34813 | NC 012205.1 | II |
| 1871 | Tomato leaf curl Vietnam virus uid14214 | NC 004153.1 | II |
| 1872 | Tomato leaf curl virus associated DNA beta uid14622 | NC 004715.1 | NA |
| 1873 | Tomato leaf curl virus Pune associated DNA beta uid18001 | NC 008524.1 | NA |
| 1874 | Tomato leaf curl virus satellite DNA uid14428 | NC 002743.1 | NA |
| 1875 | Tomato leaf curl virus uid14191 | NC 003896.1 | II |
| 1876 | Tomato mosaic virus uid14926 | NC 002692.1 | IV |
| 1877 | Tomato pseudo curly top virus uid14582 | NC 003825.1 | II |
| 1878 | Tomato severe leaf curl virus uid14482 | NC 004642.1 | II |
| 1879 | Tomato yellow dwarf disease associated satellite DNA beta Kochi uid20983 | NC 009893.1 | NA |
| 1880 | Tomato yellow leaf curl China virus associated DNA 1 uid15481 | NC 005058.1 | NA |
| 1881 | Tomato yellow leaf curl China virus satellite DNA beta uid15446 | NC 004544.1 | NA |
| 1882 | Tomato yellow leaf curl China virus uid15318 | NC 004044.1 | II |
| 1883 | Tomato yellow leaf curl Guangdong virus uid17801 | NC 008374.1 | II |
| 1884 | Tomato yellow leaf curl Indonesia virus Lembang  uid17387 | NC 008267.1 | II |
| 1885 | Tomato yellow leaf curl Malaga virus uid14239 | NC 004569.1 | II |
| 1886 | Tomato yellow leaf curl Mali virus associated DNA beta uid15995 | NC 007485.2 | NA |

| | | | |
|---|---|---|---|
| 1887 | Tomato yellow leaf curl Sardinia virus uid14484 | NC 003828.1 | II |
| 1888 | Tomato yellow leaf curl Thailand betasatellite uid42747 | NC 013641.1 | NA |
| 1889 | Tomato yellow leaf curl Thailand virus associated DNA 1 uid14300 | NC 005059.1 | NA |
| 1890 | Tomato yellow leaf curl Thailand virus satellite DNA beta uid14450 | NC 004903.1 | NA |
| 1891 | Tomato yellow leaf curl Vietnam virus satellite DNA beta uid19829 | NC 009560.1 | NA |
| 1892 | Tomato yellow leaf curl Vietnam virus uid19785 | NC 009548.1 | II |
| 1893 | Tomato yellow leaf curl virus associated DNA beta uid28045 | NC 010126.1 | NA |
| 1894 | Tomato yellow leaf curl virus uid15182 | NC 004005.1 | II |
| 1895 | Torque teno canis virus uid48141 | NC 014071.1 | II |
| 1896 | Torque teno douroucouli virus uid48173 | NC 014087.1 | II |
| 1897 | Torque teno felis virus uid48143 | NC 014072.1 | II |
| 1898 | Torque teno midi virus 1 uid19131 | NC 009225.1 | II |
| 1899 | Torque teno midi virus 2 uid48185 | NC 014093.1 | II |
| 1900 | Torque teno mini virus 1 uid48193 | NC 014097.1 | II |
| 1901 | Torque teno mini virus 2 uid48171 | NC 014086.1 | II |
| 1902 | Torque teno mini virus 3 uid48175 | NC 014088.1 | II |
| 1903 | Torque teno mini virus 4 uid48179 | NC 014090.1 | II |
| 1904 | Torque teno mini virus 5 uid48177 | NC 014089.1 | II |
| 1905 | Torque teno mini virus 6 uid48189 | NC 014095.1 | II |
| 1906 | Torque teno mini virus 7 uid48163 | NC 014082.1 | II |
| 1907 | Torque teno mini virus 8 uid48135 | NC 014068.1 | II |
| 1908 | Torque teno mini virus 9 uid14058 | NC 002195.1 | II |
| 1909 | Torque teno sus virus 1 uid48139 | NC 014070.1 | II |
| 1910 | Torque teno sus virus 2 uid48301 | NC 014092.2 | II |
| 1911 | Torque teno tamarin virus uid48169 | NC 014085.1 | II |
| 1912 | Torque teno virus 1 uid15247 | NC 002076.2 | II |
| 1913 | Torque teno virus 10 uid48151 | NC 014076.1 | II |
| 1914 | Torque teno virus 12 uid48149 | NC 014075.1 | II |
| 1915 | Torque teno virus 14 uid48153 | NC 014077.1 | II |
| 1916 | Torque teno virus 15 uid48191 | NC 014096.1 | II |
| 1917 | Torque teno virus 16 uid48181 | NC 014091.1 | II |
| 1918 | Torque teno virus 19 uid48155 | NC 014078.1 | II |
| 1919 | Torque teno virus 25 uid48165 | NC 014083.1 | II |
| 1920 | Torque teno virus 26 uid48157 | NC 014079.1 | II |
| 1921 | Torque teno virus 27 uid48147 | NC 014074.1 | II |
| 1922 | Torque teno virus 28 uid48145 | NC 014073.1 | II |
| 1923 | Torque teno virus 3 uid48161 | NC 014081.1 | II |
| 1924 | Torque teno virus 4 uid48137 | NC 014069.1 | II |
| 1925 | Torque teno virus 6 uid48187 | NC 014094.1 | II |
| 1926 | Torque teno virus 7 uid48159 | NC 014080.1 | II |
| 1927 | Torque teno virus 8 uid48167 | NC 014084.1 | II |
| 1928 | Tree shrew adenovirus uid14611 | NC 004453.1 | I |
| 1929 | Triatoma virus uid14802 | NC 003783.1 | IV |
| 1930 | Trichodysplasia spinulosa associated polyomavirus uid51185 | NC 014361.1 | I |
| 1931 | Trichomonas vaginalis virus 3 uid14837 | NC 004034.1 | III |
| 1932 | Trichomonas vaginalis virus II uid14822 | NC 003873.1 | III |
| 1933 | Trichomonas vaginalis virus uid14813 | NC 003824.1 | III |
| 1934 | Trichoplusia ni ascovirus 2c uid18003 | NC 008518.1 | I |
| 1935 | Trichoplusia ni SNPV uid15635 | NC 007383.1 | I |

| | | | |
|---|---|---|---|
| 1936 | Triticum mosaic virus uid38495 | NC 012799.1 | IV |
| 1937 | Tulip virus X uid14865 | NC 004322.1 | IV |
| 1938 | Tupaia paramyxovirus uid14723 | NC 002199.1 | V |
| 1939 | Tupaia rhabdovirus uid15415 | NC 007020.1 | V |
| 1940 | Tupaiid herpesvirus 1 uid14597 | NC 002794.1 | I |
| 1941 | Turdivirus 1 uid51587 | NC 014411.1 | IV |
| 1942 | Turdivirus 2 uid51589 | NC 014412.1 | IV |
| 1943 | Turdivirus 3 uid51591 | NC 014413.1 | IV |
| 1944 | Turkey adenovirus A uid14524 | NC 001958.1 | I |
| 1945 | Turkey astrovirus 2 uid14954 | NC 005790.1 | IV |
| 1946 | Turkey astrovirus uid15096 | NC 002470.1 | IV |
| 1947 | Turkey coronavirus uid30039 | NC 010800.1 | IV |
| 1948 | Turnip crinkle virus satellite RNA uid14433 | NC 004033.1 | NA |
| 1949 | Turnip crinkle virus uid14811 | NC 003821.2 | IV |
| 1950 | Turnip crinkle virus virulent satellite RNA C uid14506 | NC 006451.1 | NA |
| 1951 | Turnip curly top virus uid50429 | NC 014324.1 | II |
| 1952 | Turnip mosaic virus uid15408 | NC 002509.2 | IV |
| 1953 | Turnip rosette virus uid14876 | NC 004553.1 | IV |
| 1954 | Turnip vein clearing virus uid14685 | NC 001873.1 | IV |
| 1955 | Turnip yellow mosaic virus uid15293 | NC 004063.1 | IV |
| 1956 | Turnip yellows virus uid15072 | NC 003743.1 | IV |
| 1957 | TYLCAxV Sic1 IT Sic2 2 04  uid30523 | NC 011024.1 | II |
| 1958 | TYLCCNV Y322  satellite DNA beta uid16338 | NC 007735.1 | NA |
| 1959 | UR2 sarcoma virus uid15322 | NC 001618.1 | VI |
| 1960 | Urochloa streak virus uid30033 | NC 010797.1 | II |
| 1961 | Ustilago maydis virus H1 uid14812 | NC 003823.1 | III |
| 1962 | Usutu virus uid15047 | NC 006551.1 | IV |
| 1963 | Vaccinia virus uid15241 | NC 006998.1 | I |
| 1964 | Variola virus uid15197 | NC 001611.1 | I |
| 1965 | Velvet tobacco mottle virus Satellite RNA uid14194 | NC 003906.1 | NA |
| 1966 | Venezuelan equine encephalitis virus uid15302 | NC 001449.1 | IV |
| 1967 | Verbena virus Y uid29881 | NC 010735.1 | IV |
| 1968 | Vernonia yellow vein betasatellite uid41303 | NC 013423.1 | NA |
| 1969 | Vernonia yellow vein virus uid16335 | NC 007730.2 | II |
| 1970 | Vesicular exanthema of swine virus uid14704 | NC 002551.1 | IV |
| 1971 | Vesicular stomatitis Indiana virus uid14673 | NC 001560.1 | V |
| 1972 | Vibrio phage fs1 uid14227 | NC 004306.1 | II |
| 1973 | Vibrio phage fs2 uid14088 | NC 001956.1 | II |
| 1974 | Vibrio phage K139 uid14144 | NC 003313.1 | I |
| 1975 | Vibrio phage kappa uid28503 | NC 010275.1 | I |
| 1976 | Vibrio phage KSF 1phi uid14562 | NC 006294.1 | II |
| 1977 | Vibrio phage KVP40 uid14416 | NC 005083.2 | I |
| 1978 | Vibrio phage N4 uid42785 | NC 013651.1 | I |
| 1979 | Vibrio phage VEJphi uid38367 | NC 012757.1 | II |
| 1980 | Vibrio phage Vf12 uid14385 | NC 005949.1 | II |
| 1981 | Vibrio phage Vf33 uid14384 | NC 005948.1 | II |
| 1982 | Vibrio phage VfO3K6 uid14093 | NC 002362.1 | II |
| 1983 | Vibrio phage VfO4K68 uid14094 | NC 002363.1 | II |
| 1984 | Vibrio phage VGJphi uid14279 | NC 004736.1 | II |
| 1985 | Vibrio phage VHML uid14234 | NC 004456.1 | I |

| | | | |
|---|---|---|---|
| 1986 | Vibrio phage VP2 uid14473 | NC 005879.1 | I |
| 1987 | Vibrio phage VP5 uid14382 | NC 005891.1 | I |
| 1988 | Vibrio phage VP882 uid18851 | NC 009016.1 | I |
| 1989 | Vibrio phage VP93 uid37885 | NC 012662.1 | I |
| 1990 | Vibrio phage VSK uid14337 | NC 003327.2 | II |
| 1991 | Vibriophage VP4 uid15449 | NC 007149.1 | I |
| 1992 | Vibriophage VpV262 uid14316 | NC 003907.2 | I |
| 1993 | Vicia faba endornavirus uid16237 | NC 007648.1 | III |
| 1994 | Visna Maedi virus uid14636 | NC 001452.1 | VI |
| 1995 | Walleye dermal sarcoma virus uid14718 | NC 001867.1 | VI |
| 1996 | Walrus calicivirus uid14874 | NC 004541.1 | IV |
| 1997 | Watermelon mosaic virus uid15046 | NC 006262.1 | IV |
| 1998 | Wesselsbron virus uid38295 | NC 012735.1 | IV |
| 1999 | West Nile virus uid15431 | NC 001563.2 | IV |
| 2000 | West Nile virus uid30293 | NC 009942.1 | IV |
| 2001 | Western equine encephalomyelitis virus uid14831 | NC 003908.1 | IV |
| 2002 | Wheat dwarf virus uid15478 | NC 003326.1 | II |
| 2003 | Wheat eqlid mosaic virus uid20763 | NC 009805.1 | IV |
| 2004 | Wheat streak mosaic virus uid15354 | NC 001886.1 | IV |
| 2005 | White ash mosaic virus uid32671 | NC 011533.1 | IV |
| 2006 | White bream virus uid18013 | NC 008516.1 | IV |
| 2007 | White clover mosaic virus uid15069 | NC 003820.1 | IV |
| 2008 | Wild potato mosaic virus uid15404 | NC 004426.1 | IV |
| 2009 | Wild tomato mosaic virus uid20625 | NC 009744.1 | IV |
| 2010 | Wisteria vein mosaic virus uid15532 | NC 007216.1 | IV |
| 2011 | Wongabel virus uid33129 | NC 011639.1 | V |
| 2012 | Woodchuck hepatitis virus uid14212 | NC 004107.1 | VII |
| 2013 | Woolly monkey sarcoma virus uid19547 | NC 009424.4 | VI |
| 2014 | WU Polyomavirus uid19765 | NC 009539.1 | I |
| 2015 | Xanthomonas phage Cf1c uid14329 | NC 001396.1 | II |
| 2016 | Xanthomonas phage OP1 uid16299 | NC 007709.1 | I |
| 2017 | Xanthomonas phage OP2 uid16300 | NC 007710.1 | I |
| 2018 | Xanthomonas phage phiL7 uid38267 | NC 012742.1 | I |
| 2019 | Xanthomonas phage Xop411 uid19771 | NC 009543.1 | I |
| 2020 | Xanthomonas phage Xp10 uid14292 | NC 004902.1 | I |
| 2021 | Xanthomonas phage Xp15 uid15255 | NC 007024.1 | I |
| 2022 | Xenopus laevis endogenous retrovirus Xen1 uid30173 | NC 010955.1 | VI |
| 2023 | Xestia c nigrum granulovirus uid14092 | NC 002331.1 | I |
| 2024 | Xylella phage Xfas53 uid42595 | NC 013599.1 | I |
| 2025 | Y73 sarcoma virus uid16745 | NC 008094.1 | VI |
| 2026 | Yaba like disease virus uid14595 | NC 002642.1 | I |
| 2027 | Yaba monkey tumor virus uid14466 | NC 005179.1 | I |
| 2028 | Yam mosaic virus uid14884 | NC 004752.1 | IV |
| 2029 | Yellow fever virus uid15284 | NC 002031.1 | IV |
| 2030 | Yersinia pestis phage phiA1122 uid14332 | NC 004777.1 | I |
| 2031 | Yersinia phage Berlin uid18481 | NC 008694.1 | I |
| 2032 | Yersinia phage L 413C uid14280 | NC 004745.1 | I |
| 2033 | Yersinia phage phiYeO3 12 uid14591 | NC 001271.1 | I |
| 2034 | Yersinia phage PY54 uid15227 | NC 005069.1 | I |
| 2035 | Yokose virus uid15118 | NC 005039.1 | IV |

| | | | |
|---|---|---|---|
| 2036 | Youcai mosaic virus uid14869 | NC 004422.1 | IV |
| 2037 | Zantedeschia mild mosaic virus uid32715 | NC 011560.1 | IV |
| 2038 | Zika virus uid36615 | NC 012532.1 | IV |
| 2039 | Zinnia leaf curl disease associated sequence uid14440 | NC 005047.1 | NA |
| 2040 | Zinnia leaf curl virus associated DNA beta uid14538 | NC 005874.1 | NA |
| 2041 | Zucchini green mottle mosaic virus uid15189 | NC 003878.1 | IV |
| 2042 | Zucchini yellow mosaic virus uid15390 | NC 003224.1 | IV |
| 2043 | Zygocactus virus X uid14955 | NC 006059.1 | IV |
| 2044 | Zygosaccharomyces bailii virus Z uid14823 | NC 003874.1 | III |

**Supplementary References**:

40. Makino S, Chang MF, Shieh CK, Kamahora T, Vannier DM et al. (1987) Molecular cloning and sequencing of a human hepatitis delta (delta) virus RNA. Nature, 329: 343-346.

41. Miller JL, Woodward J, Chen S, Jaffer M, Weber B et al. (2011) Three-dimensional reconstruction of Heterocapsa circularisquama RNA virus by electron cryo-microscopy. J. Gen. Virol., 92(8): 1960-1970.
